# Commutability assessment of new standard reference materials (SRMs) for determining serum total 25-hydroxyvitamin D using ligand binding and liquid chromatography–tandem mass spectrometry (LC–MS/MS) assays

**DOI:** 10.1007/s00216-024-05699-7

**Published:** 2025-01-10

**Authors:** Stephen A. Wise, Étienne Cavalier, Pierre Lukas, Stéphanie Peeters, Caroline Le Goff, Laura E. Briggs, Emma L. Williams, Ekaterina Mineva, Christine M. Pfeiffer, Hubert Vesper, Christian Popp, Christian Beckert, Jan Schultess, Kevin Wang, Carole Tourneur, Camille Pease, Dominik Osterritter, Ralf Fischer, Ben Saida, Chao Dou, Satoshi Kojima, Hope A. Weiler, Agnieszka Bielecki, Heather Pham, Alexandra Bennett, Shawn You, Amit K. Ghoshal, Bin Wei, Christian Vogl, James Freeman, Neil Parker, Samantha Pagliaro, Jennifer Cheek, Jie Li, Hisao Tsukamoto, Karen Galvin, Kevin D. Cashman, Hsuan-Chieh Liao, Andrew N. Hoofnagle, Jeffery R. Budd, Adam J. Kuszak, Ashley S. P. Boggs, Carolyn Q. Burdette, Grace Hahm, Federica Nalin, Johanna E. Camara

**Affiliations:** 1 ICF Contractor in Support of National Institutes of Health (NIH), Office of Dietary Supplements (ODS), Bethesda, MD 20817 USA; 2 University of Liège, Clinical Chemistry, CHU de Liège, Liège, BE 4000 Belgium; 3 Imperial College Healthcare NHS Trust, London, W6 8RF UK; 4 Centers for Disease Control and Prevention (CDC), Nutritional Biomarkers Branch, Atlanta, GA 30341 USA; 5 Abbott Laboratories, ADD Wiesbaden Abbott GmbH, 65205 Wiesbaden, Germany; 6 Affimedix Inc., Hayward, CA 94545 USA; 7 BioMérieux, 69280 Marcy-L’Étoile, France; 8 Chromsystems Instruments & Chemicals GmbH, 82166 Gräfelfing, Germany; 9 Diazyme Laboratories, Inc., Poway, CA 92064 USA; 10 Fujirebio Inc., Hachioji-Shi, Tokyo 192-0031 Japan; 11 Nutrition Research Division, Health Canada, Ottawa, K1A 0K9 Canada; 12 Immunodiagnostic Systems (IDS), Boldon, NE35 9PD UK; 13 PerkinElmer Health Sciences, Inc., Hayward, CA 94545 USA; 14 Quest Diagnostics, Valencia, CA 91355 USA; 15 Quest Diagnostics, Chantilly, VA 20151 USA; 16 Roche Diagnostics GmbH, 82377 Penzberg, Germany; 17 Siemens Healthcare Diagnostics Inc., Tarrytown, NY 10591 USA; 18 Siemens Healthcare Diagnostics Inc., Newark, DE 19702 USA; 19 Tosoh Corporation, Kanagawa, 252-1123 Japan; 20 University College Cork, Cork Centre for Vitamin D and Nutrition Research, Cork, T12 YT20 Ireland; 21 Department of Laboratory Medicine, University of Washington, Seattle, WA 98185 USA; 22 Consulting Biostatistician, Shoreview, MN 55126 USA; 23 National Institutes of Health (NIH), Office of Dietary Supplements (ODS), Bethesda, MD 20817 USA; 24 Chemical Sciences Division, National Institute of Standards and Technology (NIST), Gaithersburg, MD 20899 USA

**Keywords:** 25-Hydroxyvitamin D_3_, 25-Hydroxyvitamin D_2_, Vitamin D, Vitamin D-binding protein (VDBP), Immunoassay, Reference measurement procedure (RMP)

## Abstract

Commutability is where the measurement response for a reference material (RM) is the same as for an individual patient sample with the same concentration of analyte measured using two or more measurement systems. Assessment of commutability is essential when the RM is used in a calibration hierarchy or to ensure that clinical measurements are comparable across different measurement procedures and at different times. The commutability of three new Standard Reference Materials^®^ (SRMs) for determining serum total 25-hydroxyvitamin D [25(OH)D], defined as the sum of 25-hydroxyvitamin D_2_ [25(OH)D_2_] and 25-hydroxyvitamin D_3_ [25(OH)D_3_], was assessed through an interlaboratory study. The following SRMs were assessed: (1) SRM 2969 Vitamin D Metabolites in Frozen Human Serum (Total 25-Hydroxyvitamin D Low Level), (2) SRM 2970 Vitamin D Metabolites in Frozen Human Serum (25-Hydroxyvitamin D_2_ High Level), and (3) SRM 1949 Frozen Human Prenatal Serum. These SRMs represent three clinically relevant situations including (1) low levels of total 25(OH)D, (2) high level of 25(OH)D_2_, and (3) 25(OH)D levels in nonpregnant women and women during each of the three trimesters of pregnancy with changing concentrations of vitamin D-binding protein (VDBP). Twelve laboratories using 17 different ligand binding assays and eight laboratories using nine commercial and custom liquid chromatography–tandem mass spectrometry (LC–MS/MS) assays provided results in this study. Commutability of the SRMs with patient samples was assessed using the Clinical and Laboratory Standards Institute (CLSI) approach based on 95% prediction intervals or a pre-set commutability criterion and the recently introduced International Federation of Clinical Chemistry and Laboratory Medicine (IFCC) approach based on differences in bias for the clinical and reference material samples using a commutability criterion of 8.8%. All three SRMs were deemed as commutable with all LC–MS/MS assays using both CLSI and IFCC approaches. SRM 2969 and SRM 2970 were deemed noncommutable for three and seven different ligand binding assays, respectively, when using the IFCC approach. Except for two assays, one or more of the three pregnancy levels of SRM 1949 were deemed noncommutable or inconclusive using different ligand binding assays and the commutability criterion of 8.8%. Overall, a noncommutable assessment for ligand binding assays is determined for these SRMs primarily due to a lack of assay selectivity related to 25(OH)D_2_ or an increasing VDBP in pregnancy trimester materials rather than the quality of the SRMs. With results from 17 different ligand binding and nine LC–MS/MS assays, this study provides valuable knowledge for clinical laboratories to inform SRM selection when assessing 25(OH)D status in patient populations, particularly in subpopulations with low levels of 25(OH)D, high levels of 25(OH)D_2_, women only, or women who are pregnant.

**Graphical Abstract:**

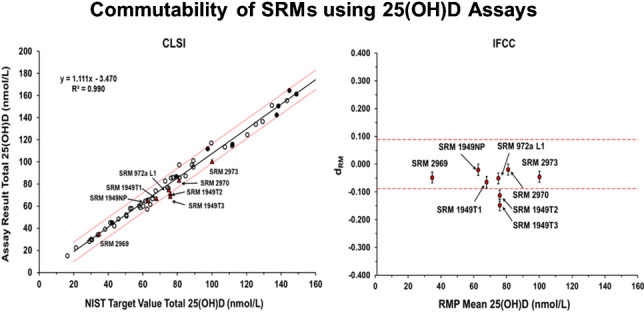

**Supplementary Information:**

The online version contains supplementary material available at 10.1007/s00216-024-05699-7.

## Introduction

The National Institute of Standards and Technology (NIST) and the National Institutes of Health, Office of Dietary Supplements (NIH ODS) have developed Certified Reference Materials (CRMs), denoted as Standard Reference Materials (SRMs^®^), to support measurements of vitamin D metabolites in human serum [1–6]. Clinically, vitamin D status is assessed by measuring total serum 25-hydroxyvitamin D [25(OH)D], which is defined as the sum of 25-hydroxyvitamin D_2_ [25(OH)D_2_] and 25-hydroxyvitamin D_3_ [25(OH)D_3_]. SRM 972 Vitamin D in Frozen Human Serum was issued in 2009 consisting of four different serum pools with certified values assigned for 25(OH)D_2_, 25(OH)D_3_, and 3-*epi*−25-hydroxyvitamin D_3_ [3-*epi*−25(OH)D_3_] [3]. SRM 972 was widely used [6] until it was replaced in 2012 by SRM 972a Vitamin D Metabolites in Frozen Human Serum [4] that also contained four different levels (Table 1). SRM 972a was designed to provide concentrations of serum total 25(OH)D representing both sufficient (20 to 30 ng/mL) and risk of inadequacy (12 to 20 ng/mL) as defined by the Institute of Medicine (IOM) [7, 8], now the National Academy of Medicine (NAM). National health survey assessments for 1988 to 2018 indicate that most of the US population has serum total 25(OH)D concentrations ranging from 16 to 30 ng/mL; however, approximately 35% of the US population have total serum concentrations greater than 30 ng/mL (75 nmol/L) [9–11]. SRM 2973 Vitamin D Metabolites in Human Serum (High Level) was issued in 2017 to complement SRM 972a and to extend the range of total 25(OH)D concentration to 40 ng/mL.

**Table 1:** SRMs for determining vitamin D metabolites^a^

SRM	SRM Level Description	Concentration^a^					
		ng/mL					µg/mL
		25(OH)D_2_	25(OH)D_3_	3-*epi*−25(OH)D_3_	Total 25(OH)D	24,25(OH)_2_D_3_	VDBP
SRM 972a							
Level 1	Normal 25(OH)D	0.54 ± 0.06	**28.8 ± 1.1**	**1.81 ± 0.10**	29.3 ± 1.1^b^	**2.66 ± 0.10**	
Level 2	Low 25(OH)D	**0.81 ± 0.06**	**18.1 ± 0.4**	**1.28 ± 0.09**	**18.9 ± 0.4**	**1.41 ± 0.05**	
Level 3	High 25(OH)D_2_	**13.2 ± 0.3**	**19.8 ± 0.4**	1.17 ± 0.14	**33.0 ± 0.5**	**1.62 ± 0.06**	
Level 4	High 3-*epi*−25(OH)D_3_	0.55 ± 0.10	**29.4 ± 0.9**	**26.0 ± 2.2**	30.0 ± 0.9^b^	**2.64 ± 0.09**	
SRM 2973	High 25(OH)D	0.65 ± 0.02	**39.4 ± 0.8**	2.10 ± 0.08	40.1 ± 0.8^b^	**3.13 ± 0.11**	
SRM 2969	Low 25(OH)D	**2.01 ± 0.05**	**11.9 ± 0.3**		**13.9 ± 0.3**	0.57 ± 0.01^d^	
SRM 2970	High 25(OH)D_2_	**23.5 ± 0.3**	**9.63 ± 0.31**		**33.1 ± 0.4**	0.73 ± 0.01^d^	
SRM 1949							
NP	Nonpregnant	0.67 ± 0.03^d^	24.98 ± 0.28	1.32 ± 0.06	25.65 ± 0.28^b^		211.5 ± 2.8
T1	1st trimester	1.20 ± 0.05	26.01 ± 0.22	1.43 ± 0.02	27.21 ± 0.23^b^		286.7 ± 3.8
T2	2nd trimester	0.514 ± 0.037	30.00 ± 0.50	1.87 ± 0.07	30.51 ± 0.50^b^		349.7 ± 4.3
T3	3rd trimester	0.897 ± 0.057	29.43 ± 0.41	1.87 ± 0.04	30.33 ± 0.41^b^		383.4 ± 5.1

^a^Bold type values are denoted as certified values; normal type values are designated as non-certified or reference values; uncertainties are as stated on the Certificate of Analysis (COA) for each SRM. For specific details for each SRM, see the current COA at www.nist.gov/SRMs and search by SRM number to find the COA

^b^Value for total 25(OH)D is not on the COA; value determined from sum of 25(OH)D_2_ and 25(OH)D_3_ and combined expanded uncertainty

^c^Value for 24,25(OH)_2_D_3_ not reported on the COA; value from certification report by Hahm et al. [2] and converted from ng/g to ng/mL

^d^Value is not on COA

All CRMs for clinical diagnostic markers in human serum, which are typically prepared as pooled and/or processed serum samples, should undergo a commutability assessment to support their equivalent performance to that of individual patient samples [12–14]. The International Vocabulary of Metrology (VIM) defines commutability as “a property of a reference material (RM) (SRM in this study) demonstrated by the closeness of agreement between the relationship among the measurement results for a stated quantity and the relationship obtained among measurement results for other specified materials” (e.g. patient samples in clinical laboratory medicine) [15]. As a practical definition, commutability is where the measurement response for the RM is the same as for an individual patient sample with the same concentration of analyte when measured using two or more measurement systems.

Two previous studies were conducted by NIH ODS, NIST, the Centers for Disease Control and Prevention (CDC), and the University of Ghent (Ghent, Belgium) to assess commutability of SRM 972a and SRM 2973 for determining total 25(OH)D, with the first study conducted in 2011 (SRM 972a) [16] and the second study conducted in 2016 (SRM 972a and SRM 2973) [17]. These previous commutability studies also included external quality assessment (EQA) samples from the Vitamin D External Quality Assessment Scheme (DEQAS) [18] and the College of American Pathologist Accuracy-based Vitamin D (ABVD) scheme [19]. Based on these studies, a RM might be deemed noncommutable for 25(OH)D due to several properties of the sample that may be considered unusual but still relevant clinically. For example, a RM with a high concentration of the 3-epimer might be noncommutable if the LC–MS/MS method does not separate the 25(OH)D_3_ and 3-*epi*−25(OH)D_3_ [20, 21]. Similarly, a material with a high concentration of 25(OH)D_2_ might be noncommutable if the ligand binding assay has unequal response for 25(OH)D_3_ and 25(OH)D_2_ [22, 23]. In both cases, however, the RM is deemed noncommutable (i.e., not behaving like patient samples) because of a lack of selectivity of the assay used and not necessarily the properties of the material. The concentration of vitamin D-binding protein (VDBP) in serum, which increases during pregnancy [6], could also influence the response of a ligand binding assay and thereby alter a commutability assessment [24–27].

Recently, NIST and NIH ODS collaborated to develop three new SRMs with novel properties relative to total 25(OH)D measurements: (1) SRM 2969 Vitamin D Metabolites in Frozen Human Serum (Total 25-Hydroxyvitamin D Low Level), (2) SRM 2970 Vitamin D Metabolites in Frozen Human Serum (25-Hydroxyvitamin D_2_ High Level), and (3) SRM 1949 Frozen Human Prenatal Serum (Four Levels) (Table 1). SRM 2969 has a lower level of total 25(OH)D (i.e., 13.9 ng/mL) than previous SRMs (i.e., 18.9 ng/mL in SRM 972a). The level of 25(OH)D in SRM 2969 is near the NAM threshold at which risk of vitamin D deficiency symptoms increases (i.e., 12 ng/mL) [7, 8], and previous commutability studies did not address this lower level with SRMs or EQA samples [16, 17]. SRM 2970 has a high endogenous 25(OH)D_2_ concentration (23.5 ng/mL), which is higher than that in SRM 972a L3 (13.2 ng/mL) and can appear in individuals consuming vitamin D_2_ dietary supplements. Finally, SRM 1949 is unique in that it is comprised of three prenatal serum levels representing the three trimesters of pregnancy and one from nonpregnant women.

Historically, commutability of CRMs and EQA materials has been assessed following Clinical and Laboratory Standards Institute (CLSI) Guideline EP30-A “Characterization and Qualification of Commutable Reference Material for Laboratory Medicine: Approved Guideline” [28] and EP14 “Evaluation of Commutability of Processed Samples” [29]. In brief, a mathematical relationship between the laboratory results and the reference measurement procedure (RMP) results is established using single-donor samples and results from the RM samples are assessed as to whether they fit within this mathematical relationship, in which case the RMs are deemed commutable. Recently, the International Federation of Clinical Chemistry and Laboratory Medicine (IFCC) published new guidelines for assessing commutability in a series of papers [30–32]. The IFCC approach is based on the difference in bias between a RM and the clinical samples measured using two different measurement procedures, one of which is a RMP in the preferred case for assessing commutability of CRMs. This difference in bias is compared with a commutability criterion, C, which is selected based on a medically relevant difference between the RM and clinical sample results. The advantage of the IFCC approach is that it determines the difference in bias between the RM and the average bias of clinical samples at the measurand concentration in the RM and estimates its uncertainty. Thus, all assays are evaluated in the same manner with the IFCC approach, regardless of their imprecision.

The objective of this study was to assess the commutability of three new SRMs for total 25(OH)D assays using the new IFCC guidelines and to compare the results with the traditional CLSI approach using 95% PI and a pre-set commutability criterion. Assessing commutability for SRM 2969 and 2970 is important because RMs with low levels of 25(OH)D have not been assessed previously, and RMs with high levels of 25(OH)D_2_ have been shown to be noncommutable in previous commutability studies for assays with unequal response to 25(OH)D_2_ and 25(OH)D_3_. Commutability assessment for SRM 1949 would identify potential measurement problems with prenatal serum in determining 25(OH)D using different assays, particularly ligand binding assays, due to changing levels of VDBP. This study also aimed to include a greater number of unique ligand binding assays for total 25(OH)D than in previous studies. A total of 17 different ligand binding assays and nine commercial and custom LC–MS/MS assays were used to assess commutability of six different SRM levels.

## Methods

### Measurands

The measurand for the study was total 25(OH)D in serum in nmol/L, which is defined as the sum of 25(OH)D_2_ and 25(OH)D_3_ and not including 3-*epi*−25(OH)D_3_. For the LC–MS/MS analyses, participants provided results for 25(OH)D_2_, 25(OH)D_3_, and total 25(OH)D, and some participants provided results for 3-*epi*−25(OH)D_3_. Concentrations of 25(OH)D_2_ and 25(OH)D_3_ are typically determined as mass fraction (ng/g) or concentration (ng/mL) and converted to molar concentration (nmol/L) using relative molar masses for 25(OH)D_2_ (412.65 g/mol) and 25(OH)D_3_ (400.64 g/mol) with the equivalent conversion factors of 2.423 and 2.496, respectively.

### Recruitment of participating laboratories

IFCC guidelines [31] recommend including as “many different measurement procedures (MP) and analytical measurement principles as possible in a commutability assessment” and that “including the most representative groups of measurement procedures will increase the likelihood of an RM being suitable for use with other MPs not included in the initial assessment or with a new MP that may enter the market” [31]. The two major assay principles for total 25(OH)D testing are ligand binding assays (primarily immunoassays) and LC–MS/MS assays. For commercially available ligand binding assays, we invited the assay manufacturers’ laboratories to participate in the study rather than a laboratory only using the commercial assay. NIH ODS invited the major assay manufacturers, based on participation in DEQAS and/or in the CDC Vitamin D Standardization—Certification Program (VDSCP) [33]. Invitations to participate were sent to 18 vitamin D assay manufacturers with 10 responding favorably. Because several major assay manufacturers did not participate, we recruited two laboratories (University of Liège, Liège, BE, and Imperial College Healthcare Trust, London, UK) to provide results using additional ligand binding assays. For the LC–MS/MS assays, we targeted commercially available LC–MS/MS systems as well as laboratories using custom assays to provide testing services to the vitamin D research community, including major commercial testing laboratories, clinical research laboratories, and national survey laboratories. We invited 13 laboratories using LC–MS/MS assays and received eight positive responses with one laboratory providing results using two different sample preparation approaches for their assay. Twelve laboratories provided results for 17 different ligand binding assays (25 sets of results) (Table 2), and eight laboratories provided results using nine commercial and custom LC–MS/MS assays (Table 3) for a total of 34 sets of results. There were multiple results for the following ligand binding assays (number of results): Abbott Alinity (3), bioMérieux (2), DiaSorin (2), Fujirebio (2), IDS-iSYS (2), Roche (2), and Siemens ADVIA (2). The participating laboratories included four LC–MS/MS and 13 ligand binding assays that are currently certified assays in the CDC VDSCP [33] as indicated in Tables 2 and 3.

**Table 2:** Participants and ligand binding assays used in this commutability study

Participant	Assay Manufacturer	Assay Kit Name	Assay Instrument/Model	Sample Volume	Assay Type
Abbott Diagnostics	Abbott	Alinity 25-OH Vitamin D*	Alinity i	10 µL	CMIA
Abbott Diagnostics	Abbott	Architect 25-OH Vitamin D*	ARCHITECT i2000SR	10 µL	CMIA
Affimedix Inc	Affimedix	MicrO-D*	SpectraMax ID5 Spectrometer	10 µL	ELISA
bioMérieux	bioMérieux	VIDAS 25 OH Vitamin D Total	VIDAS	100 µL	ELFA
Diazyme Labs Inc	Diazyme	EZ Vitamin D*	Beckman AU680	3 µL	ITA
Fujirebio Inc	Fujirebio	Lumipulse G 25-OH Vitamin D*	LUMIPULSE G1200	20 µL	CLIA
Immunodiagnostic Systems (IDS)	IDS	25-Hydroxy Vitamin D^S^ EIA*		25 µL	EIA
Immunodiagnostic Systems (IDS)	IDS	IDS 25VitD^S^*	IDS-iSYS/IDS i10	10 µL	CLIA
PerkinElmer Health Sciences, Inc	PerkinElmer	Total 25OH Vitamin D ELISA*	80 min Microtiterwell Immunoassay	10 µL	ELISA
Imperial College Healthcare Trust	Abbott	Alinity 25-OH Vitamin D	Alinity i	10 µL	CMIA
Imperial College Healthcare Trust	DiaSorin	25OH Vitamin D Total	Liaison XL	150 µL	CLIA
Roche Diagnostics GmbH	Roche	Elecsys Vitamin D Total III*	Cobas e801	9 µL	ECLIA
Siemens Healthcare Diagnostics	Siemens	Vitamin D Total (VitD)*	ADVIA Centaur XP	20 µL	CLIA
Siemens Healthcare Diagnostics	Siemens	Vitamin D Total (VitD)*	Atellica IM	20 µL	CLIA
Siemens Healthcare Diagnostics	Siemens	LOCI Vitamin D Total*	Dimension/EXL	8 µL	CLIA
Tosoh Corporation	Tosoh	ST AIA-PACK 25-OH Vitamin D*	AIA-2000	60 µL	FEIA
University of Liège	Abbott	Alinity 25-OH Vitamin D	Alinity i	10 µL	CMIA
University of Liège	Beckman Coulter	Access 25(OH) Vitamin D Total	Beckman Coulter Access 2	30 µL	CLIA
University of Liège	bioMérieux	25-OH Vitamin D TOTAL	bioMérieux Mini Vidas	100 µL	ELFA
University of Liège	DiaSorin	25OH Vitamin D Total	Liaison XL	25 µL	CLIA
University of Liège	Fujirebio	Lumipulse G 25-OH Vitamin D	LUMIPULSE G1200	20 µL	CLIA
University of Liège	IDS iSYS	IDS iSYS 25 VTD^S^	IDS iSyS	10 µL	CLIA
University of Liège	Roche	Vitamin D Total III	Cobas E411	15 µL	CLIA
University of Liège	Siemens	Vitamin D Total (VitD)	ADVIA Centaur XP	20 µL	CLIA
University of Liège	Snibe	25-OH Vitamin D	Maglumi X3	10 µL	CLIA

^*^Assay and laboratory currently certified in the CDC Vitamin D Standardization and Certification Program [33]

*CLIA*, chemiluminescence immunoassay; *CMIA*, chemiluminescence microparticle immunoassay; *ELFA*, enzyme-linked fluorescence assay; *FEIA*, fluorescence enzyme immunoassay; *ITA*, immunoturbidimetric assay; *ECLIA*, electrochemiluminescence immunoassay; *ELISA*, enzyme-linked immunosorbent assay; *EIA*, electrochemical immunoassay

**Table 3:** Participants and LC–MS/MS assays used in this commutability study

Participant	Assay [Reference]	Mass Spectrometer and LC column
CDC	LC–MS/MS (modified [34])*	Thermo Altis-Vanquish LC–MS/MS; Supelco Ascentis Express F5, 2.1 mm × 150 mm, 2.7 µm; separates 25(OH)D_3_ and 3-*epi*−25(OH)D_3_
Chromsystems Instruments & Chemicals GmbH	Chromsystems 1 LC–MS/MS (order no. 62062; sample preparation with reaction vials)	Sciex/Citrine Triple Quad; Chromsystems Analytical column (order no. 62130) + Chromsystems Trap Column (order no. 62110/Epi); separates 25(OH)D_3_ and 3-*epi*−25(OH)D_3_
Chromsystems Instruments & Chemicals GmbH	Chromsystems 2 LC–MS/MS (order no. 62062/1000/F; sample preparation with 96 well filter plates	Sciex/Citrine Triple Quad; Chromsystems Analytical column (order no. 62130) + Chromsystems Trap Column (order no. 62110/Epi); separates 25(OH)D_3_ and 3-*epi*−25(OH)D_3_
University College Cork	LC–MS/MS [35]	Waters Acquity TQD; Supelco Ascentis Express F5 (2.1 mm × 100 mm, 2.7 µm); Ascentis Guard column (2.1 mm × 5 mm); separates 25(OH)D_3_ and 3-*epi*−25(OH)D_3_
Health Canada	LC–MS/MS [36]*	Waters XEVO TQ XS; Waters Acquity UPLC HSS PFP, 2.1 mm × 100 mm, 100 Å, 1.8 µm; separates 25(OH)D_3_ and 3-*epi*−25(OH)D_3_
Imperial College Healthcare Trust (ICHT)	LC–MS/MS	Waters Acquity TQ-S Micro; Waters ACQUITY UPLC HSS PFP, 2.1 × 100 mm, 1.8 µm; separates 25(OH)D_3_ and 3-*epi*−25(OH)D_3_
Quest Diagnostics (Chantilly, VA)	LC–MS/MS*^a^	Thermo/TQS Quantum Ultra; Luna C18, 4.6 mm × 50 mm; 5 µm; does not separate 25(OH)D_3_ and 3-*epi*−25(OH)D_3_
University of Liège	LC–MS/MS*	LC–MS/MS QTRAP 6500; Phenomenex Kinetex PFP, 100 Å, 2.1 mm × 100 mm, 2.6 µm; separates 25(OH)D_3_ and 3-*epi*−25(OH)D_3_
University of Washington	LC–MS/MS	Waters/Xevo TQ-XS MS/MS, Acquity UPLC I-Class with a Column Manager; Restek Pentafluorophenyl propyl (PFP Propyl) 180525B 3.0 mm × 100 cm, 5 µm; separates 25(OH)D_3_ and 3-*epi*−25(OH)D_3_

^*^Assay and laboratory currently certified in the CDC Vitamin D Standardization and Certification Program [33]

^a^Assay is certified in CDC VDSCP, but with another Quest laboratory

The IFCC recommends that MPs included in a commutability study must have acceptable performance characteristics for the measurand, such as precision and selectivity [31]. Previous commutability studies and assay performance evaluations [16, 17, 20, 22] have demonstrated that some total 25(OH)D assays have different selectivity for the measurand. No assays were excluded from this study based on either precision or selectivity performance since the study was intended to assess how the SRMs behave with assays of different selectivity.

### Samples

#### Single-donor serum samples

The CLSI guidelines for commutability assessment recommend the use of a minimum of 20 clinical samples [29] and the IFCC guidelines recommend a minimum of 30 samples. We used a set of 50 single-donor serum samples that were analyzed previously in a commutability study in 2016 for SRM 972a and SRM 2973 [17] and that were procured from Solomon Park Research Laboratories (Seattle, WA). Single-donor serum samples from 50 healthy human donors (i.e., no known disease states, pregnant, or renal failure patients) were prepared according to the CLSI C37-A guidelines [37, 38] and contained only endogenous vitamin D metabolites with a distribution of total 25(OH)D concentrations across a clinically relevant range of 15 nmol/L (6 ng/mL) to 150 nmol/L (60 ng/mL). The 50 single-donor samples contained samples from 28 female donors and 22 male donors (see ESM, results spreadsheet). No other demographic information is available for the single-donor samples. The 50 single-donor set included eight samples with elevated levels of 25(OH)D_2_, i.e., > 32 nmol/L (13 ng/mL), which were excluded in some of the evaluations as described later. There were also 12 samples with total 25(OH)D > 100 nmol/L which exceeds the highest concentration of 25(OH)D in the SRMs (SRM 2973). Commutability assessment using the CLSI 95% PI approach was performed for the remaining 38 samples to evaluate whether this influenced the commutability assessment using all 50 single-donor samples. Each single-donor sample vial contained 0.5 mL of serum. The preparation and distribution of 25(OH)D concentrations for these 50 single-donor samples were described previously [20].

#### SRM samples

The commutability of SRM 2969, SRM 2970, and SRM 1949 (Table 1) was assessed in this study. These SRMs were prepared from serum pools from multiple donors. For SRM 1949, the four levels were based on the following donors: nonpregnant (NP) women of reproductive age (*n* = 12), first trimester (T1) women 6 to 12 weeks pregnant (*n* = 40), second trimester (T2) women 18 to 21 weeks (*n* = 69), and third trimester (T3) women 32 to 35 weeks pregnant (*n* = 60) [1]. The mean donor age for each serum pool in SRM 1949 was 29 years. No additional demographic information on the donors for SRM 1949 was available, and no demographic data was available for donors used to prepare SRM 2969 or SRM 2970. For five of the 25(OH)D ligand binding assays that were not included in the previous commutability study [17] (Affimedix, Diazyme, Fujirebio, PerkinElmer, and Tosoh), SRM 972a L1 and SRM 2973 were also included in this study.

## Study experimental design and analysis protocol

### Participant laboratory analysis protocol

The experimental design of the study was based on IFCC guidance for the commutability assessment [30–32]. The IFCC approach recommends the analysis of patient samples using two MPs. For each MP, the patient samples are analyzed in one run with replicate measurements of the patient samples in adjacent positions, i.e., one after the other, and the RM samples located in five distinct positions within the run order. The sequence of the clinical samples is randomly assigned by concentrations, and the RM samples are in various positions, with adjacent replicates, throughout the analysis sequence. The minimum number of replicates is two and the recommended minimum number of positions for each RM is five [31, 32].

For this study, participant laboratories analyzed duplicate preparations of each of the 50 single-donor serum samples (DS-01 through DS-50) in adjacent positions in one run. The six SRM levels, with adjacent replicates, were distributed among the single-donor samples (separated by either two or four single-donor samples). Using this protocol, the participant laboratories performed a total of 160 sample measurements. The five participant laboratories analyzing the two additional SRMs provided a total of 180 measurements. Laboratories were provided with the analysis protocol and a data reporting template (Excel spreadsheet). The sample analysis protocol including the recommended run order is provided in the Electronic Supplementary Material (ESM). Samples were distributed to the participating laboratories in October 2022, and most of the results were received in December 2022 with two laboratories providing results in March 2023.

#### Participant laboratory assays

Each participating laboratory provided information regarding the assay used (Tables 2 and 3). For the ligand binding assays, information on assay kit, assay instrument/model, and sample volume required for analysis is provided (Table 2). For the LC–MS/MS methods, the LC–MS/MS instrumentation and analytical LC column (Table 3) and LC conditions, internal standards, and *m/z* transitions monitored (Table S1) are summarized. For the assay manufacturers providing results from multiple assays (Abbott, IDS, Siemens, and Chromsystems Instrument & Chemicals), a brief explanation of differences in a manufacturer’s multiple assays is provided in the ESM. Different ligand binding assays may have differing responses for 25(OH)D_2_ and 25(OH)D_3_ and cross-reactivity for 3-*epi*−25(OH)D_3_ and/or 24,25(OH)_2_D_3_ as reported by the manufacturers and/or by studies comparing various ligand binding assays (see Tables S2 and S3, ESM). The results for all laboratories for the 50 single-donor samples and the SRMs are provided in an Excel spreadsheet in the ESM.

#### NIST analysis of single-donor samples and SRMs

NIST analyzed the 50 single-donor samples previously [20] in duplicate using the RMPs for 25(OH)D_2_ [39], 25(OH)D_3_ [39], and 24,25(OH)_2_D_3_ [40], and these measurements were used to assign target concentrations in the current commutability study. In the previous study, subsamples (≈2 g from combining contents from four vials each containing 0.5 mL of serum) were prepared and were analyzed twice (duplicate injections) by LC–MS/MS [20]; however, for this study, only the first injection of each of the two replicates was used to mimic the protocol followed by the other laboratories (Table S4, ESM).

For the SRMs, replicate measurements performed during NIST’s certification campaign using the RMPs were used as the assigned target values in this study representing the five RM positions in the analysis protocol. The certification analyses for SRM 2969 and SRM 2970 [2], SRM 2973 [5], and SRM 972a [4] are described elsewhere. For the certification analyses, subsamples from 12 or 13 vials were analyzed; however, only results from 10 vials were used to represent the duplicates in the five RM positions in the study analysis protocol. In addition, subsamples were analyzed twice (duplicate injections) in the certification analyses whereas in this study, only the first injection of each of the two replicates was used.

For SRM 1949, there were two modifications to the experimental design. Firstly, the NIST RMPs were not used in the value assignment of 25(OH)D_2_ and 25(OH)D_3_, and the assigned values were denoted as reference values (i.e., non-certified) rather than certified values [1]. The NIST ID LC–MS/MS method used for analysis of SRM 1949 was a modification of the RMPs intended to provide a higher throughput method to determine 25(OH)D_2_, 25(OH)D_3_, and 3-*epi*−25(OH)D_3_ in one chromatographic run using different isotopically labeled internal standards as described previously [1, 6] rather than the RMPs, which are based on separate chromatographic runs for the determination of 25(OH)D_2_ and 25(OH)D_3_. Secondly, only three sets of replicate measurements (rather than five) were available for the analysis of SRM 1949, which necessitated modification of some of the statistical equations to reflect three rather than five RM positions in the data evaluation protocol. For SRM 1949, duplicate subsamples (750 µL) from each of three vials (each containing 1.8 mL of serum) of the four levels were prepared and analyzed with two injections each. Only the first injection was used for the commutability study data analysis. The measurement replicates used to assign the target values for 25(OH)D_2_, 25(OH)D_3_, and total 25(OH)D in the five SRMs analyzed in this commutability study are summarized in Tables S5 and S6 (ESM).

#### Distribution of samples to participants

Each participant received a panel of 50 single-donor serum (DS) samples (50 vials each containing 0.5 mL of serum) and one vial of each of the three NIST SRMs: (1) SRM 2969 (1.1 mL of serum/vial), (2) SRM 2970 (1.1 mL of serum/vial), and (3) SRM 1949 (four levels) (1.8 mL of serum/vial for each level). Several laboratories analyzed the samples using more than one assay and were therefore provided with additional sets of the 50 single-donor samples and the SRMs as needed. Samples were shipped frozen on dry ice. Upon receipt of the samples, participants were requested to store them frozen (at − 60 °C or lower) until analyzed.

## Data analysis

Participant results were evaluated using three different approaches for commutability assessment: (1) the traditional CLSI approach using 95% PIs, (2) the CLSI assessment using a pre-set offset commutability criterion, and (3) the IFCC approach using the difference in bias. In the CLSI approach using 95% PIs [29], the commutability is determined by comparing the measured results for the SRM samples to the scatter of the results for the 50 patient samples using the laboratory measurement procedure and the RMPs. Using the Ordinary Deming Regression model, the mean results of the duplicate measurements for the 50 single-donor samples for the laboratory MP and the RMPs are used to provide a regression line. The scatter is represented by a 95% PI about the regression line. If the mean value of the replicate measurements for the SRMs is within the 95% PI, the SRM is deemed commutable; if the mean is outside the 95% PI, the SRM is deemed noncommutable. All calculations for the Ordinary Deming Regression were performed using Analyse-it (Analyse-it Software, Leeds, UK). The Analyse-it Methods Comparison Tab, which is based on CLSI EP14-Ed4 and EP30A guidelines [28, 29], was used to generate the Ordinary Deming Regression line with 95% PI based on the single-donor samples and determines whether the SRMs are within or outside the 95% PI. The Ordinary Deming Regression requires input of the ratio of variances of the RMP versus the test assay (*λ*). As in the previous commutability study [17], we used *λ* = 0.1 as an approximate mean of individual values and for consistency for all the assays (see [17] for discussion of the selection of the *λ* value). Using the Analyse-it Methods Comparison function for commutability assessment eliminated potential bias associated with visual examination of plots to assess commutability.

The recent update of CLSI EP14 in 2022 includes a second approach to assess commutability using a pre-set offset rather than the 95% PI. This alternative approach avoids an incorrect commutability assessment when the comparison plot of the two MPs results in a very narrow distribution for patient samples around the regression line indicating high precision and analytical selectivity. In such a case, a noncommutability bias may be observed when there is none (i.e., a type I error). To minimize this possibility, the CLSI guidelines state that a criterion “based on a clinically acceptable bias can be pre-set around the regression fit” [29]. We plotted the constant offset using 8.8% as the clinically significant criterion as discussed below.

Whereas the CLSI approaches for commutability assessment are based on the statistical distribution of the measurements for the single-donor samples observed for the two MPs, the IFCC approach for commutability assessment is based on differences in bias between the SRM and the clinical samples measured using the two different MPs. The IFCC approach was developed to provide a commutability assessment that is independent of variability of assay measurements (i.e., all measurement procedures are assessed using the same criterion). The difference between the bias for the RM and the average bias for the single-donor samples is denoted as *d*_RM_ and is an estimate of the closeness of the agreement between the bias for the RM and the bias for the single-donor samples and the expanded uncertainty of the estimate *U*(*d*_RM_) [32]. A maximum value of ǀ*d*_RM_ǀ for the RM to be considered commutable is established, which is designated as the commutability criterion, C [32]. The RM is commutable when *d*_RM_ ± *U*(*d*_RM_) is within 0 ± C, noncommutable when *d*_RM_ ± *U*(*d*_*RM*_) is outside 0 ± C, and inconclusive when *d*_RM_ ± *U*(*d*_RM_) and 0 ± C overlap [32]. In presenting the IFCC approach for commutability, Nilsson et al. [32] provided an Excel template as Supplementary Material with example calculations and step by step instructions to determine *d*_RM_ ± *U*(*d*_RM_). We used this template after making the necessary changes to accommodate our modifications to the experimental design including duplicate analyses rather than triplicate analyses and measurements for only three RM positions for SRM 1949 rather than five RM positions (see ESM for Modified IFCC template).

After evaluating our results as described above, CLSI released EP30 Ed2 in August 2024 [41] which updated the 95% PI approach outlined in EP14 [29] to include confidence intervals around the RM points in the direction orthogonal to the regression line to “help users understand the confidence they can place on the study results” [41]. In addition, the new EP30 Ed2 [41] now includes and recommends the IFCC differences in bias approach. Because our study focuses on the IFCC difference in bias approach for assessment of commutability and compares the results to previous studies using the traditional CLSI 95% PI approach, we have not evaluated our results with the addition CIs to the RM points as recommended in the recent update. We acknowledge that the lack of CI on the RM points may limit a comparison to the differences in bias approach; however, it does not limit our intended comparison to previous studies using the CLSI approach.

### Selection of commutability criterion (C)

A critical decision in the IFCC approach [32] and the recent CLSI pre-set offset approach [29] is selection of a commutability criterion, C, to establish the bias limits for the assessment. A number of recent commutability studies for various clinical biomarkers [42–55] have implemented the IFCC approach for assessing RMs, control materials, and/or EQA materials (Table S7, ESM for details) and compared the IFCC differences in bias approach with the traditional CLSI 95% PI approach. In these studies, the value for C ranged from 4 to 23.7% and was selected based on biological variability [46, 51, 52, 54–56], recommended guidelines of international/national organizations [42, 44, 47, 48, 50], or performance of routine methods [49, 53].

Establishing analytical performance specifications for the measurement of 25(OH)D has been debated for over a decade [57, 58]. Sandberg et al. [59] and Ceriotti et al. [60] proposed criteria for assigning measurands to three different models for analytical performance specifications (APS) based on the following: (1) the effect of APS on clinical outcome, (2) components of biological variation, and (3) state-of-the-art measurements. For determining biological variation, a European Federation of Clinical Chemistry and Laboratory Medicine meta-analysis includes five studies for 25(OH)D_3_ [61–64]. Viljoen et al. [64] reported analytical quality goals for 25(OH)D measurements based on biological variation of 12.1% within subject and 40.3% between subject and calculated the critical difference for sequential values to be 38.4% (*p* < 0.05). A recent study by Cavalier et al. [61] demonstrated that an APS for the measurement of 25(OH)D based on biological variation was inappropriate and proposed an APS based on measurement uncertainty (MU). Analytical methods that would differentiate a change in 25(OH)D induced by vitamin supplementation should have an MU < 13.6% (*p* < 0.05) [61]. In deriving the proposed MU of 13.6%, Cavalier et al. [61] used a value of 31.6% for the physiological variation of the 25(OH)D concentration over a 10-week period based on measurements using an immunoassay with very high precision (1.5% CV) comparable to their LC–MS/MS method.

Recently, Miller et al. [65] provided recommendations from the IFCC Working Group on Commutability in Metrological Traceability regarding the selection of a quantitative criterion to assess commutability of CRMs. The commutability criterion is denoted as the maximum allowable noncommutability bias (MANCB) that would allow a CRM to be used as a calibrator in a calibration hierarchy for a 25(OH)D assay without exceeding the maximum allowable combined standard uncertainty for a clinical sample result (*u*max_CS_). Miller et al. [65] proposed assigning 3/8 of the *u*max_CS_, which is the maximum allowable *u* from noncommutability, *u*max_NC_. The MANCB is then derived as a fraction of the *u*max_NC_ using the equation MANCB = √3 × *u*max_NC_. If we use the MU value of 13.6% from the study of Cavelier [61] as the value for *u*max_CS_, then *u*max_NC_ is 5.1% (3/8 × 13.6%) and MANCB becomes 8.8% (1.73 × 5.1%). We therefore used 8.8% for C for the CLSI pre-set limit and IFCC approaches.

## Results and discussion

### Traditional CLSI approach for assessment of commutability

For the traditional CLSI approach to assess commutability, the Ordinary Deming Regression was used to establish the relationship of measurement results obtained for the 50 single-donor samples using the 25(OH)D assay and results obtained using the NIST RMPs with 95% PIs. A potential limitation of this approach is that if the assay has high measurement variability, the 95% PI is broad and the SRMs may be assessed as commutable (inside the 95% PI) when they are noncommutable. Conversely, if the assay has high precision, then the 95% PI may be too narrow and indicate that an SRM is noncommutable (i.e., outside the 95% PI) when it is, in fact, commutable.

**Fig. 1:**
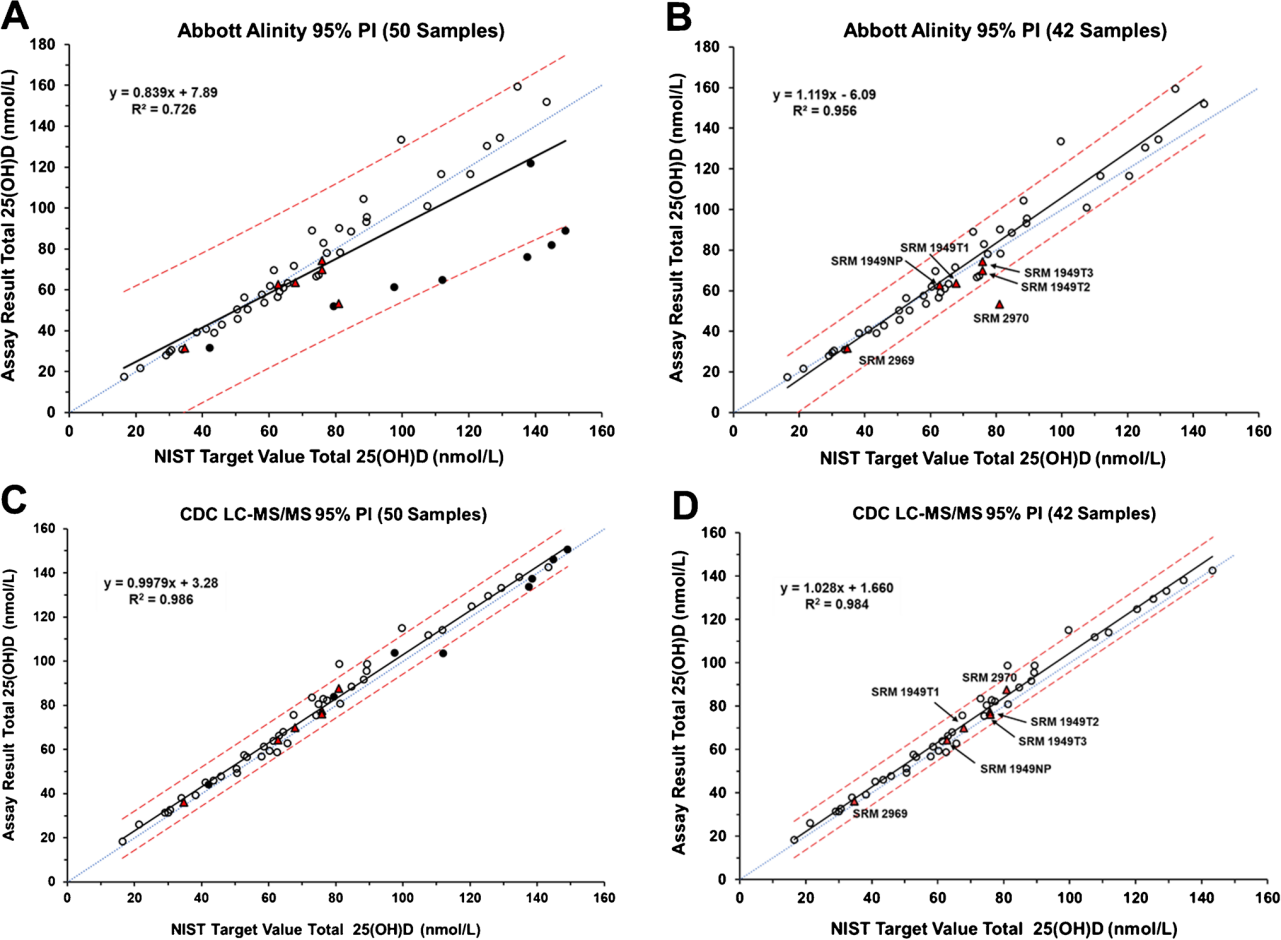
Assessment of commutability using the CLSI 95% PI approach for (**A** and **B**) Abbott Alinity assay and (**C** and **D**) CDC LC–MS/MS assay using both the 50 and 42 single-donor sets. The 42-sample set excluded eight samples with elevated 25(OH)D_2_ concentrations (i.e., > 20 nmol/L). The black circles (open and filled) are the single-donor samples. The black-filled circles represent the single-donor samples with high 25(OH)D_2_ concentrations. The black solid line is the Ordinary Deming regression line and the red dashed lines are the 95% PI. The blue dotted line is the identity line (*y* = *x*). The red triangles are the SRM samples which are identified in the plots for the 42-sample set

In the previous commutability study for 25(OH)D using this same set of 50 single-donor samples [17], the commutability assessment was performed using the traditional CLSI 95% PI approach with both the 50 single-donor sample set and a subset of 42 samples, i.e., excluding the samples with high 25(OH)D_2_ concentrations (32 to 137 nmol/L), due to the variability of the measurements for several ligand binding assays. For the current study, we have also performed the commutability assessment with both the 50 and 42 single-donor sample sets for direct comparison to the earlier study [17]. The results for the Ordinary Deming Regression line (slope, intercept, *R*^2^) and the PI (minimum and maximum *y*-intercept value and width) are summarized in Table S8 (ESM) and demonstrate a significant change in slope of the regression line and a significant decrease in PI width for several of the ligand binding assays (up to 60%) between the 50- and 42-sample sets. However, the LC–MS/MS assays do not change significantly when using either the 50- or the 42-sample set. These two assay behaviors using the CLSI 95% PI approach are illustrated in Fig. 1 for the 50 and 42 single-donor sample sets for the Abbott Alinity assay and for the CDC LC–MS/MS assay. Based on the 95% PI plot in Fig. 1A using all 50 single-donor samples, all SRMs would be assessed as commutable using the Abbott Alinity assay; however, when using the 42-sample subset, SRM 2970 is deemed to be noncommutable. For the CDC LC–MS/MS assay (Fig. 1C and D), all SRMs are assessed as commutable regardless of whether the 50- or 42-sample set is used for the 95% PI with similar results for the remaining LC–MS/MS assays (Table S9, ESM). Using the CLSI 95% PI approach with the 50-sample set for the ligand binding assays, only two SRMs (SRM 1949T2 and SRM 1949 T3) would be assessed as noncommutable for three assays (Table S10, ESM). However, when using the 42-sample subset, SRM 2970 would also be assessed as noncommutable (Fig. 1B) for Abbott Alinity assay and for six additional assays (Table S11, ESM). Regression analysis plots showing both the 50- and 42-sample sets for the remaining ligand binding and LC–MS/MS assays are provided in Figs. S1 through S16, and the assessment outcomes are summarized in Tables S9, S10, and S11 (ESM).

The 50 single-donor sample set included 12 samples with total 25(OH)D concentrations greater than 100 nmol/L. Since the SRMs evaluated for commutability all had concentrations less than 100 nmol/L, we also investigated whether the removal of these 12 samples would significantly alter the commutability assessment using CLSI 95% PI approach. The results of the Ordinary Deming regression analysis for the 38-sample set are summarized in Table S12. Because six of the eight samples with high 25(OH)D_2_ concentration were part of the 12 samples removed from the evaluation, the commutability assessment results are similar to the evaluation using the 42-sample set.

**Fig. 2:**
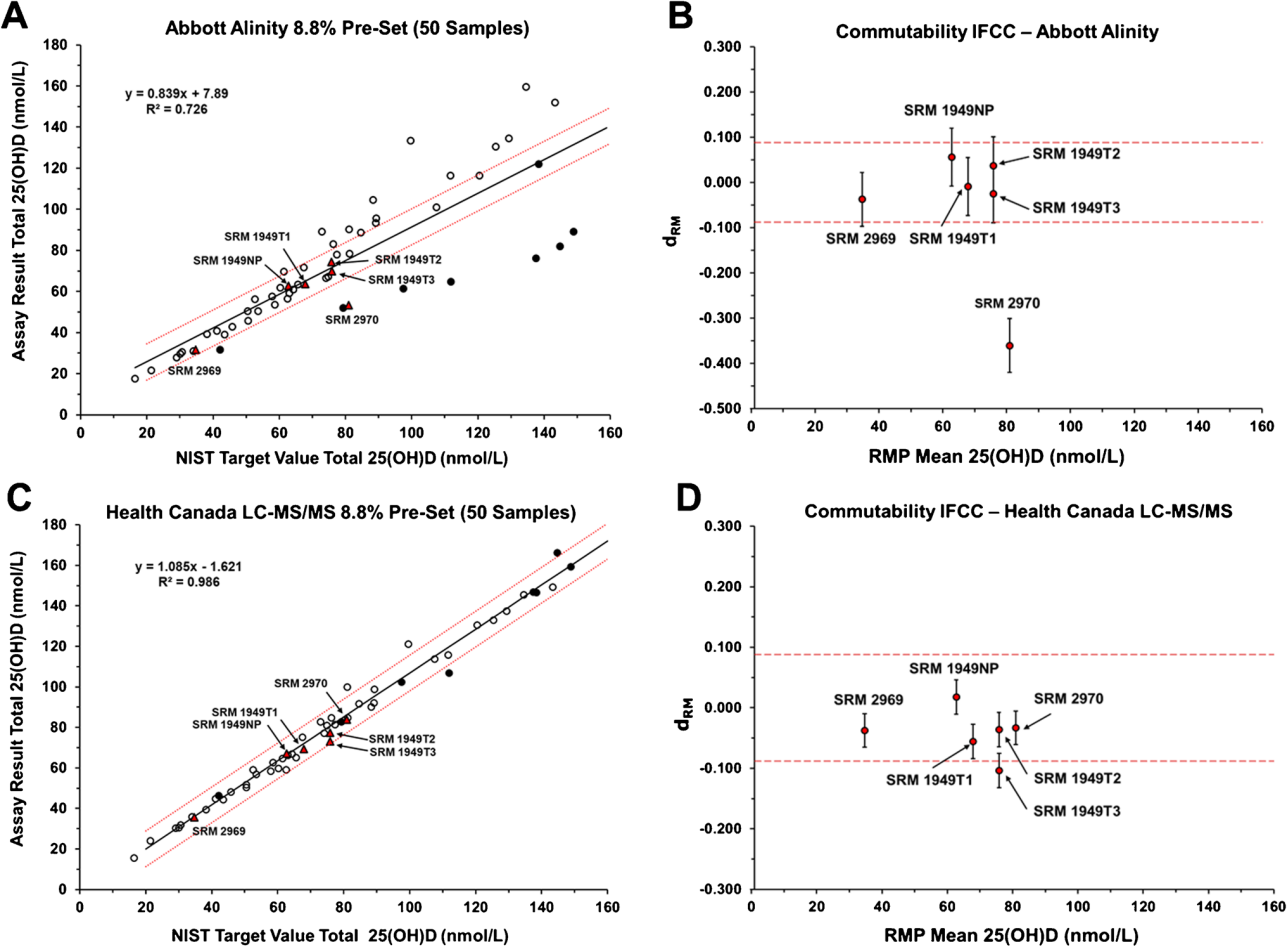
Comparison of commutability assessment using the CLSI pre-set limit approach and IFCC approach both with C = 8.8% using (**A** and **B**) Abbott Alinity assay and (**C** and **D**) Health Canada LC–MS/MS assay. For **A** and **C**, the black circles (open and filled) are the single-donor samples. The black-filled circles represent the single-donor samples with high 25(OH)D_2_ concentrations. The black solid line is the Ordinary Deming regression line and the red dotted lines are the 8.8% pre-set limits. The red triangles are the SRM samples. For B and D, the red dashed lines are ± the commutability criterion (C) of 8.8%. The red-filled circles are the *d*_RM_ values and the error bars are the expanded uncertainty, *U*(*d*_RM_), which is a 95% confidence interval for the *d*_RM_ values

To address this limitation of the CLSI 95% PI approach, the recent edition of CLSI EP14 [29] suggests using a constant offset above and below the regression line as a pre-set criterion that “may be based on a clinically significant difference.” For evaluation of results of this study, we selected the same value for the pre-set criterion as the C used in the IFCC approach, namely 8.8%. The results for the Abbott Alinity assay using this approach are shown in Fig. 2A indicating that SRM 2970 is noncommutable (Table S13, ESM). For LC–MS/MS assays, the CLSI pre-set offset approach provides assessments like the CLSI 95% PI approach because the measurement variability of the LC–MS/MS results is generally low, 0.5 to 5.2% based on five replicate measurements for each SRM (Table S14, ESM). The results for the remaining assays using the CLSI pre-set offset approach are provided as Figs. S17 through S22 (ligand binding assays) and Figs. S23 and S24 (LC–MS/MS assays). The advantage of both CLSI approaches (95% PI and pre-set offset) is that the regression plot of the individual measurements provides an easy visualization of the commutability assessment.

### IFCC approach for assessment of commutability

**Fig. 3:**
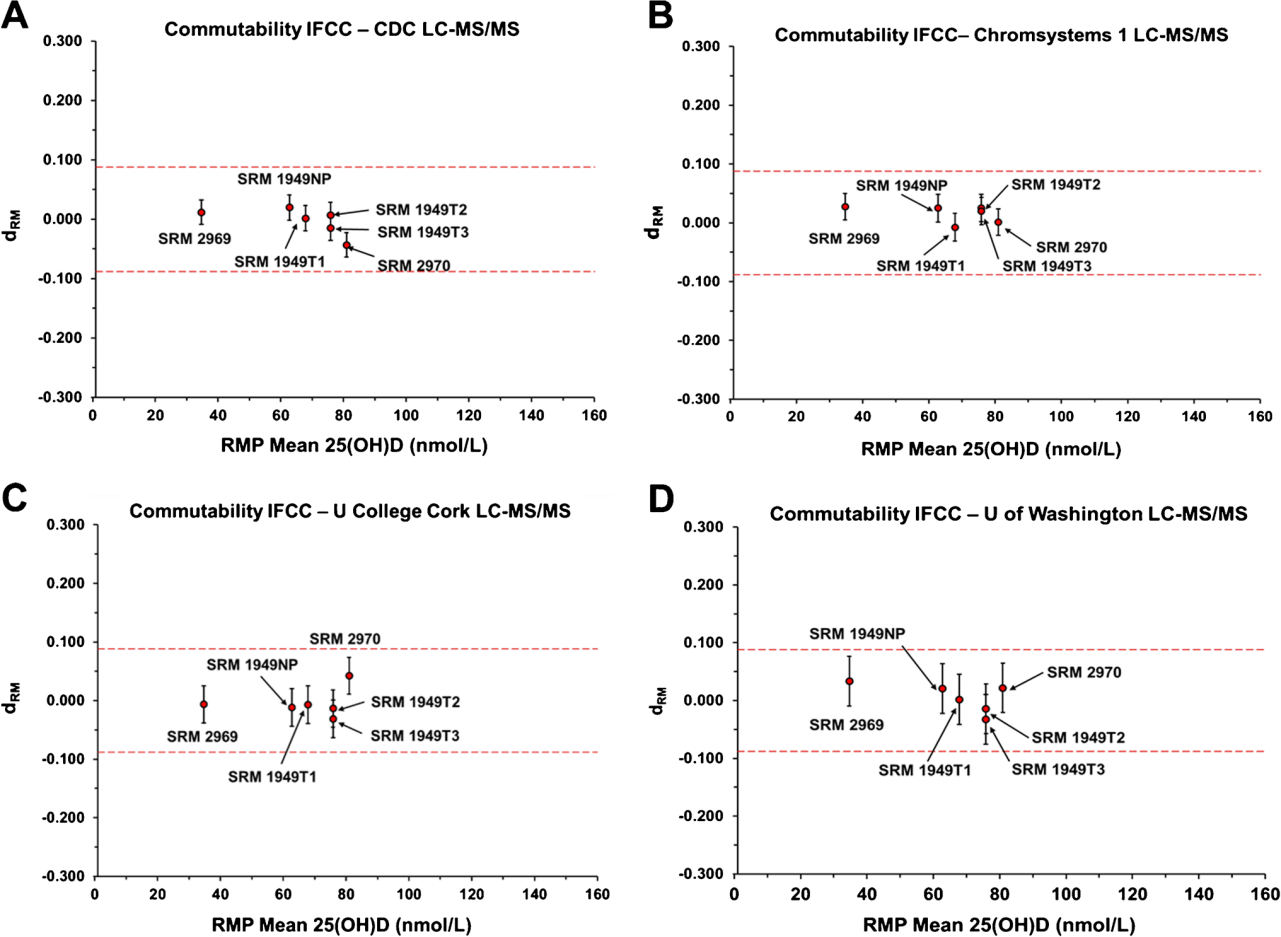
Assessment of commutability using the IFCC approach for LC–MS/MS assays: (**A**) CDC, (**B**) Chromsystems 1, (**C**) University College Cork, and (**D**) University of Washington. Red dashed lines are ± the commutability criterion (C) of 8.8%. The red-filled circles are the *d*_RM_ values and the error bars are the expanded uncertainty, *U*(*d*_RM_), which is a 95% confidence interval for the *d*_RM_ values

**Fig. 4:**
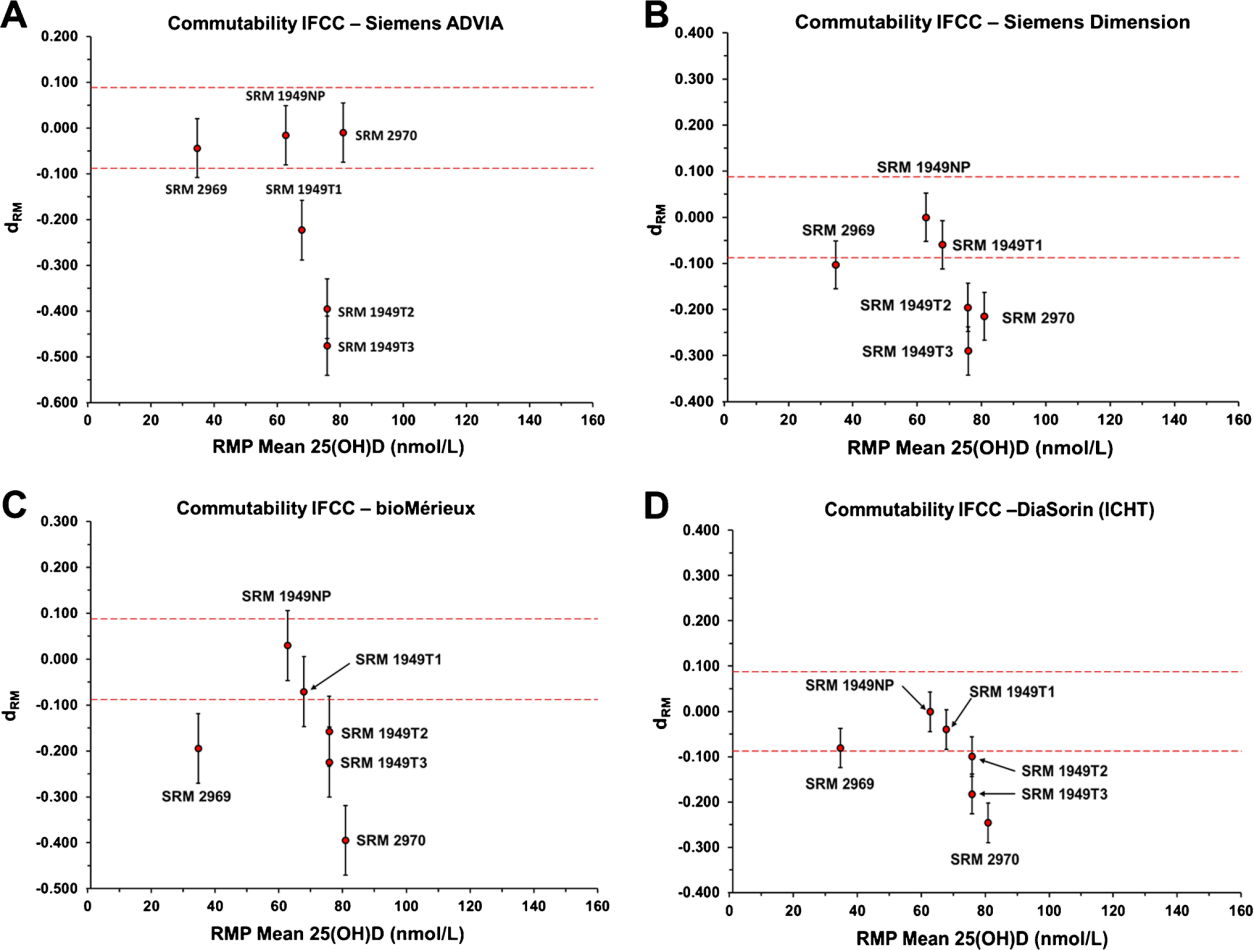
Assessment of commutability using the IFCC approach for selected ligand binding assays with focus on SRM 2970: (**A**) Siemens ADVIA, (**B**) Siemens Dimension, (**C**) bioMérieux, and (**D**) DiaSorin (IHCT). Red dashed lines are ± the commutability criterion (C) of 8.8%. The red-filled circles are the *d*_RM_ values and the error bars are the expanded uncertainty, *U*(*d*_RM_), which is a 95% confidence interval for the *d*_RM_ values

**Fig. 5:**
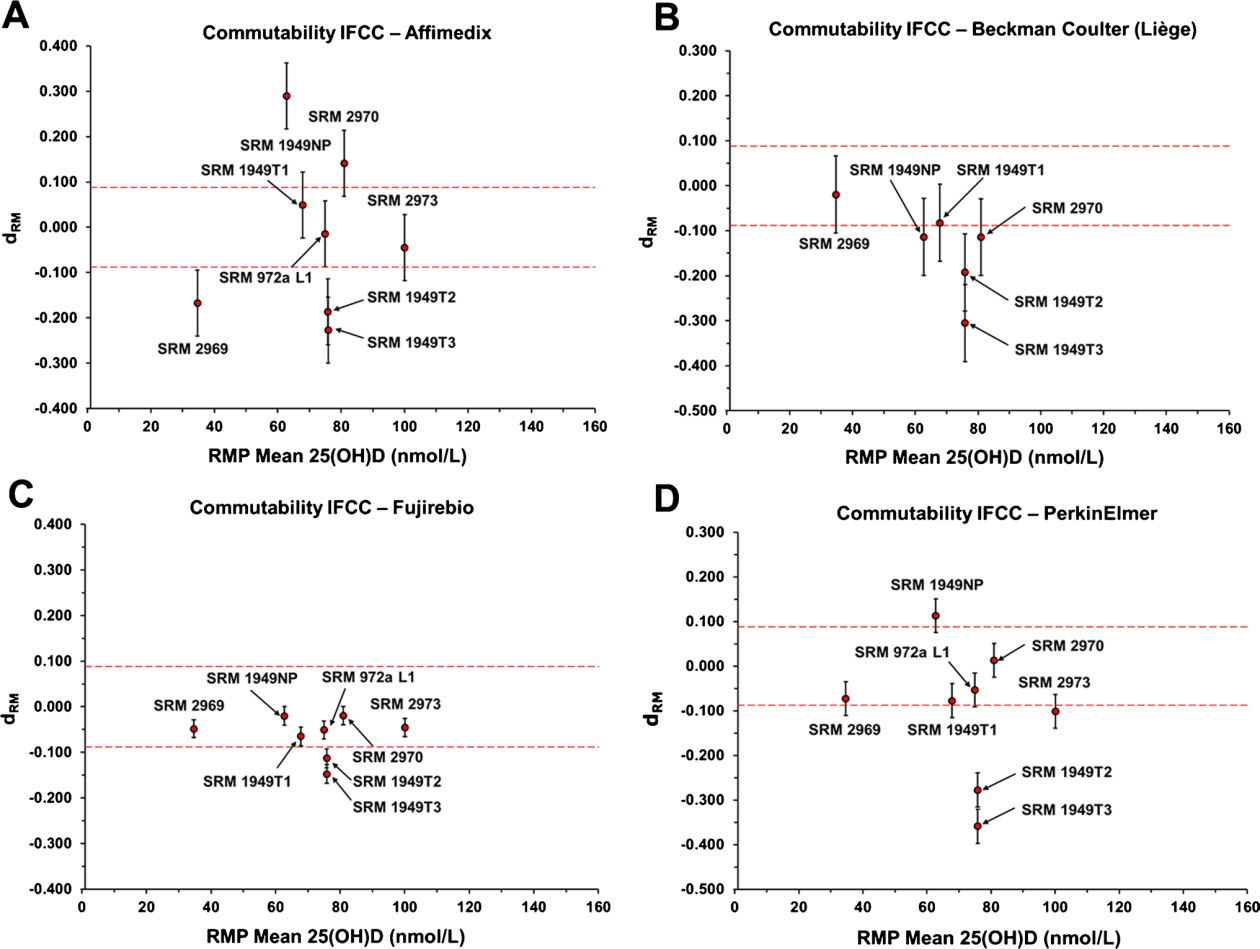
Assessment of commutability using the IFCC approach for selected ligand binding assays with focus on SRM 1949: (**A**) Affimedix, (**B**) Beckman Coulter, (**C**) Fujirebio, and (**D**) PerkinElmer. Red dashed lines are ± the commutability criterion (C) of 8.8%. The red-filled circles are the *d*_RM_ values and the error bars are the expanded uncertainty, *U*(*d*_RM_), which is a 95% confidence interval for the *d*_RM_ values

All assay results for the single-donor samples and SRMs were evaluated using the IFCC approach, and the commutability plots of *d*_RM_ versus concentration determined by the RMP were prepared using a commutability criterion of 8.8%. Selected commutability plots are provided in Fig. 3 for four LC–MS/MS assays and in Figs. 4 and 5 for eight ligand binding assays.

#### Inconclusive commutability assessment

Using the IFCC approach, the assessment of commutability is deemed to be “inconclusive” when the confidence interval of the d_RM_ overlaps with the commutability criterion indicating that the experiment did not provide an unequivocal decision of either commutable or noncommutable compared with the clinical samples. If the assay imprecisions are large and the commutability criterion small, then an inconclusive commutability assessment will be more frequent. Recent recommendations by the IFCC provide further considerations on how to determine whether a CRM is fit-for-purpose when the commutability assessment is inconclusive [65]. Miller et al. [65] provide six possible cases of inconclusive commutability assessments and offer recommendations on whether the CRM will be fit-for-purpose in a calibration hierarchy. In case 1, the mean noncommutability bias is within C with only a small fraction of the error bar exceeding C as for SRM 1949NP (Fig. 4C, bioMérieux) and SRM 1949T1 (Fig. 4B). In this example, the relatively small fraction of the confidence interval (CI) exceeding C would probably not cause the umaxcs to be exceeded. In case 2 (Fig. 5D, PerkinElmer), SRM 2969 has about the same noncommutability bias as in example 1 (Fig. 4C, SRM 1949NP) but with a larger fraction of the CI outside the C boundaries. With the larger uncertainty in case 2, the umaxcs is likely exceeded. Cases 3 and 4 are analogous to examples 1 and 2 except that the noncommutability bias is outside the 8.8% C value as shown in Fig. 5D, PerkinElmer) for SRM 1949NP and SRM 2973. Cases 5 and 6 are illustrated in Fig. 4C (bioMérieux) for SRM 1949NP, when the noncommutability bias is small (i.e., near 0) and the CI is large but only a small fraction exceeds the C value, and for SRM 1949T2 when the noncommutability bias is large (i.e., outside C) and the CI is sufficiently large to overlap the C boundary.

The IFCC recommendations suggest that the location of the mean noncommutability bias within or outside the C value can resolve an inconclusive assessment if the overlapping portion of the uncertainty is relatively small. If the mean noncommutability bias for the SRM is inside the C value with only a small portion of the uncertainty outside the C value, then the SRM is highly likely commutable and useful in a calibration hierarchy. Similarly, if the mean noncommutability is outside the C value with only a small portion of the uncertainty inside the C value, then the SRM is highly likely noncommutable. When a significant portion of the uncertainty is outside the C value even if the mean noncommutability is inside, the inconclusive assessment would make it difficult to justify using the SRM in a calibration hierarchy. Likewise, an analogous situation with mean noncommutability bias outside the C value but significant overlap of the uncertainty inside would also make it difficult to use in a calibration hierarchy. In Tables 4 and 5, we have arbitrarily denoted 25% of the error bar overlap as the “the relatively small fraction” from the IFCC guidelines and color-coded the results to indicate where an inconclusive assessment is likely to be commutable or noncommutable.

**Table 4:**
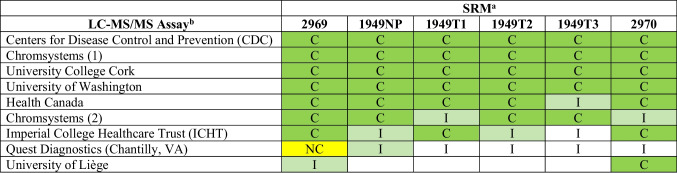
Commutability assessment of SRMs for LC–MS/MS assays using IFCC approach with C = 8.8%

^a^*C*, commutable (green); *NC*, noncommutable (yellow); *I*, inconclusive. For an Inconclusive assessment of commutability, light textured green indicates that the *d*_RM_ value is within the C boundaries (± 8.8%) and* Ud*_RM_ overlaps the C boundary less than 25% outside, and light textured yellow indicates that the d_RM_ value is outside the C boundaries (± 8.8%) and *Ud*_RM_ overlaps the C boundary less than 25%. No color for an Inconclusive assessment indicates that the overlap of *Ud*_RM_ is greater than 25% for *d*_RM_ values either inside or outside the C boundaries (± 8.8%). Blank cells indicate that the sample was not analyzed

^b^Assays ordered to cluster assays with similar assessment of the SRMs

**Table 5:**
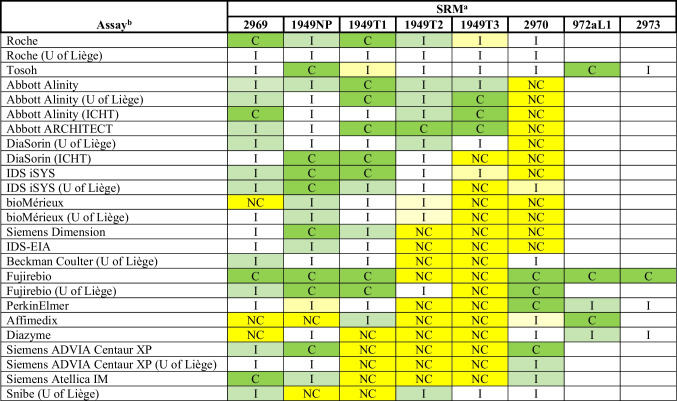
Commutability assessment of SRMs for ligand binding assays using IFCC approach with C = 8.8%

^a^*C*, commutable (green); *NC*, noncommutable (yellow); *I*, inconclusive. For an Inconclusive assessment of commutability, light textured green indicates that the *d*_RM_ value is within the C boundaries (± 8.8%) and *Ud*_RM_ overlaps the C boundary less than 25% outside, and light textured yellow indicates that the *d*_RM_ value is outside the C boundaries (± 8.8%) and *Ud*_RM_ overlaps the C boundary less than 25%. No color for an Inconclusive assessment indicates that the overlap of *Ud*_RM_ is greater than 25% for *d*_RM_ values either inside or outside the C boundaries (± 8.8%). Blank cells indicate that the sample was not analyzed

^b^Assays ordered to cluster assays with similar assessment of the SRMs

#### LC–MS/MS assays

Plots of *d*_RM_ versus the concentration of 25(OH)D determined by the RMP illustrating the IFCC approach to commutability assessment are shown in Fig. 3 for four LC–MS/MS assays with uncertainties associated with the *d*_RM_ values (i.e., the error bars) varying from 0.0206 to 0.0427 (Table S15). An assessment of all the SRMs as commutable using LC–MS/MS assays is expected (Table 4, Fig. 3 and Fig. S25), unless the LC–MS/MS assay does not separate the 25(OH)D_3_ and the 3-*epi*−25(OH)D_3_ and the concentration of 3-*epi*−25(OH)D_3_ is high as was demonstrated in a previous study [17] for SRM 972a L4. In the current study, however, only one LC–MS/MS assay did not chromatographically separate the 25(OH)D_3_ and the 3-*epi*−25(OH)D_3_ (Table 3). Using the IFCC approach, several SRMs were assessed as inconclusive with five of the nine LC–MS/MS assays (Table 4), whereas both CLSI approaches would have assessed all SRMs as commutable (Table S9 and S14).

#### Ligand binding assays

Commutability assessment plots for selected ligand binding assays are provided in Figs. 4 and 5 with uncertainties for the *d*_RM_ values ranging from 0.0198 to 0.1352 (Tables S16 and S17, ESM), and the assessment for 17 ligand binding assays is summarized in Table 5. SRM 2970 is of particular interest because it has a high endogenous concentration of 25(OH)D_2_, which has not been assessed in SRMs in previous commutability studies, i.e., 23.5 ng/mL (56.9 nmol/L) vs. 13.2 ng/mL (32.0 nmol/L) in SRM 972a L3 [17]. In the previous commutability study [17], SRM 972a L3 and two EQA samples with high levels of 25(OH)D_2_ were assessed as noncommutable for several ligand binding assays (Abbott ARCHITECT, bioMérieux, DiaSorin, IDS-iSYS, and Snibe). In this study, SRM 2970 was assessed as noncommutable by seven different assays (Table 5) with the assessment plots for five of these assays shown in Fig. 2B and Fig. 4 (B, C, and D) and assessment plots for the remaining ligand binding assays provided in Figs. S26 through S29 (ESM). SRM 2970 was also deemed noncommutable using the Abbott ARCHITECT assay (Fig. S26C), which is like the Abbott Alinity, and the IDS EIA assay (Fig. S28B). SRM 2970 was assessed as commutable using only three assays including Fujirebio (Fig. 5C), PerkinElmer (Fig. 5D), and Siemens ADVIA Centaur XP (Fig. 4A), and the remaining seven assays were deemed as inconclusive (Table 5 and Figs. S26 through S29). Le Goff et al. [23] reported that the Abbott ARCHITECT assay had 64% cross-reactivity for 25(OH)D_2_ whereas the Siemens ADVIA Centaur XP assay overestimated 25(OH)D_2_ by 30%. SRM 2969, which contains a low concentration of total 25(OH)D, was assessed as commutable using only three assays including Fujirebio, Roche, and Siemens Atellica IM (with Abbott Alinity commutable in only 1 of 3 data sets) (Table 5) and as noncommutable using the Affimedix (Fig. 5A), bioMérieux (Fig. 4C), and Diazyme (Fig. S27B) assays. All other assays were inconclusive in the assessment of SRM 2969 (Figs. S26 through S29).

For SRM 1949, only the Abbott ARCHITECT (Fig. S26C) and the Abbott Alinity assays (Fig. 2B and Fig. S26), which use the same reagents, assessed all three pregnancy levels in SRM 1949 as commutable (one level was inconclusive with only slight overlap of the C value). All three pregnancy levels in SRM 1949 were deemed noncommutable using the Diazyme (Fig. S27B), Siemens ADVIA Centaur XP (Fig. 4A and S29A), and Siemens Atellica IM (Fig. S29B) assays (Table 5). Two of the three pregnancy levels (SRM 1949T2 and SRM 1949T3) were deemed noncommutable using six assays, i.e., Affimedix, Beckman Coulter, Fujirebio, IDS-EIA, PerkinElmer, and Siemens Dimension assays (Figs. 5A, 5B, 5C, S28B, 5D, and 4B, respectively). The Snibe assay deemed only SRM 1949T1 as noncommutable among the pregnancy levels. The remaining two assays (Roche and Tosoh) were inconclusive in the assessment of SRM 1949 pregnancy levels (Table 5, Figs. S28C and S29D). Cavalier et al. [25] evaluated the Fujirebio, DiaSorin, IDS-iSYS, and Roche assays for various patient populations including 3rd trimester pregnant women (*n* = 30) and found the agreement with LC–MS/MS to be moderate, poor, poor, and substantial, respectively. The assessment of the various pregnancy trimester levels of SRM 1949 as noncommutable using all but two assays is particularly noteworthy. The serum pools used to produce other SRMs for determining 25(OH)D have excluded pregnant women as donors. As demonstrated by the assigned values for VDBP in SRM 1949 (Table 1) [1, 66], the concentration of VDBP increases from nonpregnant women through the three trimesters of pregnancy (Fig. S30, ESM). Interestingly, SRM 1949NP, which consists of serum from only women donors and has a total 25(OH)D concentration similar to SRM 972a L1 (a normal level), which has serum from both men and women donors, was assessed as noncommutable using two assays, Affimedix and Snibe, and inconclusive (tending to noncommutable) for the PerkinElmer assay. This study represents the first report of a commutability study and the accompanying performance evaluation of various total 25(OH)D assays using SRMs specifically designed to contain serum from pregnant women and from women only.

Cavalier and coworkers [24–26] reported that several 25(OH)D immunoassays behave poorly when compared to a reference LC–MS/MS assay for serum from 3rd trimester pregnant women, including DiaSorin, Beckman Coulter, Abbott ARCHITECT, Roche, and IDS-iSYS. In this study, SRM 1949T3 was assessed as noncommutable (or inconclusive) using these same assays except for Abbott ARCHITECT (Table 5). Interestingly, Cavalier et al. [25] noted that the Fujirebio assay was in “moderate” agreement with the LC–MS/MS assay for samples from 3rd trimester women; however, in our study, SRM 1949T3 was assessed as noncommutable using the Fujirebio assay. Recently, Zhang et al. [27] observed that four investigated immunoassays underestimated the 25(OH)D content in pregnant women including Roche (− 28.3%), DiaSorin (− 39.8%), and Siemens ADVIA Centaur XP (− 50.6%), which is consistent with the degree of noncommutability demonstrated in this study with all three pregnancy levels of SRM 1949 using these assays. Siemens ADVIA Centaur XP assay (Fig. 4A) provided an assessment of noncommutable for all three trimester levels; DiaSorin provided an assessment of inconclusive and noncommutable for SRM 1949T2 and SRM 1949T3, respectively (Fig. 4D); and Roche provided an assessment of commutable for SRM 1949T1 and inconclusive for SRM 1949T2 and SRM 1949T3 (Fig. S28C and S28D and Table 5).

### Comparison of IFCC approach with the CLSI approaches — limitations and advantages

For the LC–MS/MS assays, the different approaches for assessing commutability provide similar results in that the SRMs were found to be commutable (Tables S9 and S14, ESM), although there were several inconclusive assessments using the IFCC approach (Table 5). For the ligand binding assays, however, the IFCC and CLSI 95% PI provide significantly different assessments for the SRMs depending on the assay. If we use all 50 single-donor samples for the CLSI 95% PI approach, all SRMs would be commutable for all assays with a few exceptions (Table S10), i.e., SRM 1949T2 and SRM 1949T3 (Fujirebio, PerkinElmer, Siemens ADVIA Centaur XL). These exceptions occur for the assays that are not significantly affected by the eight samples with high 25(OH)D_2_ (i.e., the width of the PI does not change significantly between the 50- and 42-sample sets). Using the CLSI approach with 95% PI, excluding the eight samples with high 25(OH)D_2_ concentrations (42-sample set Table S11), provides a more realistic assessment of commutability with a greater number of noncommutable assessments: SRM 2970 (six assays), SRM 1949T3 (five assays), and SRM 1949T2 (one assay). The IFCC approach (Table 5) provides the most noncommutable assessments: SRM 1949T3 (12 assays), SRM 1949T2 (9 assays), SRM 2970 (6 assays) 1949T1 (4 assays), SRM 1949NP (2 assays), and SRM 2969 (2 assays) with the remaining IFCC assessments deemed as inconclusive. Thus, the IFCC approach is significantly more stringent in determining commutability. However, when the inconclusive assessments are further evaluated using the IFCC recommendations (i.e., small overlap of error bars), a significant number tend toward commutable or noncommutable as assessed with the CLSI approach (42 sample set and 8.8% pre-set limit). The CLSI pre-set limit approach using the IFCC value for C of 8.8% provides similar assessments compared to the IFCC approach (Fig. 2, Table 5) but with far fewer inconclusive assessments (Table S13, ESM). In a letter to the editor comparing their results from a commutability assessment of frozen serum pools for measurements of HDL and LDL cholesterol with a similar study, Delatour et al. [67] suggested that medical-based criteria to determine C are probably too stringent. Overall, for 25(OH)D ligand binding assays, the IFCC approach provides an accurate assessment of commutability. The characterization of the IFCC approach as too stringent is contingent on assay precision and of course selectivity. Perhaps the greatest challenge in the use of the IFCC approach is the determination of an appropriate commutability criterion.

### Comparison with previous commutability studies

Two previous commutability studies for SRMs and EQA samples for 25(OH)D assays have been coordinated by NIH ODS, NIST, CDC, and the University of Ghent [16, 17]. In the first study [16], participants assessed SRM 972a using 18 assays; however, only results from six ligand binding assays and three LC–MS/MS assays were reported. For the second study [17], results from 34 laboratories using 11 different ligand binding assays and 14 LC–MS/MS assays were used to assess commutability of SRM972a and SRM 2973 as well as 15 EQA samples including an assessment of nine EQA samples shipped frozen versus ambient temperature [68]. The current commutability study is therefore the most extensive study for 25(OH)D assays to date with 17 unique ligand binding assays, including eight ligand binding assays not previously evaluated, as well as nine LC–MS/MS assays to assess three new SRMs comprising six levels of 25(OH)D. In addition, this study had participation from 11 commercial assay manufacturers (including one commercial LC–MS/MS assay) using 15 different assays compared to only seven assay manufacturers using eight different assays in the previous study (including one commercial LC–MS/MS).

The current study addressed four RM situations that had not previously been addressed in commutability studies for 25(OH)D in SRMs including serum: (1) with a lower level of total 25(OH)D, (2) with a higher level of 25(OH)D_2_, (3) from women only, and (4) from pregnant women representing each trimester. For the lower level of total 25(OH)D, the previous study included SRM 972a L2, which has a total 25(OH)D concentration of 18.9 ng/mL and was found to be commutable for all ligand binding assays. In this study using the IFCC approach, SRM 2969 [13.9 ng/mL 25(OH)D] was assessed as noncommutable using two ligand binding assays (bioMérieux and Diazyme), commutable for seven assays, and inconclusive for eight assays indicating that the low levels of total 25(OH)D may be problematic for some ligand binding assays.

SRM 2970 contains 23.5 ng/mL of 25(OH)D_2_, which is a significantly higher concentration than assessed for SRMs in the previous studies (i.e., 13.2 ng/mL 25(OH)D_2_ in SRM 972a L3). However, there were two EQA samples in the previous study at nominally the same concentration of 25(OH)D_2_ as in SRM 2970. As observed in the previous study [17], several assays assessed SRM 972a L3 and EQA samples with high 25(OH)D_2_ concentrations, as noncommutable (i.e., Abbott ARCHITECT, bioMérieux, DiaSorin, IDS-iSYS, and Snibe). In the current study, using the IFCC approach, SRM 2970 was assessed as commutable for only three assays (Fujirebio, PerkinElmer, and Siemens ADVIA Centaur XL), as noncommutable for seven assays (Abbott Alinity and ARCHITECT, bioMérieux, DiaSorin, IDS-EIA, IDS-iSYS, and Siemens Dimension), and as inconclusive for the seven remaining assays (Table 5). Interestingly, in the current study using the same CLSI 95% PI approach as in the previous study, the IDS-iSYS assay was assessed as commutable (Table S11, ESM).

### Multiple variable regression analysis

As with assay results from a previous intercomparison/commutability study [20, 22] and from a study of DEQAS samples [69], we performed multiple variable regression analysis on the 25(OH)D results from the various assays to assess the contributions of 25(OH)D_2_, 25(OH)D_3_, 3-*epi*−25(OH)D_3_, and 24,25(OH)_2_D_3_ to assay response for total 25(OH)D (Table 6 for ligand binding and Table S18 LC–MS/MS assays). Multiple regression analysis of similar data sets from the previous commutability study [20, 22] identified several ligand binding assays that underestimate the response of 25(OH)D_2_. For this commutability study, the same assays were found to underestimate the response of 25(OH)D_2_ by 14 to 52% including Abbott (Alinity and ARCHITECT), bioMérieux, DiaSorin, Diazyme, IDS-EIA, IDS-iSYS, and Snibe. Of these eight assays, the four with significant underestimation (> 30%) for 25(OH)D_2_ (Abbott, bioMérieux, and IDS-EIA) were the assays for which the assessment of SRM 2970 (i.e., high 25(OH)D_2_ content) was noncommutable. In the previous study, multiple variable regression indicated that several LC–MS/MS assays were influenced by 3-*epi*−25(OH)D_3_ and overestimated the contribution of 25(OH)D_2_. In this study, multiple variable regression demonstrated that no LC–MS/MS assays were influenced significantly by the 3-epimer because only one assay did not separate the 25(OH)D_3_ and the 3-*epi*−25(OH)D_3_ (Table S18) and the level of 3-*epi*−25(OH)D_3_ in the SRMs was not significant.

**Table 6:**
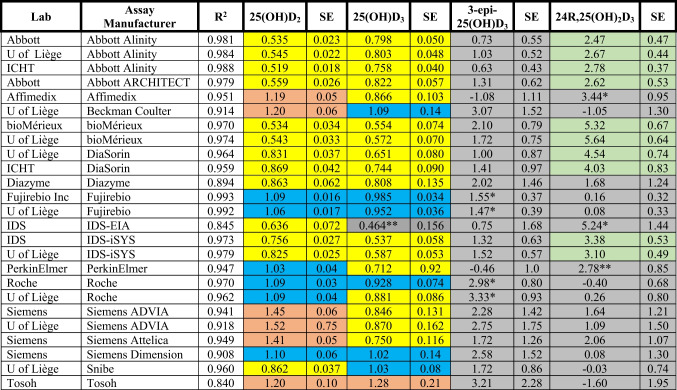
Multivariable linear regression analysis for ligand binding assays for 50 single-donor samples

Color Key for *X*_1_, *X*_2_, *X*_1_, and *X*_4_ from multivariable regression equation:

Estimated as expected (0.9 to 1.1) and significant contribution to estimate (*p* < 0.0001)

Underestimated (< 0.9) and significant contribution to estimate (*p* < 0.0001)

Overestimated (> 1.1) and significant contribution to estimate (*p* < 0.0001)

Significant contribution to the estimate (*p* < 0.0001)

No significant contribution to the estimate (*p* > 0.0001)

^*^Indicates contribution to the estimate (0.0001 > *p* < 0.001)

^**^Indicates contribution to the estimate (*p* < 0.005)

### Commutable versus noncommutable — is it the SRM or the assay selectivity?

As stated in the IFCC recommendations: “MPs to be included in a commutability assessment must have adequate selectivity for the measurand. MPs with inadequate selectivity could inappropriately disqualify an RM that may be suitable for use with many MPs being used in clinical laboratories.” In this study, all available assays were included, even those assays that are known to have sub-optimal selectivity for 25(OH)D_2_ and cross-reactivity with other metabolites such as 3-*epi*−25(OH)D_3_ and 24,25(OH)_2_D_3_ (Table S2). Overall, it appears that the SRMs are commutable, meaning they do behave like clinical patient samples, and that an observed noncommutability assessment can be attributed to a lack of selectivity (specificity) of the response of some ligand binding assays rather than the quality of the SRMs. As stated by Miller et al. [70], “Lack of specificity is a potential limitation for any analytical procedure, but it is of particular importance for immunoassays, in which antibody specificities (e.g., for various epitopes of an analyte) can differ among measurement procedures. Nonspecificity for the analyte found in native clinical samples is a method limitation distinct from noncommutability influences, but it can be a confounding factor when the commutability of a reference material is being validated among methods.” SRM 1949 clearly presents a challenge for nearly all ligand binding assays, presumably due to the higher VDBP during pregnancy (see Fig. S30).

While a majority of the ligand binding (15 of 25) and LC–MS/MS (5 of 9) assays do meet the criteria of < ± 5% bias [33, 71] compared to the NIST RMP target values (see Tables S18 and S19, ESM), only two of the LC–MS/MS and none of the ligand binding assays have an absolute mean % bias of < 5%. Most ligand binding assays in this study (Table S18, ESM) also show low individual sample pass rates (i.e., individual sample measurements < ± 5% bias) of 8 to 40% (only one assay had > 50%) for the 50 single-donor samples, which may be due to lack of selectivity for the ligand binding assays (Table S2). This observation is consistent with results from the CDC VDSCP in which participants demonstrate assay performance relative to the criterion of < ± 5% bias compared to the CDC RMP for 25(OH)D on a sample set of 40 patient samples analyzed on a quarterly basis [33]. As stated in the CDC list of Certified Total 25-hydroxyvitamin D Assays [33], the ± 5% mean bias criterion “can be considered the allowable calibration bias” and “Certification indicates that the assay is calibrated to meet those limits. Due to differences in test selectivity, measurements on individual samples can exceed calibration bias. Therefore, the individual sample pass rate provides some information about selectivity of a test that meets the calibration criteria.” [33]. Pass rates for assays in this study agree with the median and are within the ranges of results for the same assays participating in the CDC VDSCP. Although ligand binding assays with lower sample pass rates (< 30%) appear more likely to assess the SRMs as noncommutable, the inclusion of SRM 1949 in the study complicates that evaluation since the Fujirebio assay with the highest pass rate (i.e., 54% of samples with bias < ± 5%) assesses SRM 1949T2 and SRM 1949T3 as noncommutable. Only the Abbott Alinity and ARCHITECT assays, which are based on similar measurement principles, assessed all three trimester levels of SRM 1949 as commutable with the Roche and Tosoh assays providing inconclusive rather than noncommutable assessments. The LC–MS/MS assays have pass rates ranging from 22 to 76% in this study which are generally lower than the CDC VDSCP pass rates for the same LC–MS/MS assays but within the range of reported VDSCP results. Overall, these observations indicate that the SRMs are of good quality and the noncommutable determination is due to a lack of assay selectivity related to 25(OH)D_2_ or the increasing VDBP in the pregnancy trimester materials.

## Conclusions

This commutability assessment study is the first for total 25(OH)D assays using the IFCC approach for evaluation, and it includes the most diverse set of ligand binding assays to date. In addition, the SRMs assessed represent novel, but clinically relevant patient subpopulations, i.e., with low levels of 25(OH)D, high levels of 25(OH)D_2_ due to supplementation, nonpregnant women only, and women during pregnancy. LC–MS/MS assays provide consistent assessment of all SRMs as commutable using both the CLSI and IFCC evaluations indicating that the SRMs are of suitable quality for clinical 25(OH)D measurements. However, all three SRMs with novel properties relative to 25(OH)D measurements were deemed noncommutable using the IFCC approach by the majority of the 17 different ligand binding and to a lesser extent with the CLSI 95% PI and pre-set limit approaches. The high concentration of 25(OH)D_2_ in SRM 2970 presents a significant challenge for many of the ligand binding assays as demonstrated by the noncommutable assessment using seven assays. SRM 2969 with its low concentration of total 25(OH)D also presents a challenge for a limited number of the ligand binding assays as demonstrated by three noncommutable and a multitude of inconclusive assessments using the IFCC approach. Except for two similar ligand binding assays, one or more of the three pregnancy levels of SRM 1949 was assessed as noncommutable (or inconclusive) indicating that the response of most ligand binding assays may be influenced by the increasing levels of VDBP in women during pregnancy.

## Supplementary Information

Below is the link to the electronic supplementary material.

Supplementary file1 (DOCX 7.10 MB)

Supplementary file2 (DOCX 184 KB)

Supplementary file3 (XLSX 160 KB)

Supplementary file4 (XLS 304 KB)

## Declarations

### Disclaimer

The opinions expressed in this article are the authors’ own and do not reflect the view of the National Institutes of Health, the Department of Health and Human Services, or the United States government. Any citations of commercial organizations and trade names in this report do not constitute an official NIH, NIST, or CDC endorsement of approval of the products or services of these organizations.
