# Supplementary material for: Commutability assessment of new standard reference materials (SRMs) for determining serum total 25-hydroxyvitamin D using ligand binding and liquid chromatography–tandem mass spectrometry (LC–MS/MS) assays
Source: Anal Bioanal Chem. 2025 Jan 10;417(12):2539–61. doi: 10.1007/s00216-024-05699-7 (PMC12003064; doi:10.1007/s00216-024-05699-7)

**Supplemental Information**

**Tables and Figures**

**Commutability Assessment of New Standard Reference Materials (SRMs) for Serum Total 25-Hydroxyvitamin D using Ligand Binding and Liquid Chromatography – Tandem Mass Spectrometry (LC-MS/MS) Assays**

Stephen A. Wise^a^, Étienne Cavalier^b^, Pierre Lukas^b^, Stéphanie Peeters^b^, Caroline Le Goff^b^, Laura E. Briggs^c^, Emma L. Williams^c^, Ekaterina Mineva^d^, Christine M. Pfeiffer^d^, Hubert Vesper^d^, Christian Popp^e^, Christian Beckert^e^, Jan Schultess^e^, Kevin Wang^f^, Carole Tourneur^g^, Camille Pease^g^, Dominik Osterritter^h^, Ralf Fischer^h^, Ben Saida^i^, Chao Dou^i^, Satoshi Kojima^j^, Hope A. Weiler^k^, Agnieszka Bielecki^k^, Heather Pham^l^, Alexandra Bennett^l^, Shawn You^m^, Amit K. Ghoshal^n^, Bin Wei^o^, Christian Vogl^p^, James Freeman^q^, Neil Parker^q^, Samantha Pagliaro^q^, Jennifer Cheek^r^, Jie Li^r^, Hisao Tsukamoto^s^, Karen Galvin^t^, Kevin D. Cashman^t^, Hsuan-Chieh Liao^u^, Andrew N. Hoofnagle^u^, Jeffery R. Budd^v^, Adam J. Kuszak^w^, Ashley S. P. Boggs^x^, Carolyn Q. Burdette^x^, Grace Hahm^x^, Federica Nalin^x^, and Johanna E. Camara^x^

^a^ Scientific Consultant, ICF contractor in support of National Institutes of Health (NIH), Office of Dietary Supplements (ODS), Bethesda, MD 20817, USA

^b^ University of Liège, Clinical Chemistry, CHU de Liège, 4000 Liège, BE

^c^ Imperial College Healthcare NHS Trust, London W6 8RF, UK

^d^ Centers for Disease Control and Prevention (CDC), Nutritional Biomarkers Branch, Atlanta, GA 30341, USA

^e^ Abbott Laboratories, ADD Wiesbaden Abbott GmbH, Wiesbaden 65205, DE

^f^ Affimedix Inc., Hayward, CA 94545, USA

^g^ BioMérieux, 69280 Marcy‑l’Étoile, FR

^h^ Chromsystems Instruments & Chemicals GmbH, 82166 Gräfelfing, DE

^i^ Diazyme Laboratories, Inc., Poway, CA 92064, USA

^j^ Fujirebio Inc., Hachioji-shi, 192-0031Tokyo, JP

^k^ Health Canada, Nutrition Research Division, Ottawa K1A 0K9, CAN

^l^ Immunodiagnostic Systems (IDS), Boldon NE35 9PD, UK

^m^ PerkinElmer Health Sciences, Inc., Hayward, CA 94545, USA

^n^ Quest Diagnostics, Valencia, CA 91355, USA

^o^ Quest Diagnostics, Chantilly, VA 20151, USA

^p^ Roche Diagnostics GmbH, 82377 Penzberg, DE

^q^ Siemens Healthcare Diagnostics Inc., Tarrytown, NY 10591, USA

^r^ Siemens Healthcare Diagnostics Inc., Newark, DE 19702, USA

^s^ Tosoh Corporation, Kanagawa 252-1123, JP

^t^ University College Cork, Cork Centre for Vitamin D and Nutrition Research, Cork T12 YT20, IE

^u^ University of Washington, Department of Laboratory Medicine, Seattle, WA 98185, USA

^v^ Consulting Biostatistician, Shoreview, MN 55126, USA

^w^ National Institutes of Health (NIH), Office of Dietary Supplements (ODS), Bethesda, MD 20817, USA

^x^ National Institute of Standards and Technology (NIST), Chemical Sciences Division, Gaithersburg, MD 20899, USA

**Assay Manufacturers with Multiple Assays in the Study**

Four assay manufacturers provided results using multiple assays for 25(OH)D, i.e., Abbott, IDS, Siemens, and Chromsystems Instruments & Chemicals GmbH. The Abbott Alinity and ARCHITECT assays are based on the same reagents, and the Alinity I instrument is the successor of the ARCHITECT using the same technology. The two IDS assays, IDS-EIA and IDS-iSYS, are based on different assay formats, an electrochemical immunoassay (EIA) and a chemiluminescence immunoassay (CLIA). For the three Siemens assays, the ADVIA Centaur and Atellica IM VitD both use acridinium ester technology with an anti-fluorescein monoclonal antibody covalently bound to paramagnetic particles (PMP), an anti-25(OH) vitamin D monoclonal antibody labeled with acridinium ester (AE), and a vitamin D analog labeled with fluorescein. The Siemens Dimension EXL VITD is based on luminescent oxygen channeling assay (LOCI) technology with a monoclonal antibody directed to total 25(OH)Vitamin D, also anti-3-epimer 25(OH)D_3_ and 25(OH)D_2_ antibodies were added in a Bio-Ab reagent as a blocker or 3-epimer D_3_ interference and 25(OH)D_2_/25(OH)D_3_ equi-molarity. All three Siemens assays use a competitive format where an inverse relationship exists between the amount of 25(OH)D present in the patient sample and the amount of relative light units (RLUs) detected by the system. The Chromsystems Instruments & Chemicals GmbH provided results from their LC-MS/MS assay using two different sample preparation approaches, i.e., reaction vials and 96-well filter plates.

**Discussion of Papers using the IFCC Approach and the Selection of Commutability Criterion (C)**

Korzun et al. [1] assessed commutability of frozen human serum pools using eight assays for the determination of LDL and HDL cholesterol using C values of 4% and 5%, respectively, as recommended by the National Cholesterol Education Program. However, Delatour et al. [2] responded with a letter to the Editor on the Korzun et al. [1] study presenting results for a similar commutability study and suggested “that medical-based criteria are probably too stringent”. Liu et al. [3] reported a commutability study for the determination of hemoglobin A_1c_ in whole blood RMs on six different clinical analyzers using commutability criterion based on the achievable performance of routine methods set at 6%. Zhang et al. [4] assessed the commutability of two commercial EQA materials using eight different commercial thyroid stimulating hormone (TSH) assays using a commutability criterion of 23.7% based on biological variation of TSH. The commutability study reported by Hu et al. [5] assessed RMs and measurement procedures for protein C activity using criterion of 4.5% and 7.5% based on biological variation data. Xing et al. [6] assessed seven reference materials for determination of homocysteine using five different assays with a commutability criterion of 5.15% (50% of the biological variation). A commutability assessment of 15 candidate EQA materials for aminotransferase activity was reported by Long et al. [7] with measurements on seven different assays using C values of 6.7% and 5.6% for alanine aminotransferase and aspartate aminotransferase, respectively, based on biological variation. Yi et al. [8] assessed commutability for 20 processed samples, including human serum pools, charcoal stripped human serum, swine serum (individual and pooled), and commercial EQA samples, for determining progesterone using six immunoassays and found that all samples were assess commutable with the CLSI approach and all were assessed as noncommutable or inconclusive with the IFCC approach using 12.5% as the C value based on “state-of-the-art performance” since no biological variation data were available. Liu et al. [9] assessed urine CRMs for the determination of albumin and creatinine on five clinical analyzers using a C value of 13% and 10%, respectively, based on performance goals established by IFCC and other stakeholder organizations. Deng et al. [10, 11] assessed the commutability of eight processed human plasma samples for normetanephrine and metanephrine using a candidate RMPs and a C value of 15% and serum pools for insulin using a C of 16%, which was 50% of the total allowance error as recommended by the Insulin Standardization Workgroup. In a later study, Deng et al. [12] investigated the suitability of 12 processed EQA materials for the determination of serum C-peptide measurements using a C value of 15% without providing an explanation for this selection. RMs for lipoprotein were evaluated for commutability by Dikaios et al. [13] using MS-based RMP and immunoassays with a commutability criterion of 15%, which represented approximately twice the largest uncertainty associated with difference in bias in their study. Sixteen candidate RMs for plasma renin activity measurement (PRA) were assessed for commutability as reported by Liu et al. [14] using three LC-MS/MS and one immunoassay using a C value of 10%, which was 1/3 of criteria used by the National Center of Clinical Chemistry, NCCL (China). Using biological variation for the bias limits (23.4%), Zhang et al. [15] reported a commutability study carcinoembryonic antigen RMs on five analytical systems. Lyle et al. [16] assessed a WHO RM and human serum pools for six transferrin receptor assays using between biological variability (between subject CV of 19.1%) to set the commutability criterion.

Table S1. Mass spectrometer and LC conditions for the LC-MS/MS assays used in commutability study

| **Participant** | **Assay** | **Mass spectrometer and LC conditions** |
| --- | --- | --- |
| CDC | LC-MS/MS | Gradient elution with 0.35 mL/min of 72% MeOH/DIW increased to 78% MeOH in 8.5 min followed by 100% MeOH for 1.5 min and back to initial conditions for total run time of 13 min; *m/z* transitions monitored: 25(OH)D_3_: 383 → 365,105; IS VitD_3_-*d*_6_: 389 → 371; 25(OH)D_2_: 395 → 377, 209; IS VitD_2_-*d*_3_: 398 → 380; 3-*epi*-25(OH)D_3_: 383 → 365, 105; Internal standards: 3-*epi*-VitD_3_-*d*_3_: 386 → 368; Sample volume required: 100 µL |
| Chromsystems Instruments & Chemicals GmbH | Chromsystems 1  LC‑MS/MS (order no. 62062, sample preparation with reaction vials) | Binary gradient HPLC system with additional isocratic pump for run time of 3.25 min; Analytical Column temperature: 40 °C; Trap Column temperature: 20 °C to 25 °C; Chromsystems Internal Standard Mix (order no. 62045); *m/z* transitions monitored: 25(OH)D_3_: 383 → 257; ISTD 1: 386 → 257; 25(OH)D_2_: 395 → 269; ISTD 3: 398 → 272; 3-*epi*-25(OH)D_3_: 383 → 257; ISTD 2: 389 → 263; Sample volume required:  100 µL |
| Chromsystems Instrument & Chemicals GmbH | Chromsystems 2  LC‑MS/MS (order no. 62062/1000/F, sample preparation with 96 well filter plates) | Binary gradient HPLC system with additional isocratic pump for run time of 3.25 min; Analytical Column temperature: 40 °C; Trap Column temperature: 20 °C to 25 °C; Chromsystems Internal Standard Mix (order no. 62045); *m/z* transitions monitored: 25(OH)D_3_: 383 → 257; ISTD 1: 386 → 257; 25(OH)D_2_: 395 → 269; ISTD 3: 398 → 272; 3-*epi*-25(OH)D_3_: 383 → 257; ISTD 2: 389 → 263; Sample volume required:  100 µL |
| University College Cork | LC‑MS/MS | Mobile Phase A (water) and B (methanol) both containing 2 mmol/L ammonium acetate, 0.1% formic acid; Isocratic separation 29% A, 71% B, 0.45 mL/min; column temperature 35 °C; Internal standards: 25(OH)D_3_-*d*_6_, 3-epi-25(OH)D_3_-*d*_3_, 25(OH)D_2_-*d*_3_ (IsoSciences, King of Prussia, PA, USA); *m/z* transitions monitored: 25(OH)D_3_ and 3-*epi*-25(OH)D_3_ = 401.3 → 159; 25(OH)D_2_ = 413.3 → 355.3; 25(OH)D_3_-*d*_6_ = 407 → 159.1; 3-*epi*-25(OH)D_3_-*d*_3_ = 404.4 → 162; 25(OH)D_2_-*d*_3_ = 416.2 → 358.2; Sample volume required: 150 µL |
| Health Canada | LC‑MS/MS | Mobile phase A (methanol) and B (water) both containing 2.0 mmol/L ammonium acetate and 0.1% formic acid; isocratic elution: 68% A, 32% B; flow rate: 0.4 mL/min; column temperature 30 °C; seal wash and weak needle wash: 90/10 water:MeOH; strong needle wash: 100% MeOH; Internal standards (IS): 25(OH)D_3_-[^13^C_5_], 3-*epi*-25(OH)D_3_-[^13^C_5_], and 25(OH)D_2_-[^13^C_3_] (IsoSciences, >99.0%); *m/z* transitions monitored: 25OHD_3_ = 401.3 → 159, IS 25OHD_3_-[^13^C_5_] = 406.3 → 159; 25(OH)D_2_ = 413.3319 → 355.2631, 413.332 → 395.337, IS 25(OH)D_2_-[^13^C_3_] = 416.4 → 398.3; 3-*epi*-25(OH)D_3_ = 401.3 → 159, IS 3-*epi*-25(OH)D_3_-[^13^C_5_] = 406.3 → 159; Sample volume required: 150 µL |
| Imperial College Healthcare Trust | LC‑MS/MS | Aqueous mobile phase: 2 mmol/L ammonium acetate, 0.1% formic acid in LCMS grade water. Organic mobile phase: 2 mmol/L ammonium acetate, 0.1% formic acid in LCMS grade MeOH; Isocratic elution; Internal standards: 26,26,26,27,27,27-25(OH)D_3_-*d*_6_, 25(OH)D_2_-*d*_3_; *m/z* transitions monitored: 25(OH)D_3_ = 401.25 → 159.10 (quantifier); 25(OH)D_2_ = 413.25 → 83.10 (quantifier); IS 25(OH)D_3_-*d*_6_ = 407.25 → 159.10, IS 25(OH)D_2_-*d*_3_ = 416.25 → 83.10; Sample volume required: 150 µL |
| Quest Diagnostics (Chantilly, VA) | LC-MS/MS | Sample volume required: 100 µL; 0.8 min per sample |
| University of Liège | LC‑MS/MS | Mobile phase A: 0.1% formic acid in water, mobile phase B: 0.1% formic acid in methanol; gradient program: 0.2 min 40% B, 0.3 min 75% B, 5.5 min 75% B, 5.6 min 95% B, 8.0 min 95% B, 8.1 min 40% B; total flow: 0.40 mL/min; column temperature 30 °C; Internal standards: 25(OH)D[_3_-26,26,26,27,27,27](callto:3%20-26,26,26,27,27,27)-*d*_6_ (Medical Isotopes, Inc.), 25(OH)D2-[^2^H_3_] (IsoSciences), 3-*epi*-25(OH)D_3_-*d*_6_ (Toronto Research Chemicals Inc., North York, ON, CA); *m/z* transitions monitored: 25(OH)D_3_ = 401.3 → 365.3; IS 25(OH)D_3_-*d*_6_ = 407.3 → 371.3; 25(OH)D_2_ = 413.5 → 355.5; IS 25(OH)D_2_-*d*_3_ = 416.6 → 358.6; 3-*epi*-25(OH)D_3_ = 401.3 → 365.3; IS 3-*epi*-25(OH)D_3_-*d*_6_ = 407.3 → 371.3; Sample volume required: 200 µL |
| University of Washington | LC‑MS/MS | Isocratic conditions, MPA:B = 18% A:82% B at flow rate 0.4 mL/min; Internal standards: 25(OH)D_2_-26,26,26,27,27,27-*d*_6_, and 25(OH)D_3_-26,26,26,27,27,27-*d*_6_ (Medical Isotopes, Inc., Pelham, NH, USA); *m/z* transitions monitored: 25(OH)D_2_ = 413.40 → 355.35; 25(OH)D_3_ = 401.4 → 365.4; IS 25(OH)D_2_-*d*_6_ = 419.4 → 355.3, and IS 25(OH)D_3_-*d*_6_ = 407.4 → 371.40; Sample volume required: 200 µL |

Table S2. Reactivity and cross reactivity characteristics for ligand binding assays used in this study

| **Assay**  **Manufacturer** | **Assay Kit Name** | **Reactivity/Cross Reactivity** | | | |
| --- | --- | --- | --- | --- | --- |
|  |  | **25(OH)D_2_** | **25(OH)D_3_** | **3-epi-25OH)D_3_** | **24,25(OH)_2_D_3_** |
| Abbott Alinity | Alinity 25-OH Vitamin D | [17] |  |  | [17] |
| Abbott ARCHITECT | Architect 25-OH Vitamin D | 81.5% [18], 63.4 ± 15.6 % [18]; [17]; 76% [19] | 98.6% - 101.1%^a^; 80.5% - 82.4% (LC-MS/MS)^a^ | 0.5% - 1.3%^a^ | 102% - 189%^a^; [17] |
| Affimedix | MicrO-D |  |  |  |  |
| Beckman Coulter | Access 25(OH) Vitamin D Total | 86%^a^ | 100%^a^ | 71%^a^ | -16%^a^; [17] |
| bioMérieux | VIDAS 25 OH Vitamin D Total | 82% [19]; 91% |  |  |  |
| DiaSorin | 25OH Vitamin D Total | 104%^a^; 113 ± 7 % [18]; 110% [19] | 100%^a^ | <1%^a^ | [17] |
| Diazyme | Diazyme EZ Vitamin D Assay | 96.8%^a;^ 106.9% [20] | 100%^a^; 100% [20] | 84.9%^a^; 33% [20] | 23.9%^a^; 118.8% [20] |
| Fujirebio | Lumipulse G 25-OH Vitamin D | 100% [21]; | 100% [21] | 2%^a^; 19.9% [21] | 21%^a^; 5.6% [21] |
| IDS-EIA | 25‑Hydroxy Vitamin D^S^ EIA | 109%^a^ | 95%^a^ | -1%^a^ | 91%^a^ |
| IDS-iSYS | IDS 25VitD^S^ | 120%^a^; 102 ± 1[18]; 100% [18]; 86% [19]; | 97%^a^ | NR^a^ | 124%^a^; [17] |
| PerkinElmer | Total 25OH Vitamin D ELISA |  |  |  |  |
| Roche | Elecsys Vitamin D Total III | 93.7%^a^;105% [21]; 101 ± 6 [18]; 92% 41 ± 76 [22]; 46% [19] | 100%^a^ [21] | 122.4%^a^ [21], minimal [23] | 8.1% [21]; [17]; 13.7%^a^ |
| Siemens ADVIA | Vitamin D Total (VitD) | 104.5%^a^; 104% [18]; 130 ± 31 [18]; 45 ± 54 [22] | 100.7%^a^ | 1.1%^a^ | NR^a^; [17] |
| Siemens Atellica | Vitamin D Total (VitD) |  |  |  |  |
| Siemens Dimension | LOCI Vitamin D Total Assay | 95.1%^a^ | 88.6%^a^ | 2.5%^a^ | 3.9%^a^ |
| Snibe | 25-OH Vitamin D Maglumi | 98.1%^a^ | 96.1%^a^ | 2.0%^a^ | NR^a^ |
| Tosoh | ST AIA-PACK 25-OH Vitamin D | 101%^a^ | 99.2%^a^ | 107%^a^ | 18%^a^ |

^a^ From FDA 510(k) Substantial Equivalence Determination Decision Summary at: <https://www.accessdata.fda.gov/scripts/cdrh/cfdocs/cfpmn/pmn.cfm>; enter the following for specific assays: Abbott ARCHITECT ( k153375), Beckman Coulter (k142362), DiaSorin (k071480), Diazyme (k122420), Fujirebio (k153361), IDS-EIA (k142351), IDS-iSYS (k091849 and k140554), Roche (k162840), Siemens ADVIA (k110586), Siemens Dimension (k162298), Snibe (k191499),Tosoh (k150270 and k123131); NR = not reported in the Decision Summary.

^b^ References provided without a value for cross reactivity contain results indicating reactivity different from equimolar for 25(OH)D2 and 25(OH)D_3_ and cross reactivity for other metabolites.

Table S3. Studies characterizing and comparing various ligand binding assays generally and specifically for pregnant women

| **Assay**  **Manufacturer** | **Assay Kit Name** | **Studies evaluating and comparing assay performance** | **Assay performance studies for pregnant women** |
| --- | --- | --- | --- |
| Abbott Alinity | Alinity 25-OH Vitamin D | [17, 24] |  |
| Abbott ARCHITECT | Architect 25-OH Vitamin D | [17, 25-31] | [25] |
| Affimedix | MicrO-D |  |  |
| Beckman Coulter | Access 25(OH)Vitamin D Total | [17, 24, 29, 31, 32] | [32] |
| bioMérieux | VIDAS 25 OH Vitamin D Total | [31] | [19] |
| DiaSorin | 25OH Vitamin D Total | [17, 25-27, 29, 31-34] | [25, 32, 35, 36] |
| Diazyme | Diazyme EZ Vitamin D Assay | [31] |  |
| Fujirebio | Lumipulse G 25-OH Vitamin D | [21, 31, 33] | [36] |
| IDS-EIA | 25‑Hydroxy Vitamin D^S^ EIA | [31] |  |
| IDS-iSYS | IDS 25VitD^S^ | [17, 25, 26, 31] | [25, 36] |
| PerkinElmer | Total 25OH Vitamin D ELISA |  |  |
| Roche | Elecsys Vitamin D Total III | [17, 21-29, 31-34, 37] | [32, 35, 36] |
| Siemens ADVIA | Vitamin D Total (VitD) | [17, 22, 25-27, 29, 31, 37] | [25, 35] |
| Siemens Atellica | Vitamin D Total (VitD) | [24] |  |
| Siemens Dimension | LOCI Vitamin D Total Assay |  |  |
| Snibe | 25-OH Vitamin D | [31] |  |
| Tosoh | ST AIA-PACK 25-OH Vitamin D |  |  |

Table S4. Measurements used to assign NIST target values to the single-donor samples in this study^a^

| **Sample No.** | **25(OH)D_2_**  **(nmol/L)** | | **25(OH)D_3_**  **(nmol/L)** | | **Total 25(OH)D**  **(nmol/L)** | | |
| --- | --- | --- | --- | --- | --- | --- | --- |
|  | **Replicate 1** | **Replicate 2** | **Replicate 1** | **Replicate 2** | **Replicate 1** | **Replicate 2** | **Mean** |
| 1 | 137.28 | 137.37 | 11.70 | 11.50 | 148.98 | 148.87 | 148.92 |
| 2 | 134.44 | 134.12 | 10.46 | 10.50 | 144.90 | 144.62 | 144.76 |
| 3 | 128.48 | 128.50 | 9.12 | 8.90 | 137.60 | 137.40 | 137.50 |
| 4 | 4.44 | 4.65 | 106.39 | 108.01 | 110.83 | 112.66 | 111.74 |
| 5 | 2.93 | 2.86 | 103.99 | 105.31 | 106.93 | 108.17 | 107.55 |
| 6 | 0.42 | 0.24 | 88.41 | 87.62 | 88.83 | 87.86 | 88.35 |
| 7 | 0.63 | 0.67 | 72.06 | 72.50 | 72.70 | 73.17 | 72.94 |
| 8 | 7.74 | 7.48 | 69.83 | 69.65 | 77.57 | 77.13 | 77.35 |
| 9 | 1.65 | 1.66 | 14.97 | 14.56 | 16.62 | 16.23 | 16.43 |
| 10 | 0.33 | 0.25 | 87.56 | 90.19 | 87.90 | 90.43 | 89.17 |
| 11 | 2.33 | 2.71 | 87.06 | 86.48 | 89.39 | 89.19 | 89.29 |
| 12 | 0.97 | 1.19 | 75.10 | 84.92 | 76.07 | 86.11 | 81.09 |
| 13 | 1.72 | 1.94 | 75.20 | 73.75 | 76.92 | 75.69 | 76.30 |
| 14 | 1.05 | 1.08 | 52.22 | 52.73 | 53.27 | 53.81 | 53.54 |
| 15 | 1.69 | 1.63 | 72.24 | 72.68 | 73.93 | 74.31 | 74.12 |
| 16 | 0.63 | 0.59 | 65.89 | 57.94 | 66.52 | 58.52 | 62.52 |
| 17 | 1.32 | 1.31 | 32.44 | 32.89 | 33.76 | 34.19 | 33.98 |
| 18 | 0.94 | 1.13 | 43.09 | 41.88 | 44.03 | 43.02 | 43.52 |
| 19 | 1.41 | 1.40 | 123.81 | 124.20 | 125.22 | 125.60 | 125.41 |
| 20 | 2.25 | 2.30 | 61.72 | 62.37 | 63.97 | 64.67 | 64.32 |
| 21 | 1.44 | 1.38 | 78.56 | 81.15 | 80.00 | 82.53 | 81.26 |
| 22 | 0.39 | 0.41 | 30.30 | 30.07 | 30.70 | 30.48 | 30.59 |
| 23 | 31.95 | 32.43 | 9.84 | 9.89 | 41.79 | 42.31 | 42.05 |
| 24 | 1.22 | 1.22 | 27.70 | 27.91 | 28.93 | 29.13 | 29.03 |
| 25 | 3.38 | 3.43 | 71.26 | 71.64 | 74.65 | 75.07 | 74.86 |

Table S4. Measurements used to assign NIST target values to the single-donor samples in this study^a^ (continued)

| **Sample No.** | **25(OH)D_2_**  **(nmol/L)** | | **25(OH)D_3_**  **(nmol/L)** | | | **Total 25(OH)D**  **(nmol/L)** | | |
| --- | --- | --- | --- | --- | --- | --- | --- | --- |
|  | **Replicate 1** | **Replicate 2** | **Replicate 1** | **Replicate 2** | **Replicate 1** | | **Replicate 2** | **Mean** |
| 26 | 0.64 | 0.64 | 30.09 | 28.72 | 30.73 | | 29.36 | 30.04 |
| 27 | 0.46 | 0.47 | 84.54 | 83.85 | 85.00 | | 84.32 | 84.66 |
| 28 | 1.24 | 1.30 | 51.65 | 51.02 | 52.89 | | 52.32 | 52.61 |
| 29 | 1.70 | 1.70 | 48.85 | 48.69 | 50.55 | | 50.39 | 50.47 |
| 30 | 0.36 | 0.35 | 60.81 | 61.34 | 61.17 | | 61.68 | 61.43 |
| 31 | 68.05 | 61.09 | 15.51 | 14.07 | 83.56 | | 75.16 | 79.36 |
| 32 | 0.36 | 0.31 | 22.17 | 19.81 | 22.53 | | 20.12 | 21.32 |
| 33 | 2.99 | 2.84 | 59.57 | 60.77 | 62.56 | | 63.61 | 63.09 |
| 34 | 86.89 | 87.72 | 10.40 | 9.96 | 97.28 | | 97.67 | 97.48 |
| 35 | 0.64 | 0.74 | 40.99 | 39.85 | 41.64 | | 40.59 | 41.11 |
| 36 | 1.89 | 2.12 | 66.36 | 64.57 | 68.24 | | 66.69 | 67.47 |
| 37 | 0.43 | 0.36 | 99.61 | 98.91 | 100.04 | | 99.26 | 99.65 |
| 38 | 0.44 | 0.50 | 128.65 | 129.06 | 129.09 | | 129.56 | 129.33 |
| 39 | 99.46 | 99.32 | 12.65 | 12.41 | 112.11 | | 111.73 | 111.92 |
| 40 | 0.48 | 0.50 | 59.31 | 56.92 | 59.79 | | 57.42 | 58.61 |
| 41 | 0.93 | 0.96 | 64.11 | 65.13 | 65.04 | | 66.09 | 65.57 |
| 42 | 1.06 | 1.03 | 49.38 | 49.61 | 50.44 | | 50.63 | 50.54 |
| 43 | 1.61 | 1.63 | 44.01 | 44.45 | 45.62 | | 46.08 | 45.85 |
| 44 | 1.21 | 1.19 | 141.72 | 142.57 | 142.93 | | 143.76 | 143.35 |
| 45 | 1.29 | 1.40 | 56.99 | 56.10 | 58.28 | | 57.50 | 57.89 |
| 46 | 0.50 | 0.51 | 61.16 | 58.36 | 61.66 | | 58.86 | 60.26 |
| 47 | 0.43 | 0.41 | 132.70 | 135.64 | 133.13 | | 136.05 | 134.59 |
| 48 | 0.55 | 0.67 | 37.93 | 37.27 | 38.48 | | 37.94 | 38.21 |
| 49 | 47.37 | 47.04 | 89.90 | 92.46 | 137.28 | | 139.50 | 138.39 |
| 50 | 2.11 | 2.13 | 117.60 | 118.99 | 119.71 | | 121.12 | 120.42 |

^a^ These measurements were originally reported as the mean of duplicate injections of two replicates [38]; these results represent the first injection for each replicate.

Table S5. Measurements used for assigning NIST certified values to SRM 2969, SRM 2970, SRM 2973, and SRM 972a L1

| **SRM No.** | **Position** | **25(OH)D_2_**  **(nmol/L)** | | **25(OH)D_3_**  **(nmol/L)** | | **Total 25(OH)D**  **(nmol/L)** | | |
| --- | --- | --- | --- | --- | --- | --- | --- | --- |
|  |  | **Replicate 1** | **Replicate 2** | **Replicate 1** | **Replicate 2** | **Replicate 1** | **Replicate 2** | **Mean** |
| 2969 | 1 | 4.90 | 4.82 | 29.61 | 30.22 | 34.51 | 35.05 | 34.78 |
| 2969 | 2 | 4.84 | 4.90 | 29.58 | 29.99 | 34.42 | 34.89 | 34.66 |
| 2969 | 3 | 4.87 | 4.93 | 29.79 | 30.17 | 34.66 | 35.10 | 34.88 |
| 2969 | 4 | 4.88 | 4.92 | 30.07 | 29.92 | 34.95 | 34.84 | 34.90 |
| 2969 | 5 | 4.82 | 4.85 | 29.43 | 29.40 | 34.25 | 34.26 | 34.26 |
|  |  |  |  |  |  |  |  |  |
| 2970 | 1 | 57.79 | 56.77 | 23.74 | 24.31 | 81.53 | 81.08 | 81.31 |
| 2970 | 2 | 56.84 | 56.89 | 24.11 | 24.07 | 80.95 | 80.96 | 80.96 |
| 2970 | 3 | 56.46 | 56.74 | 24.19 | 23.68 | 80.66 | 80.42 | 80.54 |
| 2970 | 4 | 57.91 | 56.50 | 23.78 | 23.73 | 81.69 | 80.23 | 80.96 |
| 2970 | 5 | 56.76 | 57.35 | 23.60 | 24.17 | 80.36 | 81.52 | 80.95 |
|  |  |  |  |  |  |  |  |  |
| 2973 | 1 | 1.63 | 1.59 | 98.24 | 97.98 | 99.87 | 99.57 | 99.72 |
| 2973 | 2 | 1.51 | 1.54 | 97.98 | 99.00 | 99.49 | 100.54 | 100.02 |
| 2973 | 3 | 1.64 | 1.54 | 99.00 | 99.51 | 100.64 | 101.05 | 100.85 |
| 2973 | 4 | 1.66 | 1.68 | 99.00 | 98.49 | 100.66 | 100.18 | 100.42 |
| 2973 | 5 | 1.56 | 1.54 | 97.22 | 98.23 | 98.78 | 99.77 | 99.28 |
|  |  |  |  |  |  |  |  |  |
| 972a L1 | 1 | 1.22 | 1.19 | 73.71 | 73.53 | 74.93 | 74.72 | 74.83 |
| 972a L1 | 2 | 1.19 | 1.26 | 73.66 | 73.28 | 74.85 | 74.54 | 74.70 |
| 972a L1 | 3 | 1.17 | 1.14 | 74.32 | 73.89 | 75.49 | 75.03 | 75.26 |
| 972a L1 | 4 | 1.14 | 1.12 | 73.58 | 73.78 | 74.72 | 74.90 | 74.81 |
| 972a L1 | 5 | 1.09 | 1.24 | 74.07 | 73.94 | 75.16 | 75.18 | 75.17 |

^a^ These measurements were originally reported as the mean of duplicate injections of two replicates[39-41]; these results represent the first injection for each replicate.

Table S6. Measurements used for assigning NIST reference and certified values to SRM 1949 and SRM 972a L2

| **SRM No.** | **Position** | **25(OH)D_2_**  **(nmol/L)** | | | **25(OH)D_3_**  **(nmol/L)** | | | **Total 25(OH)D**  **(nmol/L)** | | |
| --- | --- | --- | --- | --- | --- | --- | --- | --- | --- | --- |
|  |  | **Replicate 1** | **Replicate 2** | **Replicate 1** | | **Replicate 2** | **Replicate 1** | | **Replicate 2** | **Mean** |
| 972a L2 | 1 | 1.91 | 1.86 | 45.77 | | 45.90 | 47.68 | | 47.76 | 47.72 |
| 972a L2 | 2 | 1.88 | 1.86 | 45.16 | | 45.03 | 47.04 | | 46.89 | 46.97 |
| 972a L2 | 3 | 1.91 | 1.89 | 44.82 | | 44.95 | 46.73 | | 46.84 | 46.79 |
| 972a L2 | 4 | 1.91 | 1.91 | 45.10 | | 46.07 | 47.01 | | 47.98 | 47.50 |
| 972a L2 | 5 | 1.86 | 1.88 | 45.49 | | 45.36 | 47.35 | | 47.24 | 47.30 |
|  |  |  |  |  | |  |  | |  |  |
| 1949 NP | 1 | 0.734 | 0.660 | 63.62 | | 61.36 | 64.35 | | 62.02 | 63.19 |
| 1949 NP | 2 | 0.702 | 0.689 | 62.22 | | 61.81 | 62.92 | | 62.49 | 62.71 |
| 1949 NP | 3 | 0.697 | 0.771 | 61.62 | | 61.63 | 62.31 | | 62.40 | 62.36 |
|  |  |  |  |  | |  |  | |  |  |
| 1949 T1 | 1 | 2.79 | 2.66 | 63.66 | | 66.06 | 66.45 | | 68.72 | 67.59 |
| 1949 T1 | 2 | 3.04 | 2.92 | 64.41 | | 64.80 | 67.45 | | 67.72 | 67.59 |
| 1949 T1 | 3 | 2.94 | 3.01 | 64.77 | | 66.09 | 67.70 | | 69.10 | 68.40 |
|  |  |  |  |  | |  |  | |  |  |
| 1949 T2 | 1 | 1.23 | 1.43 | 74.39 | | 72.28 | 75.62 | | 73.71 | 74.67 |
| 1949 T2 | 2 | 1.23 | 1.43 | 75.97 | | 75.44 | 77.20 | | 76.87 | 77.04 |
| 1949 T2 | 3 | 1.25 | 1.16 | 75.54 | | 73.57 | 76.79 | | 74.73 | 75.76 |
|  |  |  |  |  | |  |  | |  |  |
| 1949 T3 | 1 | 2.31 | 1.89 | 74.91 | | 73.08 | 77.22 | | 74.98 | 76.10 |
| 1949 T3 | 2 | 2.49 | 1.96 | 72.40 | | 73.23 | 74.89 | | 75.19 | 75.04 |
| 1949 T3 | 3 | 2.35 | 2.23 | 74.75 | | 73.61 | 77.10 | | 75.84 | 76.47 |

^a^ These measurements for SRM 972a L2 were originally reported as the mean of duplicate injections of two replicates[40]; these results represent the first injection for each replicate. Results for SRM 1949 also represent the first injection for each of two replicates and have not been published previously.

Table S7. Commutability studies using the IFCC guidelines and the basis for selection of commutability criterion (C)

| **Authors/Reference** | **Measurand(s)** | **Commutability Criterion (C)** | **C Selection Based on:** | **Samples Assessed/No. of Assays** |
| --- | --- | --- | --- | --- |
| Korzun, Nilsson, Bachmann, Myers, Sakurabayashi et al. [1] | LDL and HDL cholesterol | 4% (LDL) and 5% (HDL) | Recommendations of the National Cholesterol Education Program | frozen human serum pools using 8 assays |
| Delouter, Liu, and Vesper [2] | LDL and HDL cholesterol | 10% to 11% | Random error components; authors state that “medical based criteria” may be too stringent | 5 frozen serum pools analyzed on 8 clinical analyzers |
| Liu, Wong, Yong, Liu, Teo, Lee, Loh, Sethi et al. [3] | hemoglobin A_1c_ | 6% | Achievable performance of routine methods | whole blood RMs using 6 different clinical analyzers |
| Zhang, Chang, Wang, Wen, Zeng et al. [4] | thyroid stimulating hormone (TSH) | 23.7% | Biological variation | 2 commercial EQA materials using 8 different TSH assays |
| Hu, Sun, Yu, Li, Liu, and Peng [5] | protein C (PC) activity | 4.5% if PC activity <60% and 7.5% if > 60% | Biological variation | 6 RMs and MPs |
| Xing, Liu, Sun, Gao, Ju, Liu, and Song [6] | homocysteine | 5.15% | Half of biological variation | 7 RMs using 5 different assays |
| Long, Qi, Zhang, Wang, Zeng, Yan, Wang, Huang, Zhao, Chen, and Zhang [7] | alanine aminotransferase and aspartate aminotransferase | 6.7% (alanine) and 5.6% (aspartate) | Biological variation | 15 candidate EQA materials |
| Yi, Wang, Zhang, Zeng, Zhao, Zhou, Zhang, Yan, Chen, and Zhang [8] | progesterone | 12.5% | State-of the-art performance | 20 processed samples including human serum pools, charcoal stripped human serum, swine serum, and commercial EQAs using 6 immunoassays |
| Liu, Ng, Liu, Teo, Loh, Wang, Sethi, Tan, Heng, Saw et al. [9] | albumin and creatinine | 13% (albumin) and 10% (creatinine) | Performance goals established by IFCC and stakeholders | 2 urine CRMs on 5 different clinical analyzers |
| Deng, Liu, Liu, Zhao, and Zhou [10] | Normetanephrine and metanephrine | 15% | 50% total allowance error as recommended by NCCL (China) | 8 processed human plasma samples |
| Deng, Zhang, Li, Wang, Zeng, Zhang, Zhang, Zhao, Zhou, and Zhang [12] | C-peptide | 15% | No explanation | 12 processed samples analyzed using LC-MS/MS and 6 immunoassays |
| Deng, Zhang, Wang, Zeng, Zhang, Zhang, Zhao, Li, Zhao, Gan, Shao et al. [11] | insulin | 16% | 50% total allowance error as recommended by Insulin Standardization Workgroup | 10 serum pools and 3 EQA samples analyzed by 6 immunoassays |
| Dikaios, Althaus, Angles-Cano, Ceglarek, Coassin, et al. [13] | lipoprotein (a) | 15% | 2x the largest uncertainty associated with the difference in bias in their study | 14 RMs for lipoproteins using 8 immunoassay-based MPs |
| Liu, Jin, Ma, Nizhamuding, Zeng, Zhang, Zhang, Zhou, and Zhang [14] | plasma renin activity (PRA) | 10% | 1/3 of criteria used by the National Center for Clinical Chemistry (NCCL, China) | 16 RMs |
| Zhang, Xu, Zhao, Fu, Song, Wang, and Yue [15] | carcinoembryonic antigen | 23.4% | Biological variation | carcinoembryonic antigen RMs using 5 analytical systems |
| Lyle, Budd, Kennerley, Smith, Danilenko et al. [16] | transferrin receptor | 19.1% | Biological variation (between person) | WHO 07/202 sTfR RM and human serum pools |

Table S8. Ordinary Deming regression analysis for ligand binding and LC‑MS/MS assays for commutability assessment using CLSI Approach with 95% PI

| **Assays** | **50 Sample Set** | | | | | | **42 Sample Set^a^** | | | | | | **Difference 50 – 42 Sample Sets^d^** | | |
| --- | --- | --- | --- | --- | --- | --- | --- | --- | --- | --- | --- | --- | --- | --- | --- |
|  | **Regression Line^b^** | | | **95% PI^c^ (nmol/L)** | | | **Regression Line^b^** | | | **95% PI^c^ (nmol/L)** | | |  |  |  |
|  | **Slope** | **Int.^b^** | **R^2^** | **Min** | **Max** | **Width** | **Slope** | **Int.^b^** | **R^2^** | **Min** | **Max** | **Width** | **Slope** | **Width** | **R^2^** |
| **Ligand Binding Assays** |  | | | | | | | | | | | | | | |
| Abbott (Alinity) | 0.839 | 7.90 | 0.726 | ‑29.4 | 45.2 | 74.6 | 1.119 | ‑6.09 | 0.956 | ‑21.3 | 9.2 | 30.5 | -0.280 | 44.1 | -0.230 |
| Abbott (Alinity) (U of Liège) | 0.872 | 7.1 | 0.717 | -32.1 | 46.4 | 78.5 | 1.165 | -7.4 | 0.956 | -23.1 | 8.2 | 31.3 | -0.293 | 47.1 | -0.239 |
| Abbott (Alinity) ICHT) | 0.825 | 10.3 | 0.721 | -26.6 | 47.3 | 73.9 | 1.102 | -3.4 | 0.958 | -17.8 | 10.9 | 28.7 | -0.274 | 45.2 | -0.237 |
| Abbott (ARCHITECT) | 0.900 | 6.58 | 0.711 | -34.3 | 47.4 | 81.7 | 1.205 | -8.56 | 0.949 | -26.0 | 8.9 | 34.9 | -0.305 | 46.8 | -0.238 |
| Affimedix | 1.198 | -10.4 | 0.933 | -32.5 | 11.6 | 44.1 | 1.189 | -10.6 | 0.916 | -33.1 | 12.0 | 45.0 | 0.009 | -0.9 | 0.017 |
| Beckman Coulter (U of Liège) | 1.222 | ‑14.3 | 0.904 | ‑42.3 | 13.8 | 56.1 | 1.206 | ‑13.2 | 0.865 | ‑45.1 | 18.8 | 63.9 | 0.016 | -7.8 | 0.039 |
| Biomérieux | 0.945 | 3.08 | 0.638 | ‑46.3 | 52.4 | 98.7 | 1.264 | ‑13.0 | 0.878 | ‑42.3 | 16.3 | 58.6 | -0.319 | 40.1 | -0.240 |
| Biomérieux(U of Liège) | 0.964 | 3.8 | 0.638 | ‑46.4 | 53.0 | 98.4 | 1.293 | ‑12.8 | 0.884 | ‑42.2 | 16.6 | 58.8 | -0.329 | 39.6 | -0.246 |
| DiaSorin (U of Liège) | 1.050 | -0.3 | 0.861 | -30.1 | 29.5 | 59.6 | 1.198 | -7.7 | 0.904 | -32.7 | 17.3 | 50.0 | -0.148 | 9.6 | -0.043 |
| DiaSorin (ICHT) | 1.103 | 2.6 | 0.859 | -28.9 | 34.0 | 62.9 | 1.264 | -5.5 | 0.912 | -30.4 | 19.4 | 49.8 | -0.161 | 13.1 | -0.053 |
| Diazyme | 1.024 | -2.8 | 0.848 | -35.8 | 28.1 | 63.9 | 1.140 | -9.1 | 0.859 | -38.2 | 20.0 | 58.2 | -0.116 | 5.7 | -0.011 |
| Fujirebio | 1.111 | -3.5 | 0.990 | -11.5 | 4.6 | 16.1 | 1.111 | -3.6 | 0.988 | -11.4 | 4.2 | 15.6 | 0.000 | 0.5 | 0.002 |
| Fujirebio (U of Liège) | 1.069 | -3.4 | 0.988 | -11.6 | 4.7 | 16.3 | 1.066 | -3.3 | 0.986 | -11.5 | 4.8 | 16.3 | 0.003 | 0.00 | 0.002 |
| IDS‑EIA | 0.899 | 16.0 | 0.689 | -26.6 | 58.5 | 84.5 | 1.106 | 5.0 | 0.767 | -32.2 | 42.2 | 74.4 | -0.207 | 10.1 | -0.078 |
| IDS‑iSYS | 0.896 | 5.3 | 0.904 | ‑15.8 | 26.4 | 42.2 | 0.983 | 0.7 | 0.918 | -18.4 | 19.8 | 38.2 | -0.087 | 4.0 | -0.014 |
| IDS-ISYS (U of Liège) | 0.947 | 3.7 | 0.927 | -15.3 | 22.7 | 38.0 | 1.010 | 0.6 | 0.927 | -17.6 | 18.8 | 36.4 | -0.063 | 1.6 | 0.000 |
| PerkinElmer | 1.025 | -6.3 | 0.927 | -26.6 | 14.0 | 40.6 | 0.985 | -4.4 | 0.902 | -25.0 | 16.2 | 41.2 | 0.040 | -0.6 | 0.025 |
| Roche | 1.109 | -7.1 | 0.958 | -23.5 | 9.2 | 32.7 | 1.081 | -5.4 | 0.944 | -22.4 | 11.5 | 33.9 | 0.028 | -1.2 | 0.014 |
| Roche (U of Liège) | 1.141 | -8.1 | 0.947 | -27.3 | 11.1 | 38.4 | 1.139 | -8.0 | 0.925 | -28.8 | 12.9 | 41.7 | 0.002 | -3.3 | 0.022 |
| Siemens ADVIA Centaur XP | 1.369 | -15.2 | 0.891 | -49.2 | 18.8 | 68.0 | 1.162 | -3.6 | 0.891 | -30.6 | 23.3 | 53.8 | 0.207 | 14.2 | 0.000 |
| Siemens ADVIA Centaur XP (U of Liège) | 1.424 | -18.1 | 0.854 | -59.2 | 23.0 | 82.2 | 1.157 | -3.2 | 0.848 | -35.1 | 28.6 | 63.7 | 0.267 | 18.5 | 0.006 |
| Siemens Atellica IM | 1.309 | -14.5 | 0.880 | -48.5 | 19.5 | 68.0 | 1.077 | -2.0 | 0.878 | -28.3 | 24.4 | 52.7 | 0.232 | 15.3 | -0.002 |
| Siemens Dimension XL | 1.177 | -6.1 | 0.897 | -34.2 | 22.1 | 56.3 | 1.202 | -7.2 | 0.869 | -37.9 | 23.4 | 61.3 | -0.025 | -5.0 | 0.028 |
| Snibe (U of Liège) | 1.012 | 8.5 | 0.908 | -14.5 | 31.5 | 46.0 | 1.161 | 0.9 | 0.951 | -15.5 | 17.3 | 32.8 | -0.149 | 13.2 | -0.043 |
| Tosoh | 1.310 | -17.9 | 0.828 | -60.0 | 24.2 | 84.2 | 1.391 | -22.2 | 0.783 | -70.9 | 26.4 | 97.3 | 0.081 | -13.1 | -0.045 |
| **LC-MS/MS Assays** |  | | | | | | | | | | | | | | |
| CDC | 0.998 | 3.3 | 0.986 | -5.3 | 11.8 | 17.1 | 1.028 | 1.6 | 0.984 | -6.5 | 9.8 | 16.3 | -0.030 | 0.8 | 0.002 |
| Chromsystems 1 | 1.094 | -0.4 | 0.984 | -9.9 | 9.2 | 19.1 | 1.078 | 0.4 | 0.980 | -9.1 | 9.8 | 18.9 | 0.016 | 0.2 | 0.004 |
| Chromsystems 2 | 0.992 | 2.2 | 0.962 | -11.6 | 15.9 | 27.5 | 1.059 | -1.6 | 0.978 | -11.6 | 8.4 | 20.0 | -0.067 | 7.5 | -0.016 |
| Cork | 1.031 | 0.6 | 0.980 | -10.1 | 11.2 | 21.3 | 1.040 | -0.3 | 0.980 | -10.0 | 9.3 | 19.3 | -0.009 | 2.0 | 0.000 |
| Health Canada | 1.085 | -1.6 | 0.986 | -10.4 | 7.2 | 17.6 | 1.087 | -1.6 | 0.986 | -9.6 | 6.4 | 16.0 | 0.002 | 1.6 | 0.000 |
| ICHT | 1.058 | 1.6 | 0.978 | -9.6 | 12.9 | 22.5 | 1.133 | -2.4 | 0.986 | -10.6 | 5.8 | 16.4 | 0.075 | 6.1 | 0.008 |
| Quest Diagnostics (VA) | 1.046 | -4.5 | 0.968 | -18.2 | 9.3 | 27.5 | 0.949 | 0.9 | 0.978 | -8.3 | 10.2 | 18.5 | 0.097 | 9.0 | -0.010 |
| U of Liège | 1.018 | -0.4 | 0.986 | -9.0 | 8.3 | 17.3 | 1.008 | -0.1 | 0.984 | -8.3 | 8.1 | 16.4 | 0.010 | 0.9 | 0.002 |
| U of Washington | 0.991 | -1.6 | 0.988 | -9.5 | 6.4 | 15.9 | 0.972 | -0.6 | 0.984 | -8.7 | 7.5 | 16.2 | 0.019 | -0.3 | 0.004 |

^a^ The 42 single-donor sample set excludes eight samples from the 50 single-donor sample set with elevated concentrations of 25(OH)D_2_ i.e., > 30 nmol/L.

^b^ For the regression line, Int. = y‑intercept in nmol/L

^c^ Min = minimum y‑intercept and Max = maximum y‑intercept in nmol/L; width = Min + Max values in nmol/L

^d^ Difference in the values for the 50-sample set minus the 42-sample set for slope, R^2^, and width of PI

Table S9. Commutability of SRMs for LC-MS/MS assays using CLSI 95% PI approach (50 or 42 samples)

|  | **SRMs^a^** | | | | | | | |
| --- | --- | --- | --- | --- | --- | --- | --- | --- |
| **LC-MS/MS Assay^b^** | **2969** | **1949 NP** | **1949 T1** | **1949 T2** | **1949 T3** | **2970** | **972a L1** | **2973** |
| Centers for Disease Control and Prevention (CDC) | C | C | C | C | C | C |  |  |
| Chromsystems 1 | C | C | C | C | C | C |  |  |
| University College Cork | C | C | C | C | C | C |  |  |
| University of Washington | C | C | C | C | C | C |  |  |
| Health Canada | C | C | C | C | C | C |  |  |
| Chromsystems 2 | C | C | C | C | C | C |  |  |
| Imperial College Healthcare Trust (ICHT) | C | C | C | C | C | C |  |  |
| Quest Diagnostics (Chantilly, VA) | C | C | C | C | C | C |  |  |
| University of Liège | C |  |  |  |  | C | C | C |

^a^ C = commutable (green). Blank cells in table indicate that the sample was not analyzed.

^b^ Assays ordered as in Table 5.

Table S10. Commutability of SRMs for ligand binding assays using CLSI 95% PI approach (50 samples)

| **Assay^b^** | **SRM^a^** | | | | | | | |
| --- | --- | --- | --- | --- | --- | --- | --- | --- |
|  | **2969** | **1949 NP** | **1949 T1** | **1949 T2** | **1949 T3** | **2970** | **972a L1** | **2973** |
| Roche | C | C | C | C | C | C |  |  |
| Roche (U of Liège) | C | C | C | C | C | C |  |  |
| Tosoh | C | C | C | C | C | C | C | C |
| Abbott Alinity | C | C | C | C | C | C |  |  |
| Abbott Alinity (U of Liège) | C | C | C | C | C | C |  |  |
| Abbott Alinity (ICHT) | C | C | C | C | C | C |  |  |
| Abbott ARCHITECT | C | C | C | C | C | C |  |  |
| DiaSorin (U of Liège) | C | C | C | C | C | C |  |  |
| DiaSorin (ICHT) | C | C | C | C | C | C |  |  |
| IDS iSYS | C | C | C | C | C | C |  |  |
| IDS iSYS (U of Liège) | C | C | C | C | C | C |  |  |
| bioMérieux | C | C | C | C | C | C |  |  |
| bioMérieux (U of Liège) | C | C | C | C | C | C |  |  |
| Siemens Dimension | C | C | C | C | C | C |  |  |
| IDS-EIA | C | C | C | C | C | C |  |  |
| Affimedix | C | C | C | C | C | C | C | C |
| Beckman Coulter (U of Liège) | C | C | C | C | C | C |  |  |
| Fujirebio | C | C | C | NC | NC | C | C | C |
| Fujirebio (U of Liège) | C | C | C | NC | NC | C |  |  |
| PerkinElmer | C | C | C | C | NC | C | C | C |
| Affimedix | C | C | C | C | C | C | C | C |
| Diazyme | C | C | C | C | C | C | C | C |
| Siemens ADVIA Centaur XP | C | C | C | C | NC | C |  |  |
| Siemens ADVIA Centaur XP (U of Liège) | C | C | C | C | C | C |  |  |
| Siemens Atellica IM | C | C | C | C | C | C |  |  |
| Snibe (U of Liège) | C | C | C | C | C | C |  |  |

^a^ C = commutable (green) and NC = noncommutable (yellow). Blank cells indicate that the sample was not analyzed.

^b^ Assays ordered as in Table 6.

Table S11. Commutability of SRMs for ligand binding assays using CLSI 95% PI approach (42 samples)

| **Assay^b^** | **SRM^a^** | | | | | | | |
| --- | --- | --- | --- | --- | --- | --- | --- | --- |
|  | **2969** | **1949NP** | **1949T1** | **1949T2** | **1949T3** | **2970** | **972aL1** | **2973** |
| Roche | C | C | C | C | C | C |  |  |
| Roche (U of Liège) | C | C | C | C | C | C |  |  |
| Tosoh | C | C | C | C | C | C | C | C |
| Abbott Alinity | C | C | C | C | C | NC |  |  |
| Abbott Alinity (U of Liège) | C | C | C | C | C | NC |  |  |
| Abbott Alinity (ICHT) | C | C | C | C | C | NC |  |  |
| Abbott ARCHITECT | C | C | C | C | C | NC |  |  |
| DiaSorin (U of Liège) | C | C | C | C | C | NC |  |  |
| DiaSorin (ICHT) | C | C | C | C | C | NC |  |  |
| IDS iSYS | C | C | C | C | C | C |  |  |
| IDS iSYS (U of Liège) | C | C | C | C | C | C |  |  |
| bioMérieux | C | C | C | C | C | NC |  |  |
| bioMérieux (U of Liège) | C | C | C | C | C | NC |  |  |
| Siemens Dimension | C | C | C | C | C | C |  |  |
| IDS-EIA | C | C | C | C | C | NC |  |  |
| Beckman Coulter (U of Liège) | C | C | C | C | C | C |  |  |
| Fujirebio | C | C | C | NC | NC | C | C | C |
| Fujirebio (U of Liège) | C | C | C | NC | NC | C |  |  |
| PerkinElmer | C | C | C | C | NC | C | C | C |
| Affimedix | C | C | C | C | C | C | C | C |
| Diazyme | C | C | C | C | C | C | C | C |
| Siemens ADVIA Centaur XP | C | C | C | C | NC | C |  |  |
| Siemens ADVIA Centaur XP (U of Liège) | C | C | C | C | C | C |  |  |
| Siemens Atellica | C | C | C | C | NC | C |  |  |
| Snibe (U of Liège) | C | C | C | C | NC | NC |  |  |

^a^ C = commutable (green) and NC = noncommutable (yellow). Blank cells indicate that the sample was not analyzed.

^b^ Assays ordered as in Table 6.

Table S12. Ordinary Deming regression analysis for ligand binding and LC‑MS/MS assays for commutability assessment using CLSI Approach with 95% PI using a 38-sample set with 25(OH)D < 100 nmol/L

| **Assays** | **38 Sample Set 25(OH)D < 100 nmol/L^a^** | | | | | | **Difference 50 – 38 Sample Sets^d^** | | | **Difference 42– 38 Sample Sets^d^** | | |
| --- | --- | --- | --- | --- | --- | --- | --- | --- | --- | --- | --- | --- |
|  | **Regression Line^b^** | | | **95% PI^c^ (nmol/L)** | | |  |  |  |  |  |  |
|  | **Slope** | **Int.^b^** | **R^2^** | **Min** | **Max** | **Width** | **Slope** | **Width** | **R^2^** | **Slope** | **Width** | **R^2^** |
| **Ligand Binding Assays** |  | | | | | | | | | | | |
| Abbott (Alinity) | 1.080 | -5.3 | 0.815 | ‑29.7 | 19.6 | 49.3 | -0.241 | 25.3 | -0.089 | 0.039 | -18.8 | 0.141 |
| Abbott (Alinity) (Liège) | 1.122 | -6.5 | 0.823 | -30.8 | 17.9 | 48.7 | -0.250 | 29.8 | -0.106 | 0.043 | -17.4 | 0.133 |
| Abbott (Alinity) ICHT) | 1.108 | -5.3 | 0.897 | -29.6 | 19.1 | 48.7 | -0.283 | 25.2 | -0.176 | -0.006 | -20 | 0.061 |
| Abbott (ARCHITECT) | 1.186 | -9.0 | 0.805 | -36.1 | 18.1 | 54.2 | -0.286 | 27.5 | -0.094 | 0.019 | -19.3 | 0.144 |
| Affimedix | 1.284 | -15.0 | 0.885 | -35.1 | 5.0 | 40.1 | -0.086 | 4.0 | 0.048 | -0.095 | 4.9 | 0.031 |
| Beckman Coulter (Liège) | 1.089 | ‑6.0 | 0.887 | ‑24.2 | 12.2 | 36.4 | 0.133 | 19.7 | 0.017 | 0.117 | 27.5 | -0.022 |
| Biomérieux | 1.261 | 14.4 | 0.712 | ‑49.8 | 21.0 | 70.8 | -0.316 | 27.9 | -0.074 | 0.003 | -12.2 | 0.166 |
| Biomérieux (Liège) | 1.280 | -13.7 | 0.722 | ‑48.8 | 21.4 | 70.2 | -0.316 | 28.2 | -0.084 | 0.013 | -11.4 | 0.162 |
| DiaSorin (Liège) | 1.173 | -6.8 | 0.885 | -25.9 | 12.2 | 38.1 | -0.123 | 21.5 | -0.024 | 0.025 | 11.9 | 0.019 |
| DiaSorin (ICHT) | 1.190 | -1.8 | 0.885 | -20.9 | 17.3 | 38.2 | -0.087 | 24.7 | -0.026 | 0.074 | 11.6 | 0.027 |
| Diazyme | 1.258 | -15.3 | 0.815 | -41.4 | 10.8 | 52.2 | -0.234 | 11.7 | 0.033 | -0.118 | 6.0 | 0.044 |
| Fujirebio | 1.169 | -6.4 | 0.978 | -14.2 | 1.3 | 17.4 | -0.058 | -1.3 | 0.012 | -0.058 | -1.8 | 0.01 |
| Fujirebio (Liège) | 1.135 | -6.8 | 0.982 | -13.6 | 0.0 | 13.6 | -0.066 | 2.7 | 0.006 | -0.069 | 2.7 | 0.004 |
| IDS‑EIA | 1.303 | -6.0 | 0.740 | -38.9 | 26.8 | 65.7 | -0.404 | 18.8 | -0.051 | -0.197 | 8.7 | 0.027 |
| IDS‑iSYS | 1.062 | -3.4 | 0.920 | ‑18.3 | 11.5 | 30.0 | -0.166 | 12.2 | -0.016 | -0.079 | 8.2 | -0.002 |
| IDS-ISYS (Liège) | 1.108 | -4.8 | 0.931 | -18.6 | 9.1 | 27.7 | -0.161 | 10.3 | -0.004 | -0.098 | 8.7 | -0.004 |
| PerkinElmer | 0.977 | -3.4 | 0.863 | -20.2 | 13.4 | 33.6 | 0.048 | 77.0 | 0.064 | 0.008 | 7.6 | 0.039 |
| Roche | 1.108 | -6.5 | 0.937 | -20.1 | 7.1 | 27.2 | 0.001 | 5.5 | 0.021 | -0.027 | 6.7 | 0.007 |
| Roche (Liège) | 1.190 | -10.3 | 0.891 | -30.3 | 9.7 | 40.0 | -0.049 | -1.6 | 0.056 | -0.051 | 1.7 | 0.034 |
| Siemens ADVIA Centaur XP | 1.156 | -1.9 | 0.867 | -22.8 | 18.9 | 41.7 | 0.213 | 26.3 | 0.024 | 0.006 | 12.1 | 0.024 |
| Siemens ADVIA Centaur XP (Liège) | 1.163 | -1.7 | 0.826 | -25.5 | 22.1 | 47.6 | 0.261 | 34.6 | 0.028 | -0.006 | 16.1 | 0.022 |
| Siemens Atellica IM | 1.146 | -4.1 | 0.839 | -27.4 | 19.2 | 46.6 | 0.163 | 21.4 | 0.041 | -0.069 | 6.1 | 0.039 |
| Siemens Dimension XL | 1.131 | -2.7 | 0.857 | -24.2 | 18.8 | 43.0 | 0.046 | 13.3 | 0.04 | 0.071 | 18.3 | 0.012 |
| Snibe (Liège) | 1.198 | -1.4 | 0.906 | -18.2 | 15.4 | 33.6 | -0.186 | 12.4 | 0.002 | -0.037 | -0.8 | 0.045 |
| Tosoh | 1.157 | -8.2 | 0.819 | -33.2 | 16.8 | 50.0 | 0.153 | 34.2 | 0.009 | 0.234 | 47.3 | -0.036 |
| **LC-MS/MS Assays** |  | | | | | | | | | | | |
| CDC | 1.092 | -1.8 | 0.974 | -10.0 | 6.4 | 16.4 | -0.094 | 0.7 | 0.012 | -0.064 | -0.1 | 0.01 |
| Chromsystems 1 | 1.189 | -5.2 | 0.966 | -15.4 | 5.0 | 20.4 | -0.095 | -1.3 | 0.018 | -0.111 | -1.5 | 0.014 |
| Chromsystems 2 | 1.087 | -3.1 | 0.970 | -11.6 | -11.8 | 5.7 | -0.095 | 21.8 | -0.008 | -0.028 | 14.3 | 0.008 |
| Cork | 1.129 | -4.8 | 0.962 | -15.2 | 11.2 | 5.5 | -0.098 | 15.8 | 0.018 | -0.089 | 13.8 | 0.018 |
| Health Canada | 1.124 | -3.7 | 0.972 | -12.6 | 5.2 | 17.8 | -0.039 | -0.2 | 0.014 | -0.037 | -1.8 | 0.014 |
| ICHT | 1.150 | -3.4 | 0.970 | -12.8 | 6.1 | 18.9 | -0.092 | 3.6 | 0.008 | -0.017 | -2.5 | 0.016 |
| Quest Diagnostics (VA) | 1.026 | -3.0 | 0.964 | -11.9 | 9.3 | 21.2 | 0.02 | 6.3 | 0.004 | -0.077 | -2.7 | 0.014 |
| U of Liège | 1.092 | -4.2 | 0.970 | -12.9 | 4.5 | 17.4 | -0.074 | -0.1 | 0.016 | -0.084 | -1.0 | 0.014 |
| U of Washington | 0.992 | -1.5 | 0.972 | -8.9 | 6.0 | 14.9 | -0.001 | 1.0 | 0.016 | -0.02 | 1.3 | 0.012 |

^a^ The 38 single-donor sample set excludes 12 samples from the 50 single-donor sample set with concentrations of 25(OH)D > 100 nmol/L.

^b^ For the regression line, Int. = y‑intercept in nmol/L

^c^ Min = minimum y‑intercept and Max = maximum y‑intercept in nmol/L; width = Min + Max values in nmol/L

^d^ Difference in the values for the 50-sample set minus the 42-sample set for slope, R^2^, and width of PI

Table S13. Commutability of SRMs for ligand binding assays using CLSI pre-set limit (8.8%) approach (50 samples)

|  | **SRM** | | | | | | | |
| --- | --- | --- | --- | --- | --- | --- | --- | --- |
| **Assay** | **2969** | **1949 NP** | **1949 T1** | **1949 T2** | **1949 T3** | **2970** | **972a L1** | **2973** |
| Roche | C | C | C | C | I | I |  |  |
| Roche (Liège) | C | C | C | C | C | C |  |  |
| Tosoh | C | C | C | C | C | NC | C | C |
| Abbott Alinity | C | C | C | C | C | NC |  |  |
| Abbott Alinity (U of Liège) | C | C | C | C | C | NC |  |  |
| Abbott Alinity (ICHT) | C | C | C | C | C | NC |  |  |
| Abbott ARCHITECT | C | C | C | C | C | NC |  |  |
| DiaSorin (U of Liège) | C | C | C | C | NC | NC |  |  |
| DiaSorin (ICHT) | C | C | C | I | NC | NC |  |  |
| IDS iSYS | C | C | C | C | C | I |  |  |
| IDS iSYS (U of Liège) | C | C | C | C | NC | C |  |  |
| bioMérieux | I | C | C | NC | NC | NC |  |  |
| bioMérieux (U of Liège) | C | C | C | NC | NC | NC |  |  |
| Siemens Dimension | C | C | C | NC | NC | NC |  |  |
| IDS-EIA | NC | NC | NC | NC | NC | NC |  |  |
| Beckman Coulter (U of Liège) | C | C | C | NC | NC | NC |  |  |
| Fujirebio | C | C | C | I | NC | C | C | C |
| Fujirebio (U of Liège) | C | C | C | C | NC | C |  |  |
| PerkinElmer | C | C | C | NC | NC | C | C | NC |
| Affimedix | C | NC | C | NC | NC | C | C | NC |
| Diazyme | C | C | NC | NC | NC | I | C | C |
| Siemens ADVIA Centaur XP | C | C | NC | NC | NC | C |  |  |
| Siemens ADVIA Centaur XP (U of Liège) | C | C | NC | NC | NC | C |  |  |
| Siemens Atellica IM | C | C | NC | NC | NC | C |  |  |
| Snibe (U of Liège) | I | C | C | C | NC | NC |  |  |

^a^ C = commutable (green), NC = noncommutable (yellow), I = Inconclusive. Blank cells indicate that the sample was not analyzed.

^b^ Assays ordered as in Table 6.

Table S14. Commutability of SRMs for LC-MS/MS assays using CLSI pre-set limit (8.8%) approach (50 samples)

|  | **SRMs^a^** | | | | | | | |
| --- | --- | --- | --- | --- | --- | --- | --- | --- |
| **LC-MS/MS Assay^b^** | **2969** | **1949 NP** | **1949 T1** | **1949 T2** | **1949 T3** | **2970** | **972a L1** | **2973** |
| Centers for Disease Control and Prevention (CDC) | C | C | C | C | C | C |  |  |
| Chromsystems (1) | C | C | C | C | C | C |  |  |
| University College Cork | C | C | C | C | C | C |  |  |
| University of Washington | C | C | C | C | C | C |  |  |
| Health Canada | C | C | C | C | C | C |  |  |
| Chromsystems (2) | C | C | C | C | C | C |  |  |
| Imperial College Healthcare Trust (ICHT) | C | C | C | C | C | C |  |  |
| Quest Diagnostics (Chantilly, VA) | C | C | C | C | C | C |  |  |
| University of Liège | C |  |  |  |  | C | C | C |

^a^ Blank cells indicate that the sample was not analyzed.

^b^ Assays ordered as in Figure 5.

Table S15. IFCC approach results for bias and uncertainty for LC-MS/MS assays

| **Laboratory** | **SRM 2969** | | **SRM 2970** | | **SRM 1949NP** | | **SRM 1949T1** | | **SRM 1949T2** | | **SRM 1949T3** | |
| --- | --- | --- | --- | --- | --- | --- | --- | --- | --- | --- | --- | --- |
|  | **d_RM_** | **U** | **d_RM_** | **U** | **d_RM_** | **U** | **d_RM_** | **U** | **d_RM_** | **U** | **d_RM_** | **U** |
| CDC | 0.0116 | 0.0206 | -0.0435 | 0.0206 | 0.0197 | 0.0215 | 0.0016 | 0.0215 | 0.0067 | 0.0215 | 0.0147 | 0.0215 |
| Chromsystems 1 | 0.0277 | 0.0225 | 0.0013 | 0.0225 | 0.0249 | 0.0234 | 0.0075 | 0.0234 | 0.0250 | 0.0234 | 0.0198 | 0.0234 |
| Chromsystems 2 | -0.0204 | 0.0345 | 0.0543 | 0.0345 | 0.0194 | 0.0353 | 0.0551 | 0.0353 | 0.0451 | 0.0353 | 0.0290 | 0.0353 |
| U of Cork | -0.0066 | 0.0315 | 0.0422 | 0.0315 | 0.0116 | 0.0321 | 0.0067 | 0.0321 | 0.0137 | 0.0321 | 0.0315 | 0.0321 |
| Health Canada | -0.0378 | 0.0277 | -0.0332 | 0.0277 | 0.0181 | 0.0284 | -0.0554 | 0.0284 | -0.0361 | 0.0284 | -0.1034 | 0.0284 |
| Imperial College | -0.0816 | 0.0428 | -0.2476 | 0.0428 | -0.0065 | 0.0428 | -0.0409 | 0.0428 | -0.1050 | 0.0428 | -0.1812 | 0.0428 |
| U of Liège | 0.0416 | 0.0572 | 0.0011 | 0.0572 |  |  |  |  |  |  |  |  |
| Quest (VA) | -0.1740 | 0.0749 | 0.0975 | 0.0749 | -0.0263 | 0.0752 | -0.0613 | 0.0752 | -0.0609 | 0.0752 | -0.0849 | 0.0752 |
| U of Washington | 0.0335 | 0.0427 | 0.0217 | 0.0427 | 0.0209 | 0.0431 | 0.0020 | 0.0431 | 0.0327 | 0.0431 | 0.0145 | 0.0431 |
|  | | | | | | | | | | | | |
| **Laboratory** | **SRM 2973** | | **SRM 972a L2** | |  |  |  |  |  |  |  |  |
|  | **d_RM_** | **U** | **d_RM_** | **U** |  |  |  |  |  |  |  |  |
| U of Liège | -0.0293 | 0.0572 | -0.068 | 0.0572 |  |  |  |  |  |  |  |  |

^a^ Blank cells in table indicate that the SRM was not analyzed.

Table S16. IFCC approach results for bias and uncertainty for ligand binding assays

| **Laboratory/Assay** | **SRM 2969** | | **SRM 2970** | | **SRM 1949NP** | | **SRM 1949T1** | | **SRM 1949T2** | | **SRM 1949T3** | |
| --- | --- | --- | --- | --- | --- | --- | --- | --- | --- | --- | --- | --- |
|  | **d_RM_** | **U** | **d_RM_** | **U** | **d_RM_** | **U** | **d_RM_** | **U** | **d_RM_** | **U** | **d_RM_** | **U** |
| Abbott Alinity | -0.0375 | 0.0596 | -0.3607 | 0.0596 | 0.0562 | 0.0640 | 0.0087 | 0.0640 | 0.0369 | 0.0640 | -0.0255 | 0.0640 |
| Abbot Alinity (U of Liège) | -0.0262 | 0.0642 | -0.3372 | 0.0642 | 0.0921 | 0.0644 | 0.0095 | 0.0644 | 0.0363 | 0.0644 | -0.0041 | 0.0644 |
| Abbott Alinity (ICHT) | 0.0214 | 0.0583 | -0.3236 | 0.0583 | 0.1130 | 0.0608 | 0.0629 | 0.0608 | 0.0396 | 0.0608 | 0.0076 | 0.0608 |
| Abbott ARCHITECT | -0.0377 | 0.0589 | -0.3731 | 0.0589 | 0.0603 | 0.0593 | 0.0145 | 0.0593 | 0.0157 | 0.0593 | -0.0189 | 0.0593 |
| Affimedix | -0.1677 | 0.0726 | 0.1408 | 0.0726 | 0.2893 | 0.0729 | 0.0494 | 0.0729 | -0.1869 | 0.0729 | -0.2270 | 0.0729 |
| Beckman Coulter (U of Liège) | -0.0195 | 0.0855 | -0.1142 | 0.0855 | -0.1137 | 0.0857 | -0.0824 | 0.0857 | -0.1926 | 0.0857 | -0.3051 | 0.0857 |
| bioMérieux | -0.1948 | 0.0759 | 0.3944 | 0.0759 | 0.0298 | 0.0761 | 0.0709 | 0.0761 | 0.1574 | 0.0761 | 0.2243 | 0.0761 |
| bioMérieux (U of Liège) | -0.1199 | 0.0699 | -0.3773 | 0.0699 | 0.0269 | 0.0701 | -0.0801 | 0.0701 | -0.1329 | 0.0701 | -0.2217 | 0.0701 |
| DiaSorin (U of Liège) | -0.0530 | 0.0573 | -0.1800 | 0.0573 | 0.1075 | 0.0576 | 0.0608 | 0.0576 | 0.0341 | 0.0576 | -0.0769 | 0.0576 |
| DiaSorin (ICHT) | -0.0816 | 0.0428 | -0.2476 | 0.0428 | -0.0065 | 0.0431 | -0.0409 | 0.0431 | -0.1050 | 0.0431 | -0.1812 | 0.0431 |
| Diazyme | -0.2436 | 0.0687 | -0.0679 | 0.0687 | -0.0954 | 0.0689 | -0.1738 | 0.0689 | -0.3705 | 0.0689 | -0.2938 | 0.0689 |
| Fujirebio | -0.0483 | 0.0198 | -0.0193 | 0.0198 | -0.0202 | 0.0206 | -0.0651 | 0.0206 | -0.1129 | 0.0206 | -0.1474 | 0.0206 |
| Fujirebio (U of Liège) | -0.0545 | 0.0354 | -0.0101 | 0.0354 | 0.0091 | 0.0358 | -0.0470 | 0.0358 | -0.1053 | 0.0358 | -0.1310 | 0.0358 |
| IDS EIA | -0.1355 | 0.0946 | -0.2771 | 0.0946 | -0.0145 | 0.0948 | -0.1234 | 0.0948 | -0.2233 | 0.0948 | -0.2267 | 0.0946 |
| IDS iSYS | -0.0519 | 0.0446 | -0.1361 | 0.0446 | -0.0041 | 0.0450 | -0.0388 | 0.0450 | -0.0763 | 0.0450 | -0.1304 | 0.0450 |
| IDSiSYS (U of Liège) | -0.0560 | 0.0415 | -0.1143 | 0.0415 | -0.0107 | 0.0420 | -0.0618 | 0.0420 | -0.1057 | 0.0420 | -0.1606 | 0.0420 |
| PerkinElmer | -0.0731 | 0.0377 | 0.0132 | 0.0377 | 0.1130 | 0.0381 | -0.0777 | 0.0381 | -0.2778 | 0.0381 | -0.3587 | 0.0381 |
| Roche | 0.0162 | 0.0458 | -0.0906 | 0.0458 | -0.0490 | 0.0461 | -0.0129 | 0.0461 | -0.0574 | 0.0461 | -0.1106 | 0.0461 |
| Roche (U of Liège) | 0.0829 | 0.1351 | -0.0695 | 0.1351 | -0.0371 | 0.1352 | 0.0228 | 0.1352 | -0.0311 | 0.1352 | -0.0814 | 0.1352 |
| Siemens ADVIA Centaur XP | -0.0441 | 0.0645 | -0.0102 | 0.0645 | -0.0162 | 0.0648 | -0.2234 | 0.0648 | -0.3950 | 0.0648 | -0.4757 | 0.0648 |
| Siemens ADVIA Centaur XP (U of Liège) | -0.0571 | 0.0912 | -0.0362 | 0.0912 | -0.0435 | 0.0914 | -0.2340 | 0.0914 | -0.4117 | 0.0914 | -0.4743 | 0.0914 |
| Siemens Atellica IM | 0.0099 | 0.0654 | 0.0438 | 0.0654 | 0.0378 | 0.0657 | -0.1694 | 0.0657 | -0.3410 | 0.0657 | -0.4217 | 0.0657 |
| Siemens Dimension | -0.1032 | 0.0518 | -0.2147 | 0.0518 | 0.000 | 0.0522 | -0.0597 | 0,0522 | -0.1957 | 0.0522 | -0.2899 | 0.0522 |
| Snibe (U of Liège) | 0.0292 | 0.0594 | -0.0761 | 0.0594 | 0.1969 | 0.0596 | 0.1760 | 0.0596 | 0.0569 | 0.0596 | -0.0812 | 0.0596 |
| Tosoh | -0.0601 | 0.0688 | -0.1032 | 0.0688 | 0.0156 | 0.0691 | 0.1284 | 0.0691 | 0.1066 | 0.0691 | 0.0705 | 0.0691 |

Table S17. IFCC approach results for bias and uncertainty for ligand binding assays for additional SRMs

| **Laboratory** | **SRM 972a L1** | | **SRM 2973** | |
| --- | --- | --- | --- | --- |
|  | **d_RM_** | **U** | **d_RM_** | **U** |
| Affimedix | -0.0144 | 0.0726 | -0.0452 | 0.0726 |
| Diazyme | 0.0442 | 0.0687 | 0.0890 | 0.0687 |
| Fujirebio | -0.0510 | 0.0198 | -0.0457 | 0.0198 |
| PerkinElmer | -0.0535 | 0.0377 | -0.1012 | 0.0377 |
| Tosoh | 0.0067 | 0.0688 | 0.0919 | 0.0688 |

Table S18. Multivariable linear regression analysis for LC-MS/MS assays for 50 single-donor samples^a^

| **Lab** | **Assay** | **R^2^** | **25(OH)D_2_** | **SE** | **25(OH)D_3_** | **SE** | **3-epi-25(OH)D_3_** | **SE** | **24R,25(OH)_2_D_3_** | **SE** |
| --- | --- | --- | --- | --- | --- | --- | --- | --- | --- | --- |
| CDC | LC-MS/MS | 0.988 | 0.964 | 0.019 | 0.937 | 0.041 | 0.68 | 0.44 | 0.36 | 0.38 |
| Chromsystems | Chromsystems 1 | 0.987 | 1.094 | 0.02 | 1.014 | 0.050 | 1.06 | 0.52 | -0.10 | 0.44 |
| Chromsystems | Chromsystems 2 | 0.974 | 0.929 | 0.028 | 0.929 | 0.060 | 0.48 | 0.60 | 0.80 | 0.56 |
| Univ. College Cork | LC-MS/MS | 0.982 | 1.016 | 0.024 | 0.942 | 0.053 | 1.20 | 0.57 | 0.05 | 0.49 |
| Health Canada | LC-MS/MS | 0.988 | 1.069 | 0.021 | 1.017 | 0.045 | 0.73 | 0.49 | 0.20 | 0.42 |
| ICHT | LC-MS/MS | 0.990 | 0.985 | 0.018 | 1.032 | 0.040 | 1.06 | 0.43 | 0.17 | 0.37 |
| Quest Diagnostics (VA) | LC-MS/MS | 0.985 | 1.087 | 0.023 | 0.869 | 0.049 | 1.60 | 0.53 | -0.06 | 0.45 |
| Univ. of Liège | LC-MS/MS | 0.988 | 1.017 | 0.019 | 0.923 | 0.042 | 1.01 | 0.45 | 0.09 | 0.39 |
| Univ. of Washington | LC-MS/MS | 0.992 | 1.003 | 0.015 | 1.005 | 0.033 | 1.45* | 0.36 | -1.25* | 0.31 |

Color Key for *X_1_*, *X_2_*, *X_1_*, and *X_4_* from multivariable regression equation:

Estimated as expected (0.0.85 to 1.15) and significant contribution to estimate (p < 0.0001)

Underestimated (< 0.85) and significant contribution to estimate (*p* < 0.0001)

Overestimated (> 1.15) and significant contribution to estimate (*p* < 0.0001)

Significant contribution to the estimate (*p* < 0.0001)

No significant contribution to the estimate (*p* > 0.0001)

* Indicates contribution to the estimate (0.0001 > *p* <0.001)

Table S19. Bias relative to NIST target values for ligand binding assay

| **Assay**  **Manufacturer** | **Assay Kit Name** | **Mean % bias^a^** | **Mean absolute % bias^b^** | **% samples with bias**  **< ±5%^c^** | **CDC VDSCP^d^** | |
| --- | --- | --- | --- | --- | --- | --- |
|  |  |  |  |  | **Median**  **% < ±5%^d^** | **Range^d^** |
| Abbott | Alinity 25-OH Vitamin D | 4.0 | 11.5 | 38% | 37% (4) | 25% - 45% |
| Abbott (ICHT) | Alinity 25-OH Vitamin D | 2.2 | 12.2 | 36% |  |  |
| Abbott (U of Liège) | Alinity 25-OH Vitamin D | 2.0 | 12.3 | 38% |  |  |
| Abbott | Architect 25-OH Vitamin D | 0.2 | 13.0 | 32% | 35% (8) | 28% - 40% |
| Affimedix | MicrO-D | -2.6 | 13.9 | 22% | 35% (1) | 35% |
| Beckman Coulter | Access 25(OH)Vitamin D Total | -1.8 | 13.3 | 24% |  |  |
| bioMérieux | VIDAS 25 OH Vitamin D Total | 1.1 | 19.9 | 12% |  |  |
| bioMérieux (U of Liège) | VIDAS 25 OH Vitamin D Total | -2.0 | 19.0 | 14% |  |  |
| DiaSorin (U of Liège) | 25OH Vitamin D Total | -4.6 | 12.9 | 18% | 21% (14) | 10% - 30% |
| DiaSorin (ICHT) | 25OH Vitamin D Total | -15.0 | 18.6 | 8% |  |  |
| Diazyme | Diazyme EZ Vitamin D Assay | 2.8 | 17.6 | 22% | 13.5% (10) | 8% - 21% |
| Fujirebio | Lumipulse G 25-OH Vitamin D | -5.5 | 6.6 | 44% | 45% | 45% |
| Fujirebio (U of Liège) | Lumipulse G 25-OH Vitamin D | -1.2 | 5.3 | 54% |  |  |
| IDS-EIA | 25‑Hydroxy Vitamin D^S^ EIA | -14.9 | 22.9 | 12% | 26% (14) | 18% - 35% |
| IDS-iSYS | IDS 25VitD^S^ | 1.9 | 8.7 | 34% | 34% (14) | 22% - 45% |
| IDS-iSYS (U of Liège) | IDS 25VitD^S^ | -0.5 | 8.4 | 40% |  |  |
| PerkinElmer | Total 25OH Vitamin D ELISA | 6.8 | 10.8 | 28% | 28% (1) | 28% |
| Roche | Elecsys Vitamin D Total III | -0.6 | 8.6 | 40% | 33.5% (10) | 15% - 42% |
| Roche (U of Liège) | Elecsys Vitamin D Total III | -2.5 | 10.9 | 32% |  |  |
| Siemens ADVIA Centaur XP | Vitamin D Total (VitD) | -16.0 | 19.1 | 20% | 21% (14) | 15% - 28% |
| Siemens ADVIA Centaur XP (U of Liège) | Vitamin D Total (VitD) | -17.4 | 21.9 | 12% |  |  |
| Siemens Attelica | Vitamin D Total (VitD) | -10.7 | 16.5 | 24% | 32% (15) | 18% - 38% |
| Siemens Dimension | LOCI Vitamin D Total Assay | -10.1 | 15.6 | 32% | 42.5% (14) | 32% - 50% |
| Snibe (U of Liège) | 25-OH Vitamin D | -14.3 | 16.0 | 20% | 30 (2) | 28% - 32% |
| Tosoh | ST AIA-PACK 25-OH Vitamin D | -5.5 | 18.1 | 28% | 18 (12) | 12% - 28% |

^a^ Mean % bias from NIST target value based on mean of duplicate measurements.

^b^ Mean absolute bias from NIST target value.

^c^ % of individual sample measurements (mean of duplicate measurements) that were **< ±**5% bias from NIST target values.

^d^ CDC Vitamin D Standardization – Certification Program (VDSCP);

results from www.cdc.gov/labstandards/csp/pdf/hs/CDC_Certified_Vitamin_D_Assays-508.pdf; median value of % pass rate for quarterly studies since 2019; number in parentheses is the number of quarterly exercises for which results are reported; range is the range of % pass rate reported for quarterly exercises. Blank cells indicate that the assay was not evaluated in the CDC VDSCP.

Table S20. Bias relative to NIST target values for LC-MS/MS assays

| **Laboratory** | **Assay** | **Mean % bias** | **Mean % Absolute bias** | **Mean %**  **< ±5% bias** | **CDC VDSCP** | |
| --- | --- | --- | --- | --- | --- | --- |
|  |  |  |  |  | **Median**  **% < ±5%** | **Range** |
| CDC | LC-MS/MS | -5.2 | 6.1 | 50% | 80% | 65% - 92% |
| Chromsystems Instruments & Chemicals GmbH | Chromsystems 1  LC-MS/MS | -8.7 | 8.9 | 24% |  |  |
| Chromsystems Instruments & Chemicals GmbH | Chromsystems 2  LC-MS/MS | -2.4 | 5.5 | 58% |  |  |
| Health Canada | LC-MS/MS | -6.0 | -6.6 | 42% | 68% | 40% - 80% |
| Imperial College Healthcare Trust | LC-MS/MS | -8.3 | 8.8 | 22% |  |  |
| University College Cork | LC-MS/MS | -4.0 | 6.0 | 48% |  |  |
| University of Liege | LC-MS/MS | -1.3 | 4.0 | 76% | 74% | 57% - 92% |
| Quest Diagnostics (Chantilly, VA) | LC-MS/MS | 1.8 | 6.3 | 42% |  |  |
| University of Washington | LC-MS/MS | 3.1 | 4.7 | 66% |  |  |

^a^ Mean % bias from NIST target value based on mean of duplicate measurements.

^b^ Mean absolute bias from NIST target value.

^c^ % of individual sample measurements (mean of duplicate measurements) that were **< ±**5% bias from NIST target values.

^d^ CDC Vitamin D Standardization – Certification Program (VDSCP);

results from www.cdc.gov/labstandards/csp/pdf/hs/CDC_Certified_Vitamin_D_Assays-508.pdf; median value of % pass rate for quarterly studies since 2019; number in parentheses is the number of quarterly exercises for which results are reported; range is the range of % pass rate reported for quarterly exercises. Blank cells indicate that the assay was not evaluated in the CDC VDSCP.

**References**

[1] W.J. Korzun, G. Nilsson, L.M. Bachmann, G.L. Myers, I. Sakurabayashi, K. Nakajima, M. Nakamura, R.D. Shamburek, A.T. Remaley, W.G. Miller, Difference in bias approach for commutability assessment: Application to frozen pools of human serum measured by 8 direct methods for HDL and LDL cholesterol, Clin. Chem., 61 (2015) 1107-1113.

[2] V. Delatour, Q.D. Liu, H.W. Vesper, L.-L.W. Grp, Commutability assessment of external quality assessment materials with the difference in bias approach: Are acceptance criteria based on medical requirements too strict?, Clin. Chem., 62 (2016) 1670-1671.

[3] H. Liu, L.K. Wong, S.R. Yong, Q.D. Liu, T.L. Teo, T.K. Lee, T.P. Loh, S.K. Sethi, M.S. Wong, S.M.D. Cosio, C.K.M. Ho, J.W.S. Setoh, S.F.M. Lim, G.L.L. Lee, H. Khalid, S. Lim, C.W. Lam, M.Y. Lee, C.P. Yeo, P.R. Chincholkar, R. Hawkins, B.Y. Ng, Commutable whole blood reference materials for hemoglobin A(1c) validated on multiple clinical analyzers, Clin. Chem. Lab. Med., 57 (2019) 648-658.

[4] S.L. Zhang, F. Cheng, H. Wang, J.P. Wen, J. Zeng, C.B. Zhang, W.S. Liu, N. Wang, T.T. Jia, M. Wang, R. Zhang, Y.H. Yue, J. Xu, Z.Y. Wang, Y.L. Li, W.X. Chen, Q.T. Wang, Comparability of thyroid-stimulating hormone immunoassays using fresh frozen human sera and external quality assessment data, PLoS One, 16 (2021) 13.

[5] G.F. Hu, Z.Y. Sun, Z.Y. Yu, C.B. Li, Y.H. Liu, M.T. Peng, Comparability of sample results and commutability of reference materials among different measurement procedures for protein C activity assays, Clin. Chim. Acta, 524 (2022) 164-170.

[6] T.T. Xing, J.Y. Liu, H.F. Sun, Y.H. Gao, Y. Ju, X.L. Liu, D.W. Song, Commutability assessment of reference materials for homocysteine, Clin. Chem. Lab. Med., 60 (2022) 1562-1569.

[7] Q.C. Long, T.Q. Qi, T.J. Zhang, J. Wang, J. Zeng, Y. Yan, M. Wang, W. Huang, H.J. Zhao, W.X. Chen, C.B. Zhang, Commutability assessment of candidate external quality assessment materials for aminotransferase activity measurements based on different approaches in China, Ann. Lab. Med., 41 (2021) 68-+.

[8] X.L. Yi, Y.F. Wang, T.J. Zhang, J. Zeng, H.J. Zhao, W.Y. Zhou, J.T. Zhang, Y. Yan, W.X. Chen, C.B. Zhang, Commutability of possible external quality assessment materials for progesterone measurement, Clin. Biochem., 87 (2021) 39-45.

[9] H. Liu, C.Y. Ng, Q.D. Liu, T.L. Teo, T.P. Loh, M.S. Wong, S.K. Sethi, J.G. Tan, P.Y. Heng, S. Saw, L.C.W. Lam, J.M.Y. Lee, K.B. Khaled, H.P. Phyu, N.W.T. Ong, Commutability assessment of human urine certified reference materials for albumin and creatinine on multiple clinical analyzers using different statistical models, Anal. Bioanal. Chem., 415 (2023) 787-800.

[10] Y.H. Deng, Q.X. Liu, Z.N. Liu, H.J. Zhao, W.Y. Zhou, C.B. Zhang, Commutability assessment of processed human plasma samples for normetanephrine and metanephrine measurements based on the candidate reference measurement procedure, Ann. Lab. Med., 42 (2022) 575-+.

[11] Y.H. Deng, C. Zhang, J. Wang, J. Zeng, J.T. Zhang, T.J. Zhang, H.J. Zhao, M. Li, Y. Zhao, W. Gan, Y. Shao, H.Y. Yu, W.Y. Zhou, C.B. Zhang, Application of serum pools in insulin harmonization: Commutability and stability, Annals of Clinical Biochemistry, 60 (2023) 199-207.

[12] Y.H. Deng, C. Zhang, B.Q. Li, J. Wang, J. Zeng, J.T. Zhang, T.J. Zhang, H.J. Zhao, W.Y. Zhou, C.B. Zhang, Exploration of suitable external quality assessment materials for serum C-peptide measurement, Clin. Chem. Lab. Med., 61 (2023) 1597-1604.

[13] I. Dikaios, H. Althaus, E. Angles-Cano, U. Ceglarek, S. Coassin, C.M. Cobbaert, V. Delatour, B. Dieplinger, M. Grimmler, A.N. Hoofnagle, G.M. Kostner, F. Kronenberg, Z. Kuklenyik, A.N. Lyle, U. Prinzing, L.R. Ruhaak, H. Scharnagl, H.W. Vesper, L. Deprez, I.W.G.A. Mass, Commutability assessment of candidate reference materials for lipoprotein(a) by comparison of a MS-based candidate reference measurement procedure with immunoassays, Clin. Chem., 69 (2023) 262-272.

[14] Z.N. Liu, L.Z. Jin, Z.J. Ma, X. Nizhamuding, J. Zeng, T.J. Zhang, J.T. Zhang, W.Y. Zhou, C.B. Zhang, Commutability assessment of candidate reference materials for plasma renin activity measurement: current challenges, Clin. Chem. Lab. Med., (2023) 10.

[15] R. Zhang, Z.Z. Xu, R. Zhao, W.X. Fu, Y.C. Song, Q.T. Wang, Y.H. Yue, Accurate method for value assignment of carcinoembryonic antigen reference materials, J. Clin. Lab. Anal., (2023) 11.

[16] A.N. Lyle, J.R. Budd, V.M. Kennerley, B.N. Smith, U. Danilenko, C.M. Pfeiffer, H.W. Vesper, Assessment of WHO 07/202 reference material and human serum pools for commutability and for the potential to reduce variability among soluble transferrin receptor assays, Clin. Chem. Lab. Med., (2023) 11.

[17] L.E. Briggs, J.K. Whitewood, E.L. Williams, Analytical variation concerning total 25-hydroxyvitamin D measurement, where are we now? A DEQAS review of current assay performance, J. Steroid Biochem. Mol. Biol., 231 (2023) 8.

[18] C. Le Goff, S. Peeters, Y. Crine, P. Lukas, J.C. Souberbielle, E. Cavalier, Evaluation of the cross-reactivity of 25-hydroxyvitamin D_2_ on seven commercial immunoassays on native samples, Clin. Chem. Lab. Med., 50 (2012) 2031-2032.

[19] E. Moreau, S. Bacher, S. Mery, C. Le Goff, N. Piga, M. Vogeser, M. Hausmann, E. Cavalier, Performance characteristics of the VIDAS (R) 25-OH Vitamin D Total assay - comparison with four immunoassays and two liquid chromatography-tandem mass spectrometry methods in a multicentric study, Clin. Chem. Lab. Med., 54 (2016) 45-53.

[20] F.B. Saida, M. Padilla-Chee, C. Dou, C. Yuan, First two-reagent vitamin D assay for general clinical chemistry, Clin. Biochem., 55 (2018) 28-35.

[21] J. Favresse, M. Fangazio, F. Cotton, F. Wolff, Evaluation of four automated clinical analyzers for the determination of total 25(OH)D in comparison to a certified LC-MS/MS, Clin. Chem. Lab. Med., 61 (2023) 1420-1427.

[22] L.S. Li, Q. Zeng, J.J. Yuan, Z.J. Xie, Performance evaluation of two immunoassays for 25-hydroxyvitamin D, Journal of Clinical Biochemistry and Nutrition, 58 (2016) 186-192.

[23] J.M.W. van den Ouweland, A.M. Beijers, H. van Daal, M. Elisen, G. Steen, J.P.M. Wielders, Evaluation of 3-epi-25-hydroxyvitamin D-3 cross-reactivity in the Roche Elecsys Vitamin D Total protein binding assay, Clin. Chem. Lab. Med., 52 (2014) 373-380.

[24] J.H. Lee, J. Do Seo, K. Lee, E.Y. Roh, Y.M. Yun, Y.W. Lee, S.E. Cho, J. Song, Multicenter comparison of analytical interferences of 25-OH vitamin D immunoassay and mass spectrometry methods by endogenous interferents and cross-reactivity with 3-epi-25-OH-vitamin D_3_, Pract. Lab. Med., 38 (2024).

[25] E. Cavalier, P. Lukas, Y. Crine, S. Peeters, A. Carlisi, C. Le Goff, R. Gadisseur, P. Delanaye, J.C. Souberbielle, Evaluation of automated immunoassays for 25(OH)-vitamin D determination in different critical populations before and after standardization of the assays, Clin. Chim. Acta, 431 (2014) 60-65.

[26] M.J.W. Janssen, J.P.M. Wielders, C.C. Bekker, L.S.M. Boesten, M.M. Buijs, A.C. Heijboer, F.A.L. van der Horst, F.J. Loupatty, J.M.W. van den Ouweland, Multicenter comparison study of current methods to measure 25-hydroxyvitamin D in serum, Steroids, 77 (2012) 1366-1372.

[27] J. Freeman, K. Wilson, R. Spears, V. Shalhoub, P. Sibley, Performance evaluation of four 25-hydroxyvitamin D assays to measure 25-hydroxyvitamin D-2, Clin. Biochem., 48 (2015) 1097-1104.

[28] E. Garnett, J.L. Li, D. Rajapakshe, E. Tam, Q.H. Meng, S. Devaraj, Efficacy of two vitamin D immunoassays to detect 25-OH vitamin D_2_ and D_3_, Pract. Lab. Med., 17 (2019) 4.

[29] S.P. Wyness, J.A. Straseski, Performance characteristics of six automated 25-hydroxyvitamin D assays: Mind your 3s and 2s, Clin. Biochem., 48 (2015) 1089-1096.

[30] W. Annema, A. Nowak, A. von Eckardstein, L. Saleh, Evaluation of the new restandardized Abbott Architect 25-OH Vitamin D assay in vitamin D-insufficient and vitamin D-supplemented individuals, J. Clin. Lab. Anal., 32 (2018) 5.

[31] S.A. Wise, J.E. Camara, C.T. Sempos, C.Q. Burdette, G. Hahm, F. Nalin, A.J. Kuszak, J. Merkel, R. Durazo-Arvizu, E.L. Williams, C. Popp, C. Beckert, C. Schultess, G. Van Slooten, C. Tourneur, C. Pease, R. Kaul, A. Villarreal, M.C. Batista, H. Pham, A. Bennett, E. Jansen, D.A. Khan, M. Kilbane, J. Freeman, N. Parker, J. Yuan, S. Mushtaq, C. Simpson, P. Lukas, E. Cavalier, Interlaboratory comparison of 25-hydroxyvitamin D assays: Vitamin D Standardization Program (VDSP) intercomparison study 2 - Part 2 Ligand binding assays – Impact of 25-hydroxyvitamin D_2_ and 24R,25-dihydroxyvitamin D_3_ on assay performance, Anal. Bioanal. Chem., 414 (2022) 351-366.

[32] E. Cavalier, P. Lukas, A.C. Bekaert, A. Carlisi, C. Le Goff, P. Delanaye, J.C. Souberbielle, Analytical and clinical validation of the new Abbot Architect 25(OH) D assay: fit for purpose?, Clin. Chem. Lab. Med., 55 (2017) 378-384.

[33] F. Trimboli, S. Rotundo, S. Armili, S. Mimmi, F. Lucia, N. Montenegro, G.C. Antico, A. Cerra, M. Gaetano, F. Galato, L.G. Carinci, D. Iania, S. Mancuso, M. Martucci, C. Teti, M. Greco, G. Cuda, E. Angotti, Serum 25-hydroxyvitamin D measurement: Comparative evaluation of three automated immunoassays, Pract. Lab. Med., 26 (2021).

[34] M. Rahme, L. Al-Shaar, R. Singh, R. Baddoura, G. Halaby, A. Arabi, R.H. Habib, R. Daher, D. Bassil, K. El-Ferkh, M. Hoteit, G.E. Fuleihan, Limitations of platform assays to measure serum 25OHD level impact on guidelines and practice decision making, Metabolism-Clinical and Experimental, 89 (2018) 1-7.

[35] L. Zhang, Q.C. Long, J.T. Zhang, J. Zeng, T.J. Zhang, C.B. Zhang, Ligand binding assay-related underestimation of 25-hydroxyvitamin D in pregnant women exaggerates the prevalence of vitamin D insufficiency, Clin. Chem. Lab. Med., 61 (2023) E29-E32.

[36] E. Cavalier, P. Lukas, A.C. Bekaert, S. Peeters, C. Le Goff, E. Yayo, P. Delanaye, J.C. Souberbielle, Analytical and clinical evaluation of the new Fujirebio Lumipulse (R) G non-competitive assay for 25(OH)-vitamin D and three immunoassays for 25(OH) D in healthy subjects, osteoporotic patients, third trimester pregnant women, healthy African subjects, hemodialyzed and intensive care patients, Clin. Chem. Lab. Med., 54 (2016) 1347-1355.

[37] Y. Chen, L. Kinney, A. Bozovic, H. Smith, H. Tarr, E.P. Diamandis, A. LeBlanc, Performance evaluation of Siemens ADVIA Centaur and Roche MODULAR Analytics E170 Total 25-OH Vitamin D assays, Clin. Biochem., 45 (2012) 1485-1490.

[38] S.A. Wise, J.E. Camara, C.T. Sempos, C.Q. Burdette, G. Hahm, F. Nalin, A.J. Kuszak, J. Merkel, R. Durazo-Arvizu, A.N. Hoofnagle, E.L. Williams, F. Ivison, R. Fischer, J.M.W. Van den Ouweland, C.S. Ho, E.W.K. Law, J.-N. Simard, R. Gonthier, B. Holmquist, S. Meadows, L. Cox, K. Robyak, M.H. Creer, R. Fitzgerald, M.W. Clarke, N. Breen, P. Lukas, E. Cavalier, Interlaboratory comparison of 25-hydroxyvitamin D assays: Vitamin D Standardization Program (VDSP) intercomparison study 2 – Part 1 Liquid chromatography – tandem mass spectrometry (LC-MS/MS) assays – Impact of 3-epi-25-hydroxyvitamin D_3_ on assay performance, Anal. Bioanal. Chem., 414 (2022) 333-349.

[39] G. Hahm, M. Nelson, J. Camara, B. Toman, Certification of Standard Reference Materials 2969 and 2970: Vitamin D Metabolites in Frozen Human Serum (Total 25-Hydroxyvitamin D Low Level) and (25-Hydroxyvitamin D_2_ High Level), National Institute of Standards and Technology (NIST), Gaithersburg, MD, 2021.

[40] K.W. Phinney, S.S.C. Tai, M. Bedner, J.E. Camara, R.R.C. Chia, L.C. Sander, K.E. Sharpless, S.A. Wise, J.H. Yen, R.L. Schleicher, M. Chaudhary-Webb, K.L. Maw, Y. Rahmani, J.M. Betz, J. Merkel, C.T. Sempos, P.M. Coates, R.A. Durazo-Arvizu, K. Sarafin, S.P.J. Brooks, Development of an improved Standard Reference Material for vitamin D metabolites in human serum, Anal. Chem., 89 (2017) 4907-4913.

[41] S.S.C. Tai, M.A. Nelson, M. Bedner, B.E. Lang, K.W. Phinney, L.C. Sander, J.H. Yen, J.M. Betz, C.T. Sempos, S.A. Wise, Development of Standard Reference Material (SRM) 2973 vitamin D metabolites in frozen human serum (high level), J. AOAC Int., 100 (2017) 1294-1303.

[42] A.S.P. Boggs, L.E. Kilpatrick, C.Q. Burdette, D.S. Tevis, Z.A. Fultz, M.A. Nelson, J.M. Jarrett, J.V. Kemp, R.J. Singh, S.K.G. Grebe, S.A. Wise, B.L. Kassim, S.E. Long, Development of a pregnancy-specific reference material for thyroid biomarkers, vitamin D, and nutritional trace elements in serum, Clin. Chem. Lab. Med., 59 (2021) 671-679.

**Figure Captions (Figures S1 to S29)**

Figure S1. Assessment of commutability using the CLSI 95% PI approach for (A and B) Abbott Alinity assay (ICHT) and (C and D) Abbott Alinity (Liège) assay using both the 50- and 42-single donor sets. The black circles (open and filled) are the single-donor samples. The black filled circles represent the single-donor samples with high 25(OH)D_2_ concentrations (>30 nmol/L). The black solid line is the Ordinary Deming regression line, and the red dashed lines are the 95% PI. The blue dotted line is the identity line (y = x). The red triangles are the SRM samples which are identified in the plots for the 42-sample set.

Figure S2. Assessment of commutability using the CLSI 95% PI approach for (A and B) Abbott ARCHITECT assay and (C and D) Affimedix assay using both the 50- and 42-single donor sets. The black circles (open and filled) are the single-donor samples. The black filled circles represent the single-donor samples with high 25(OH)D_2_ concentrations (>30 nmol/L). The black solid line is the Ordinary Deming regression line, and the red dashed lines are the 95% PI. The blue dotted line is the identity line (y = x). The red triangles are the SRM samples which are identified in the plots for the 42-sample set.

Figure S3. Assessment of commutability using the CLSI 95% PI approach for (A and B) bioMérieux assay and (C and D) bioMérieux (Liège) assay using both the 50- and 42-single donor sets. The black circles (open and filled) are the single-donor samples. The black filled circles represent the single-donor samples with high 25(OH)D_2_ concentrations (>30 nmol/L). The black solid line is the Ordinary Deming regression line, and the red dashed lines are the 95% PI. The blue dotted line is the identity line (y = x). The red triangles are the SRM samples which are identified in the plots for the 42-sample set.

Figure S4. Assessment of commutability using the CLSI 95% PI approach for (A and B) DiaSorin (ICHT) assay and (C and D) DiaSorin (Liège) assay using both the 50- and 42-single donor sets. The black circles (open and filled) are the single-donor samples. The black filled circles represent the single-donor samples with high 25(OH)D_2_ concentrations (>30 nmol/L). The black solid line is the Ordinary Deming regression line, and the red dashed lines are the 95% PI. The blue dotted line is the identity line (y = x). The red triangles are the SRM samples which are identified in the plots for the 42-sample set.

Figure S5. Assessment of commutability using the CLSI 95% PI approach for (A and B) Beckman Coulter assay and (C and D) Diazyme assay using both the 50- and 42-single donor sets. The black circles (open and filled) are the single-donor samples. The black filled circles represent the single-donor samples with high 25(OH)D_2_ concentrations (>30 nmol/L). The black solid line is the Ordinary Deming regression line, and the red dashed lines are the 95% PI. The blue dotted line is the identity line (y = x). The red triangles are the SRM samples which are identified in the plots for the 42-sample set.

Figure S6. Assessment of commutability using the CLSI 95% PI approach for (A and B) Fujirebio assay and (C and D) Fujirebio (Liège) assay using both the 50- and 42-single donor sets. The black circles (open and filled) are the single-donor samples. The black filled circles represent the single-donor samples with high 25(OH)D_2_ concentrations (>30 nmol/L). The black solid line is the Ordinary Deming regression line, and the red dashed lines are the 95% PI. The blue dotted line is the identity line (y = x). The red triangles are the SRM samples which are identified in the plots for the 42-sample set.

Figure S7. Assessment of commutability using the CLSI 95% PI approach for (A and B) IDS-EIA assay and (C and D) PerkinElmer assay using both the 50- and 42-single donor sets. The black circles (open and filled) are the single-donor samples. The black filled circles represent the single-donor samples with high 25(OH)D_2_ concentrations (>30 nmol/L). The black solid line is the Ordinary Deming regression line, and the red dashed lines are the 95% PI. The blue dotted line is the identity line (y = x). The red triangles are the SRM samples which are identified in the plots for the 42-sample set.

Figure S8. Assessment of commutability using the CLSI 95% PI approach for (A and B) IDS-iSYS assay and (C and D) IDS-iSYS (Liège) assay using both the 50- and 42-single donor sets. The black circles (open and filled) are the single-donor samples. The black filled circles represent the single-donor samples with high 25(OH)D_2_ concentrations (>30 nmol/L). The black solid line is the Ordinary Deming regression line, and the red dashed lines are the 95% PI. The blue dotted line is the identity line (y = x). The red triangles are the SRM samples which are identified in the plots for the 42-sample set.

Figure S9. Assessment of commutability using the CLSI 95% PI approach for (A and B) Roche assay and (C and D) Roche (Liège) assay using both the 50- and 42-single donor sets. The black circles (open and filled) are the single-donor samples. The black filled circles represent the single-donor samples with high 25(OH)D_2_ concentrations (>30 nmol/L). The black solid line is the Ordinary Deming regression line, and the red dashed lines are the 95% PI. The blue dotted line is the identity line (y = x). The red triangles are the SRM samples which are identified in the plots for the 42-sample set.

Figure S10. Assessment of commutability using the CLSI 95% PI approach for (A and B) Siemens ADVIA Centaur XP assay and (C and D) Siemens ADVIA Centaur XP (Liège) assay using both the 50- and 42-single donor sets. The black circles (open and filled) are the single-donor samples. The black filled circles represent the single-donor samples with high 25(OH)D_2_ concentrations (>30 nmol/L). The black solid line is the Ordinary Deming regression line, and the red dashed lines are the 95% PI. The blue dotted line is the identity line (y = x). The red triangles are the SRM samples which are identified in the plots for the 42-sample set.

Figure S11. Assessment of commutability using the CLSI 95% PI approach for (A and B) Siemens Atellica IM assay and (C and D) Siemens Dimension assay using both the 50- and 42-single donor sets. The black circles (open and filled) are the single-donor samples. The black filled circles represent the single-donor samples with high 25(OH)D_2_ concentrations (>30 nmol/L). The black solid line is the Ordinary Deming regression line, and the red dashed lines are the 95% PI. The blue dotted line is the identity line (y = x). The red triangles are the SRM samples which are identified in the plots for the 42-sample set.

Figure S12. Assessment of commutability using the CLSI 95% PI approach for (A and B) Snibe (Liège) assay and (C and D) Tosoh assay using both the 50- and 42-single donor sets. The black circles (open and filled) are the single-donor samples. The black filled circles represent the single-donor samples with high 25(OH)D_2_ concentrations (>30 nmol/L). The black solid line is the Ordinary Deming regression line, and the red dashed lines are the 95% PI. The blue dotted line is the identity line (y = x). The red triangles are the SRM samples which are identified in the plots for the 42-sample set.

Figure S13. Assessment of commutability using the CLSI 95% PI approach for (A and B) Chromsystems 1 LC-MS/MS assay and (C and D) Chromsystems 2 LC-MS/MS assay using both the 50- and 42-single donor sets. The black circles (open and filled) are the single-donor samples. The black filled circles represent the single-donor samples with high 25(OH)D_2_ concentrations (>30 nmol/L). The black solid line is the Ordinary Deming regression line, and the red dashed lines are the 95% PI. The blue dotted line is the identity line (y = x). The red triangles are the SRM samples which are identified in the plots for the 42-sample set.

Figure S14. Assessment of commutability using the CLSI 95% PI approach for (A and B) University College Cork LC-MS/MS assay and (C and D) Health Canada LC-MS/MS assay using both the 50- and 42-single donor sets. The black circles (open and filled) are the single-donor samples. The black filled circles represent the single-donor samples with high 25(OH)D_2_ concentrations (>30 nmol/L). The black solid line is the OrdinaryDeming regression line, and the red dashed lines are the 95% PI. The blue dotted line is the identity line (y = x). The red triangles are the SRM samples which are identified in the plots for the 42-sample set.

Figure S15. Assessment of commutability using the CLSI 95% PI approach for (A and B) Imperial College Healthcare Trust (ICHT) LC-MS/MS assay and (C and D) University of Liege LC-MS/MS assay using both the 50- and 42-single donor sets. The black circles (open and filled) are the single-donor samples. The black filled circles represent the single-donor samples with high 25(OH)D_2_ concentrations (>30 nmol/L). The black solid line is the Ordinary Deming regression line, and the red dashed lines are the 95% PI. The blue dotted line is the identity line (y = x). The red triangles are the SRM samples which are identified in the plots for the 42-sample set.

Figure S16. Assessment of commutability using the CLSI 95% PI approach for (A and B) Quest LC-MS/MS assay and (C and D) University of Washington LC-MS/MS assay using both the 50- and 42-single donor sets. The black circles (open and filled) are the single-donor samples. The black filled circles represent the single-donor samples with high 25(OH)D_2_ concentrations . The black solid line is the Ordinary Deming regression line, and the red dashed lines are the 95% PI. The blue dotted line is the identity line (y = x). The red triangles are the SRM samples which are identified in the plots for the 42-sample set.

Figure S17. Assessment of commutability using the CLSI 8.8% pre-set limit approach for the (A) Abbott Alinity (Liège) assay, (B) Abbott Alinity (ICHT) assay, (C) Abbott ARCHITECT assay, and (D) Affimedix assay. The black circles (open and filled) are the single-donor samples. The black filled circles represent the single-donor samples with high 25(OH)D_2_ concentrations (>30 nmol/L). The black solid line is the Ordinary Deming regression line, and the red dotted lines are the 8.8% pre-set limits. The red triangles are the SRM samples.

Figure S18. Assessment of commutability using the CLSI 8.8% pre-set limit approach for the (A) Beckman Coulter (Liège) assay, (B) bioMérieux assay, (C) bioMérieux (Liège) assay, and (D) Diazyme assay. The black circles (open and filled) are the single-donor samples. The black filled circles represent the single-donor samples with high 25(OH)D_2_ concentrations (>30 nmol/L). The black solid line is the Ordinary Deming regression line, and the red dotted lines are the 8.8% pre-set limits. The red triangles are the SRM samples.

Figure S19. Assessment of commutability using the CLSI 8.8% pre-set limit approach for the (A) DiaSorin (Liège) assay, (B) DiaSorin (ICHT) assay, (C) Fujirebio assay, and (D) Fujirebio (Liège) assay. The black circles (open and filled) are the single-donor samples. The black filled circles represent the single-donor samples with high 25(OH)D_2_ concentrations (>30 nmol/L). The black solid line is the Ordinary Deming regression line, and the red dotted lines are the 8.8% pre-set limits. The red triangles are the SRM samples.

Figure S20. Assessment of commutability using the CLSI 8.8% pre-set limit approach for the (A) IDS-EIA assay, (B) PerkinElmer assay, (C) IDS-iSYS assay, and (D)IDS-iSYS (Liège) assay. The black circles (open and filled) are the single-donor samples. The black filled circles represent the single-donor samples with high 25(OH)D_2_ concentrations (>30 nmol/L). The black solid line is the Ordinary Deming regression line, and the red dotted lines are the 8.8% pre-set limits. The red triangles are the SRM samples.

Figure S21. Assessment of commutability using the CLSI 8.8% pre-set limit approach for the (A) Roche assay, (B) Roche (Liège) assay, (C) Siemens ADVIA Centaur XP assay, and (D) Siemens ADVIA Centaur (Liège) assay. The black circles (open and filled) are the single-donor samples. The black filled circles represent the single-donor samples with high 25(OH)D_2_ concentrations (>30 nmol/L). The black solid line is the Ordinary Deming regression line, and the red dotted lines are the 8.8% pre-set limits. The red triangles are the SRM samples.

Figure S22. Assessment of commutability using the CLSI 8.8% pre-set limit approach for the (A) Siemens Atellica IM assay, (B) Siemens Dimension assay, (C) Snibe (Liège) assay, and (D) Tosoh assay. The black circles (open and filled) are the single-donor samples. The black filled circles represent the single-donor samples with high 25(OH)D_2_ concentrations (>30 nmol/L). The black solid line is the Ordinary Deming regression line, and the red dotted lines are the 8.8% pre-set limits. The red triangles are the SRM samples.

Figure S23. Assessment of commutability using the CLSI 8.8% pre-set limit approach for the (A) CDC LC-MS/MS assay, (B) University College Cork LC-MS/MS assay, (C) Chromsystems 1 LC-MS/MS assay, and (D) Chromsystems 2 LC-MS/MS assay. The black circles (open and filled) are the single-donor samples. The black filled circles represent the single-donor samples with high 25(OH)D_2_ concentrations (>30 nmol/L). The black solid line is the Ordinary Deming regression line, and the red dotted lines are the 8.8% pre-set limits. The red triangles are the SRM samples.

Figure S24. Assessment of commutability using the CLSI 8.8% pre-set limit approach for the (A) Imperial College Healthcare Trust (ICHT) LC-MS/MS assay, (B) University of Liège LC-MS/MS assay, (C) Quest LC-MS/MS assay, and (D) University of Washington LC-MS/MS assay. The black circles (open and filled) are the single-donor samples. The black filled circles represent the single-donor samples with high 25(OH)D_2_ concentrations (>30 nmol/L). The black solid line is the Ordinary Deming regression line, and the red dotted lines are the 8.8% pre-set limits. The red triangles are the SRM samples.

Figure S25. Assessment of commutability using the IFCC approach for LC-MS/MS assays: (A) Chromsystem 2, (B) Imperial College Healthcare Trust (ICHT), (C) University of Liège, and (D) Quest. Red dashed lines are ± the commutability criterion (C) of 10%. The error bars are the expanded uncertainty, *U*(d_RM_), which is a 95% confidence interval for the d_RM_ values.

Figure S26. Assessment of commutability using the IFCC approach for: (A) Abbott Alinity (Liège) assay, (B) Abbott Alinity (ICHT) assay, (C) Abbott ARCHITECT assay, and (D) bioMérieux (Liège). Red dashed lines are ± the commutability criterion (C) of 8.8%. The error bars are the expanded uncertainty, *U*(d_RM_), which is a 95% confidence interval for the d_RM_ values.

Figure S27. Assessment of commutability using the IFCC approach for: (A) DiaSorin (ICHT) assay, (B) Diazyme assay, (C) IDS-EIA assay, and (D) IDS-iSYS (Liège). Red dashed lines are ± the commutability criterion (C) of 8.8%. The error bars are the expanded uncertainty, *U*(d_RM_), which is a 95% confidence interval for the d_RM_ values.

Figure S28. Assessment of commutability using the IFCC approach for: (A) Fujirebio (Liège), (B) IDS-EIA assay, (C) Roche assay, and (D) Roche (Liège) assay. Red dashed lines are ± the commutability criterion (C) of 8.8%. The error bars are the expanded uncertainty, *U*(d_RM_), which is a 95% confidence interval for the d_RM_ values.

Figure S29. Assessment of commutability using the IFCC approach for: Siemens ADVIA (Liège), (B) Siemens Atellica assay, (C) Snibe (Liège) assay, and (D) Tosoh assay. Red dashed lines are ± the commutability criterion (C) of 8.8%. The error bars are the expanded uncertainty, *U*(d_RM_), which is a 95% confidence interval for the d_RM_ values.

Figure S30. Relationship of total 25(OH)D and VDBP for the four levels of SRM 1949. Error bars for both total 25(OH)D and VDBP concentrations are the uncertainty associated with the assigned values from the Certificate of Analysis for SRM 1949 and reported by Boggs et al. [42].

Figure S1.


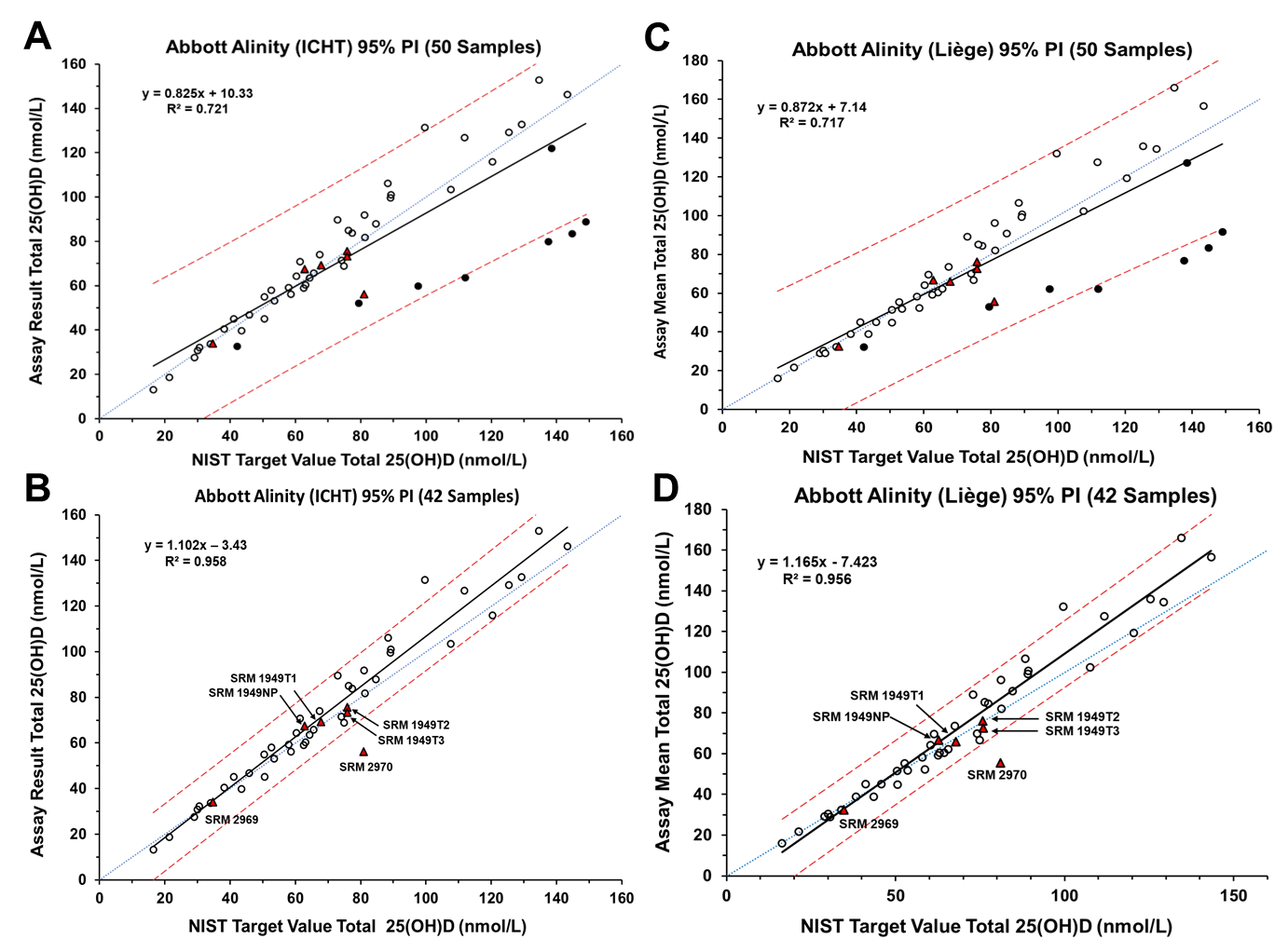


Figure S2.


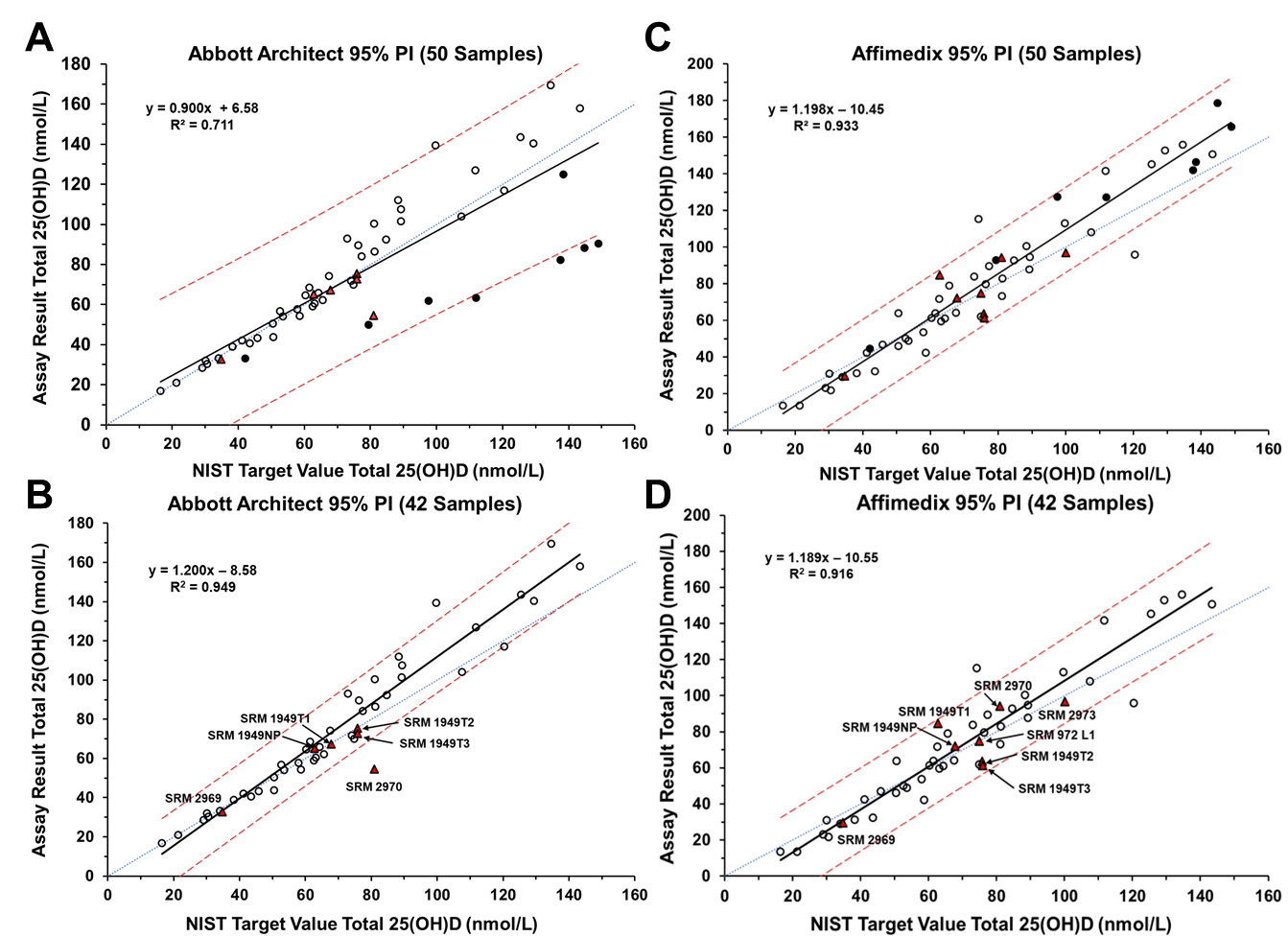


Figure S3.


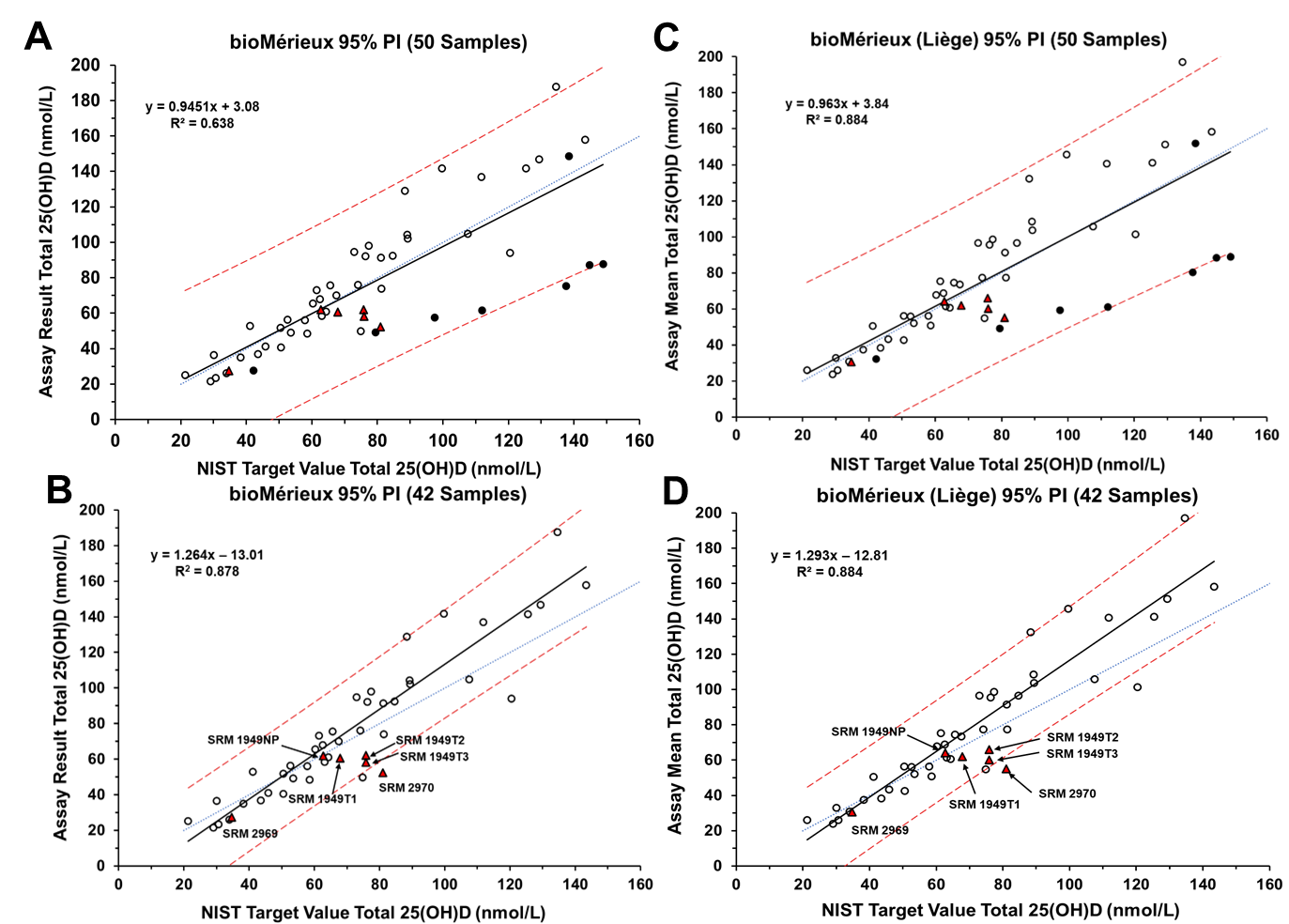


Figure S4.


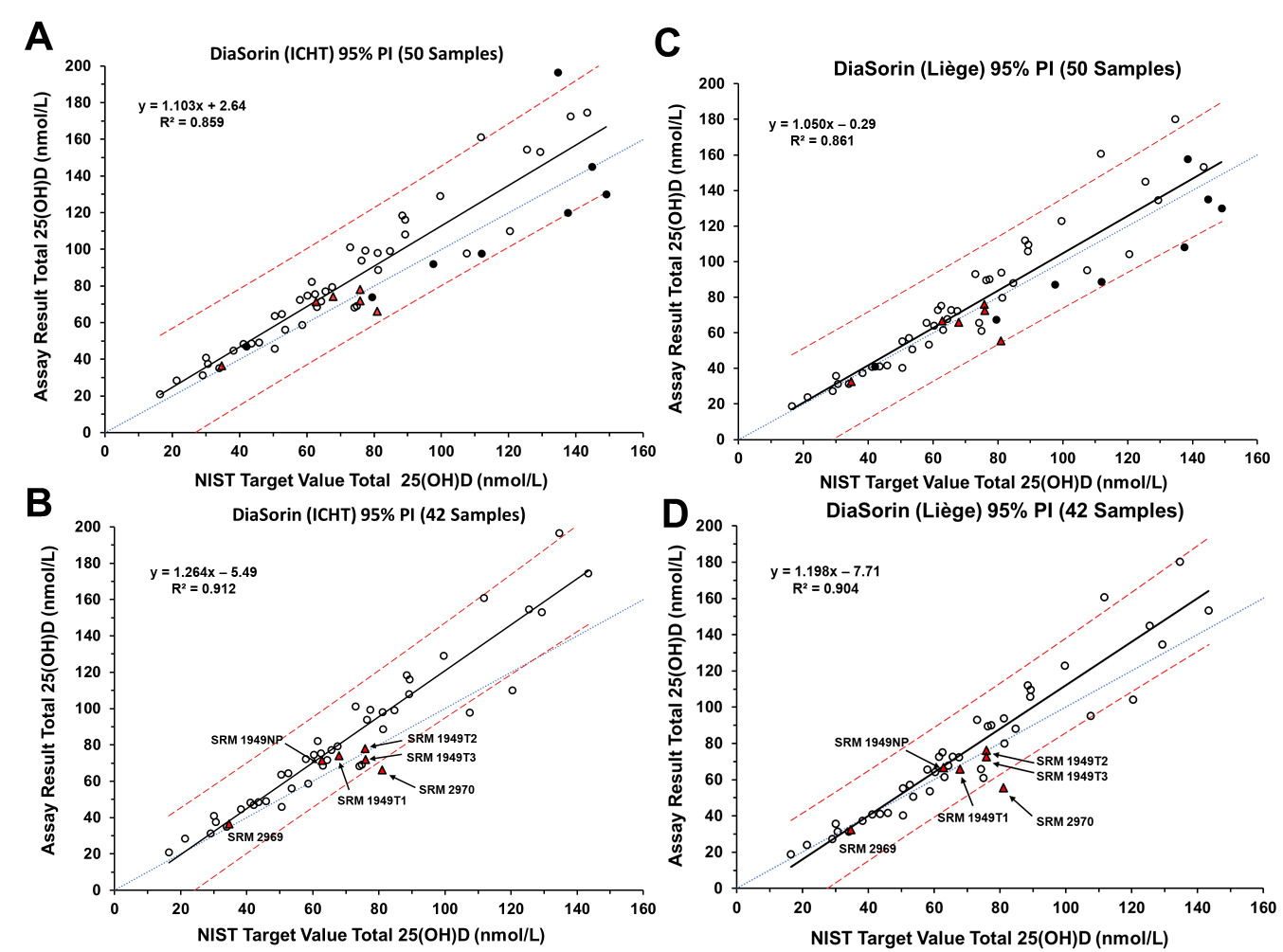


Figure S5.


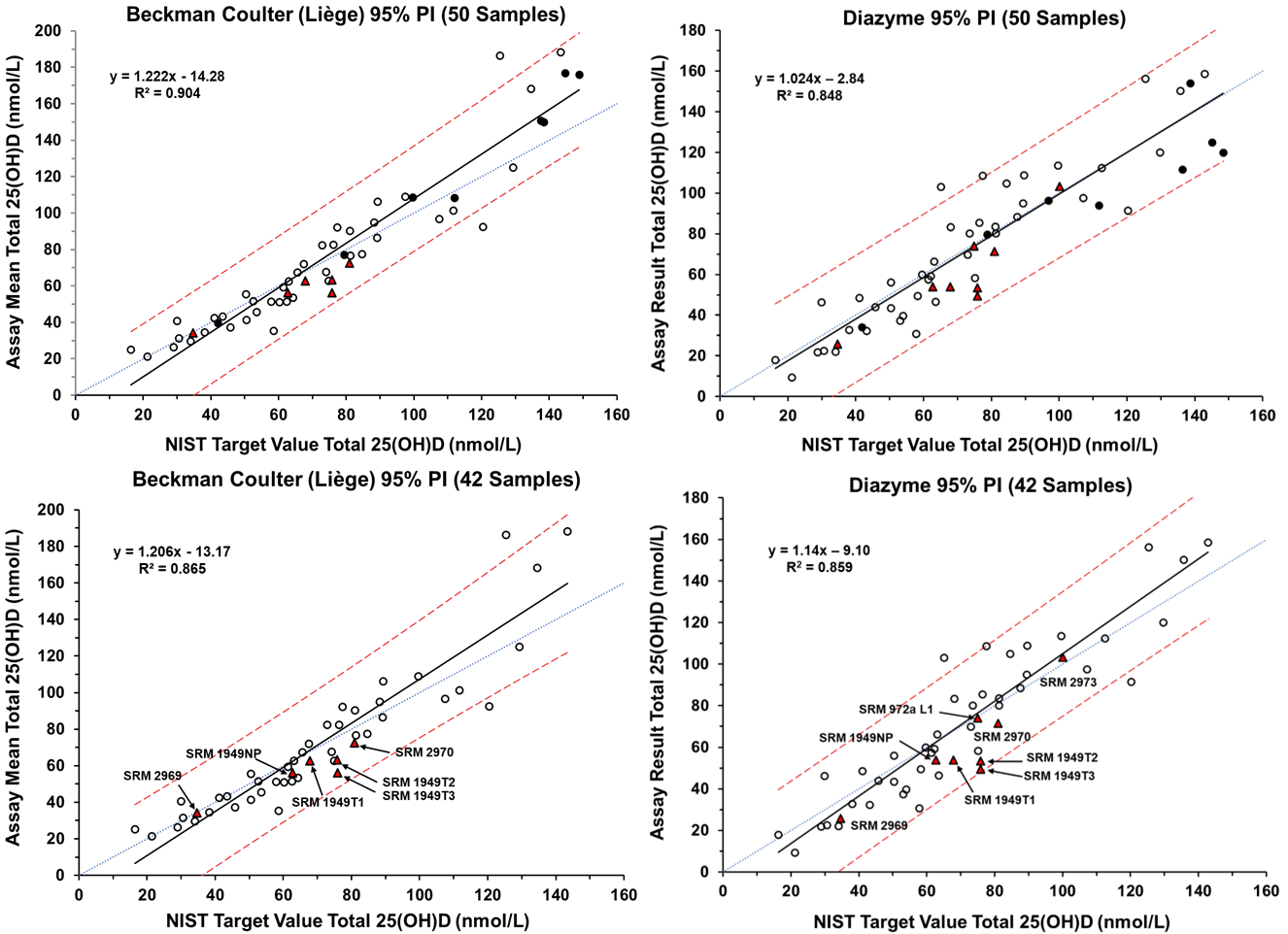


Figure S6.


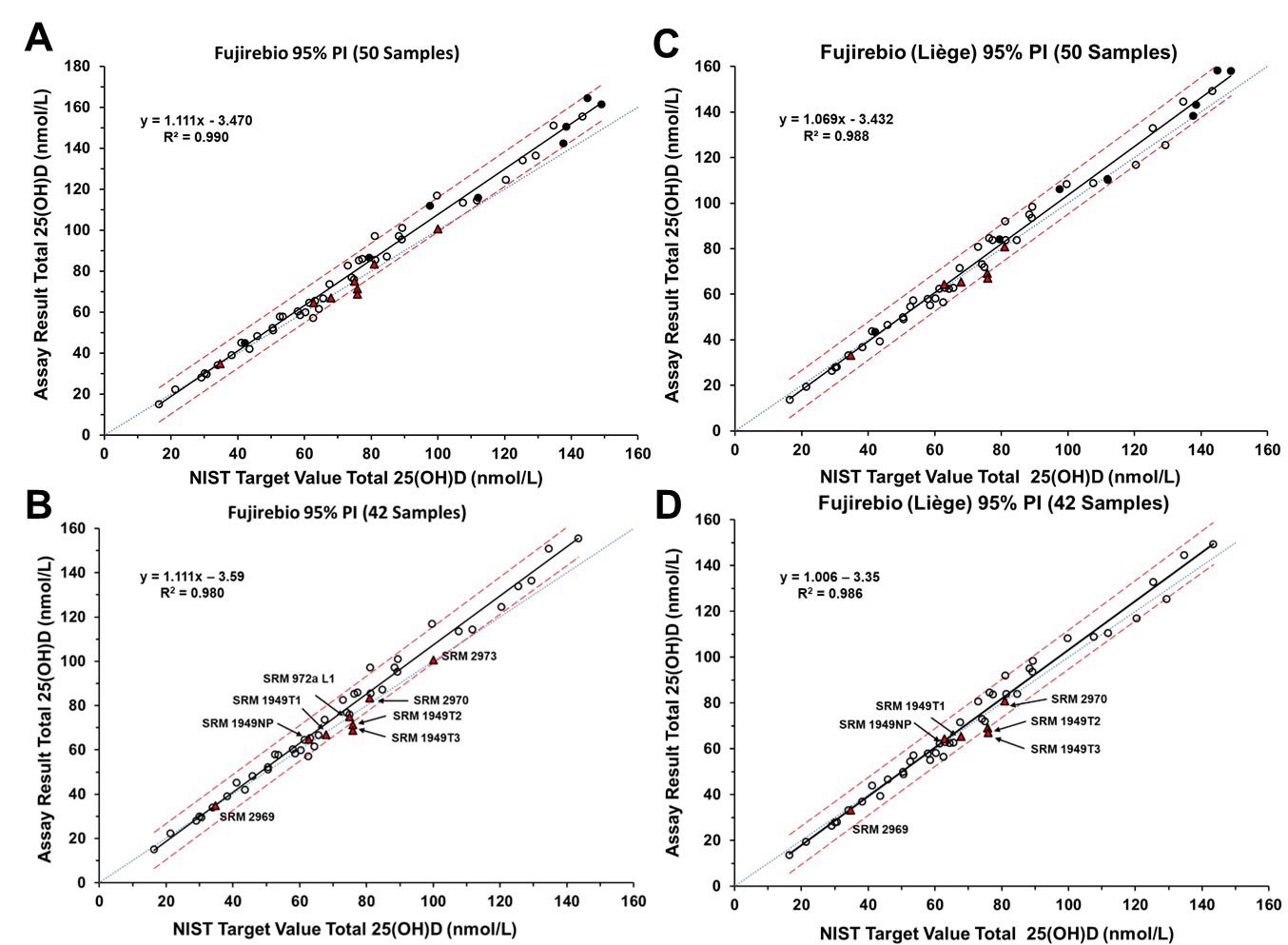


Figure S7.


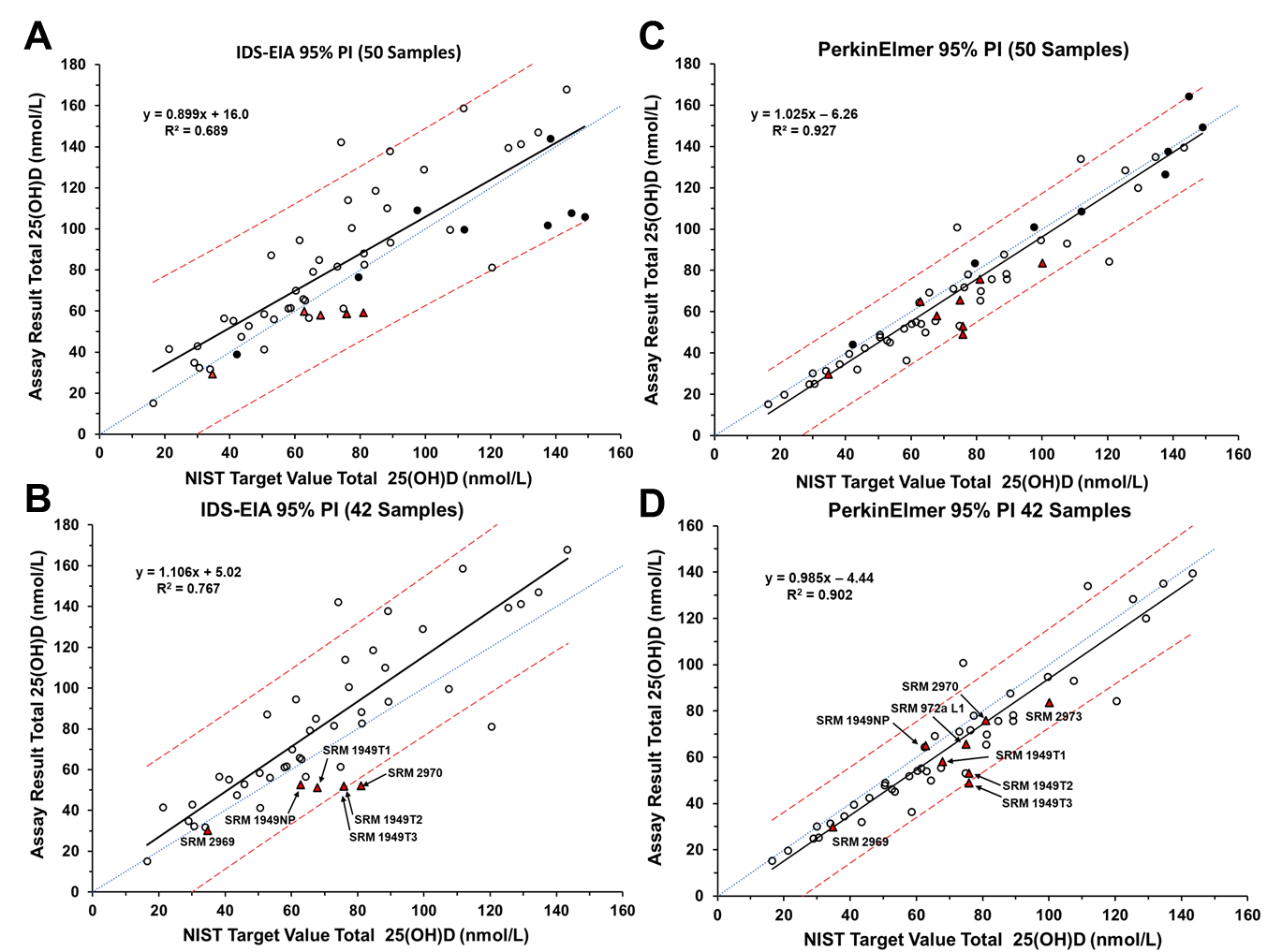


Figure S8.


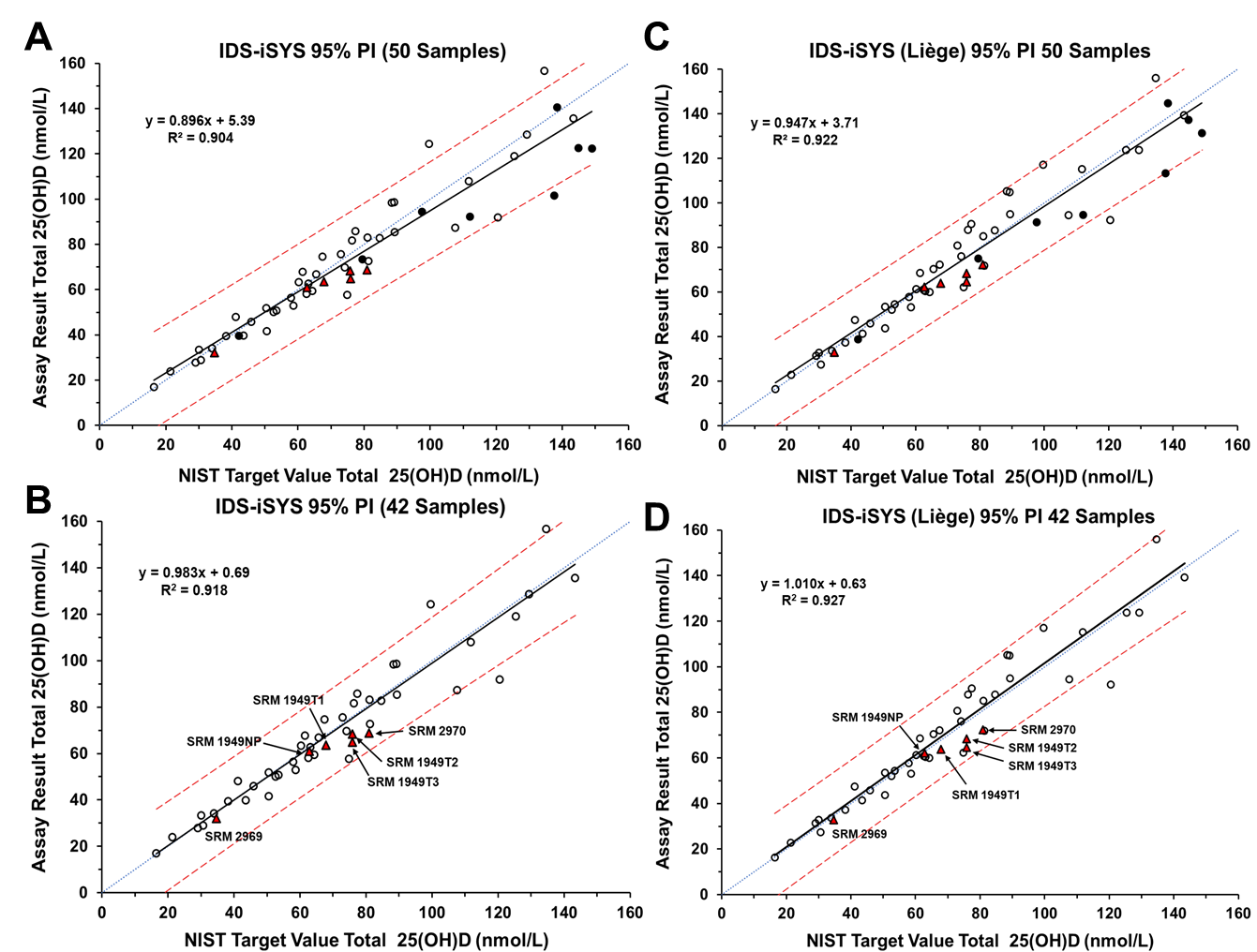


Figure S9.


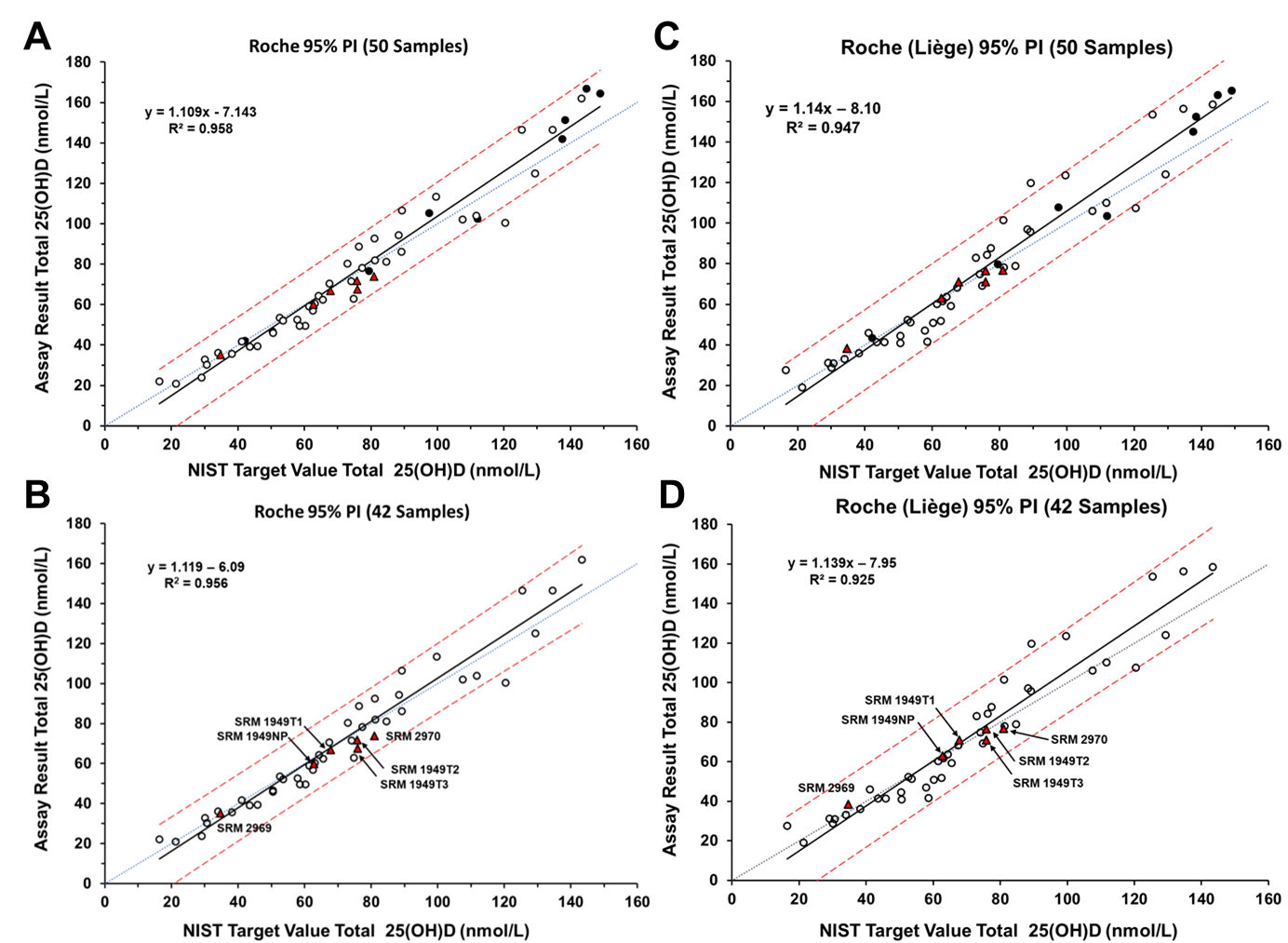


Figure S10.


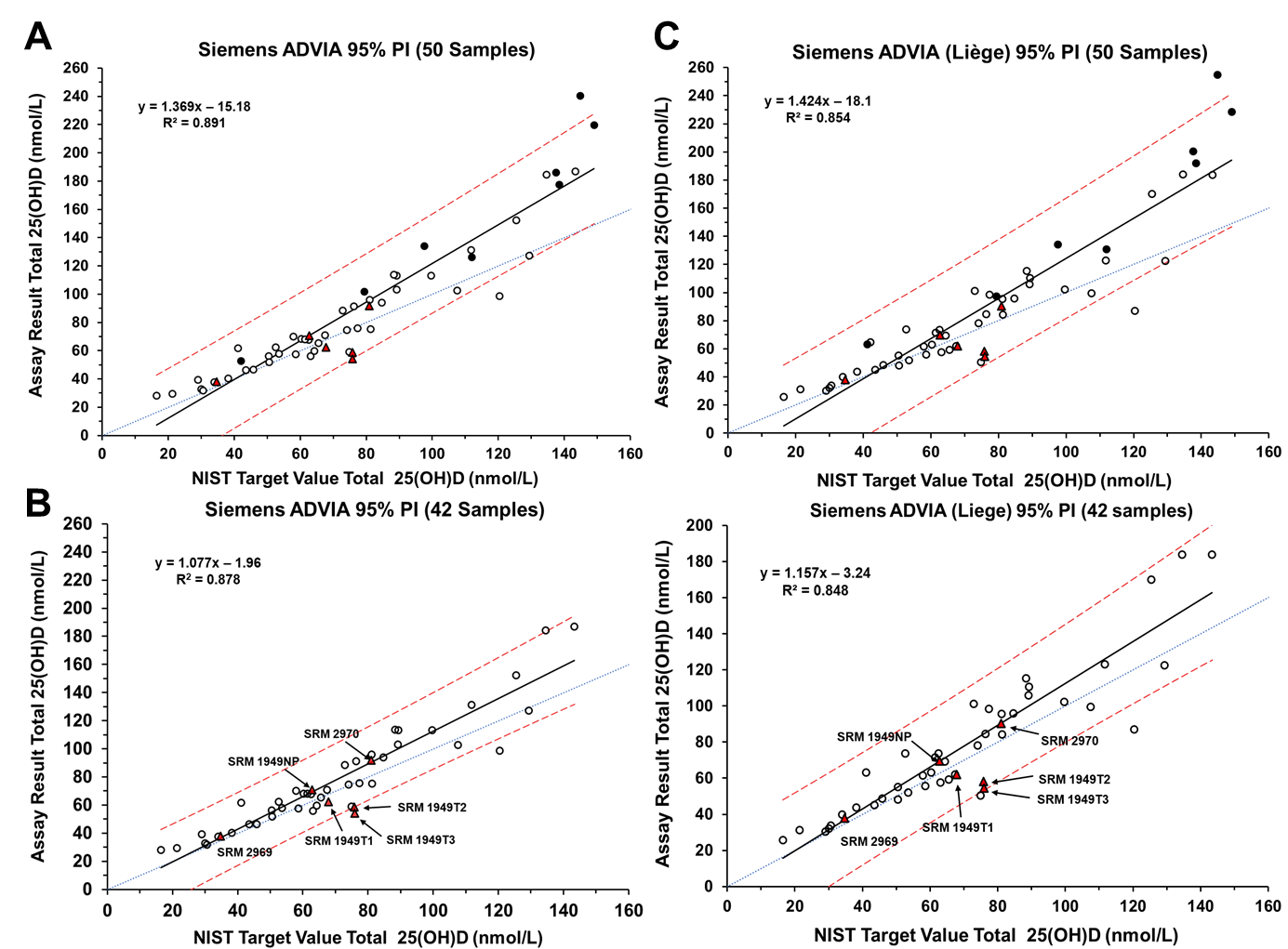


Figure S11.


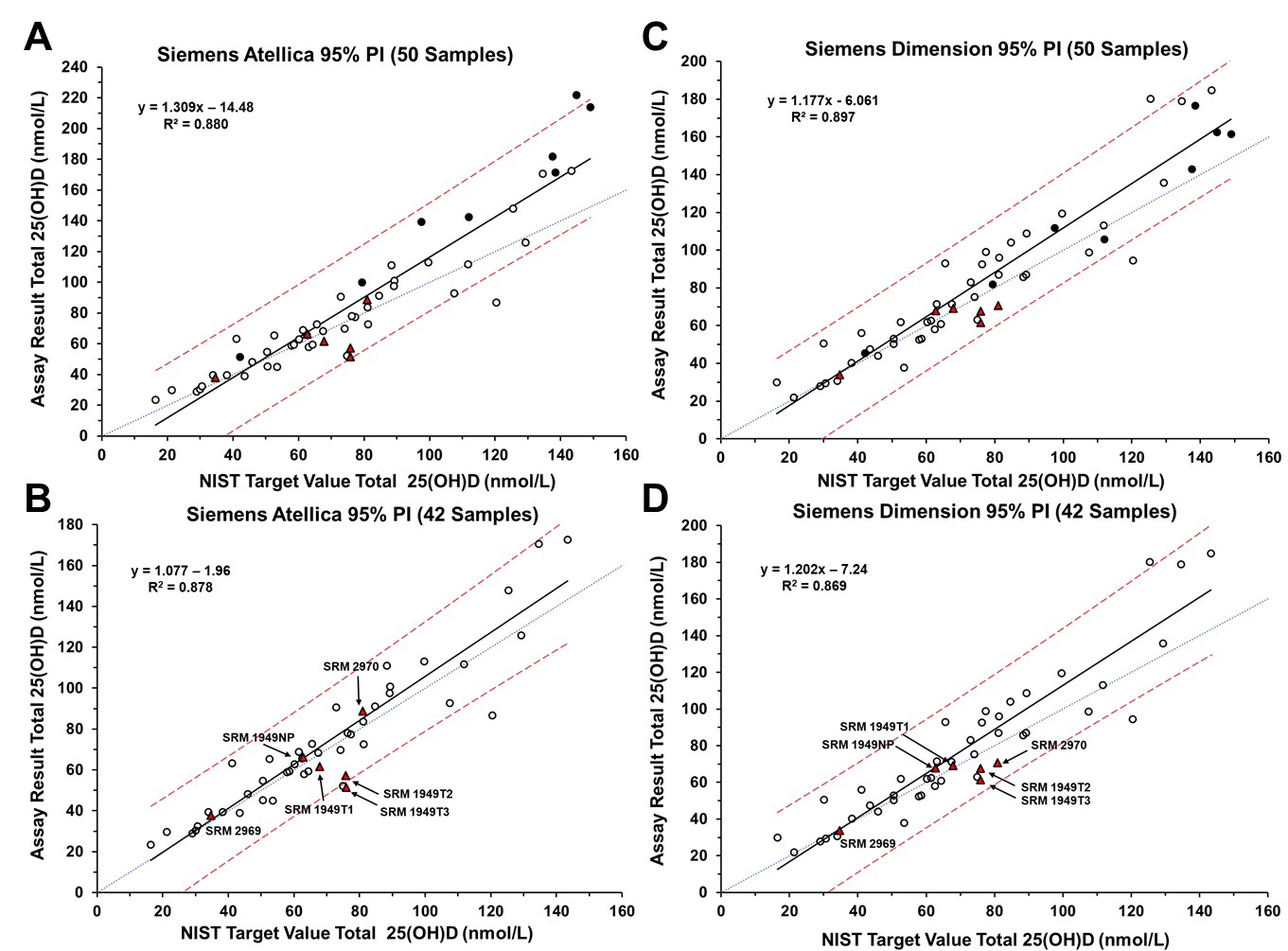


Figure S12.


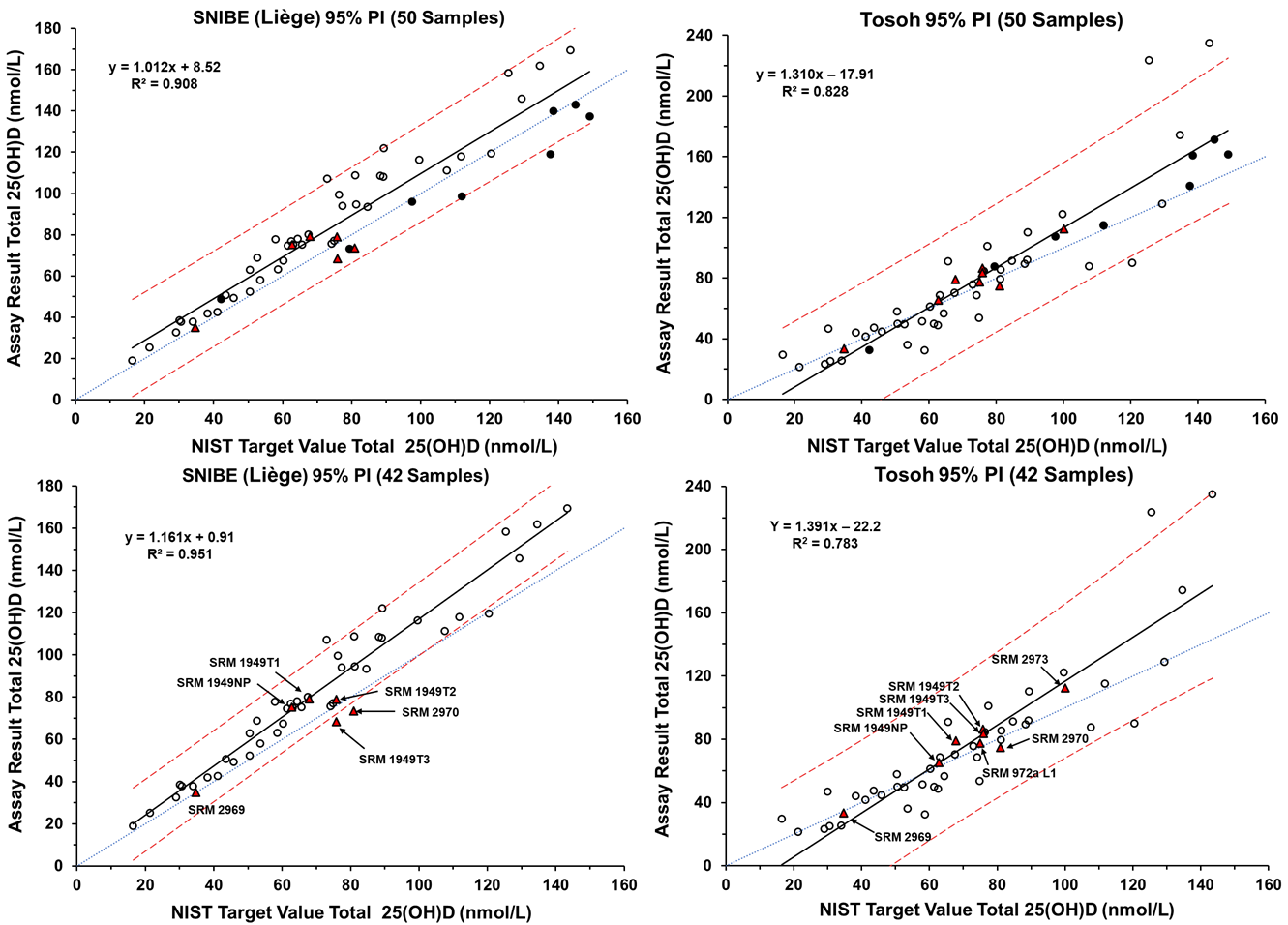


Figure S13.


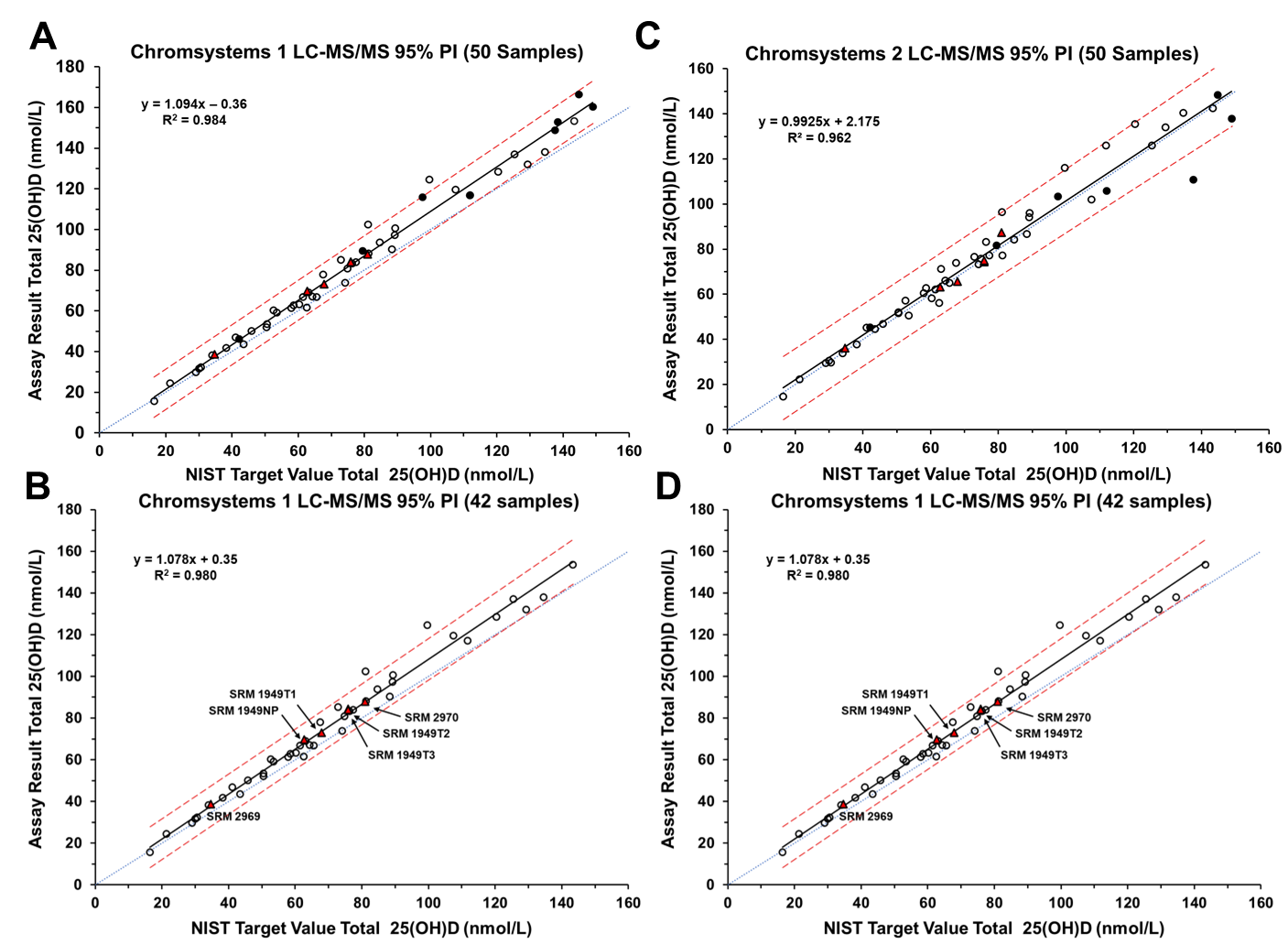


Figure S14.


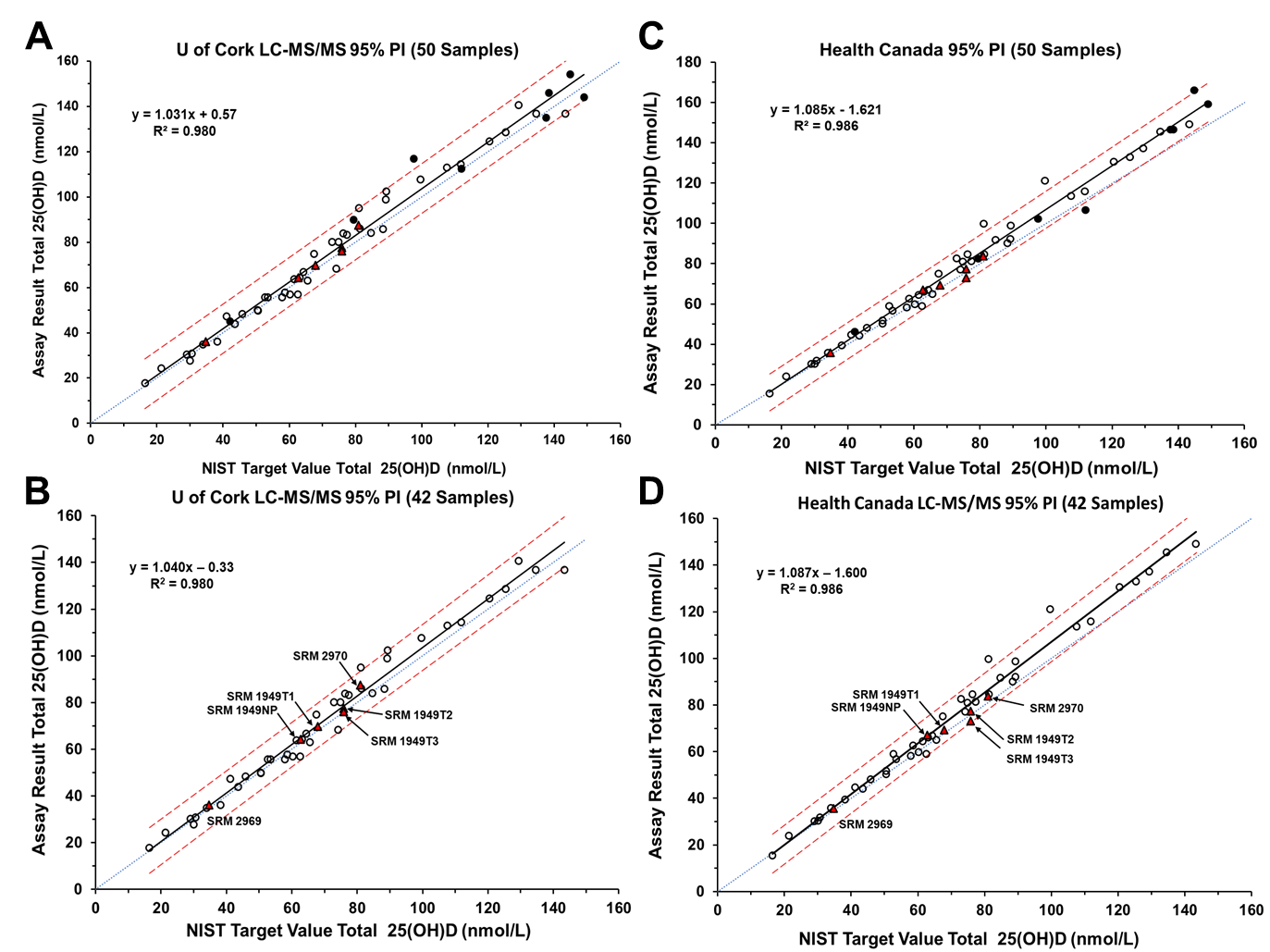


Figure S15.


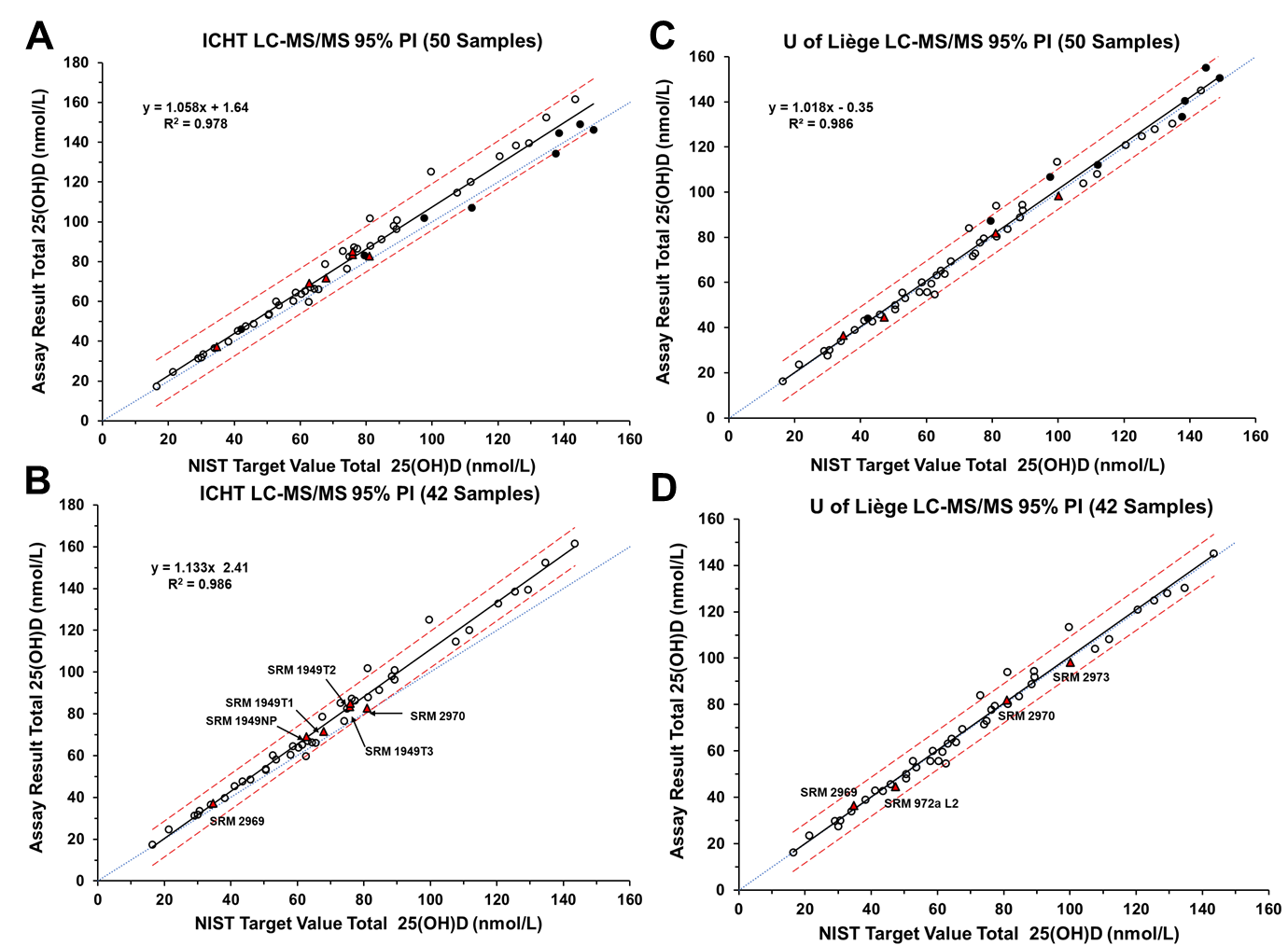


Figure S16.


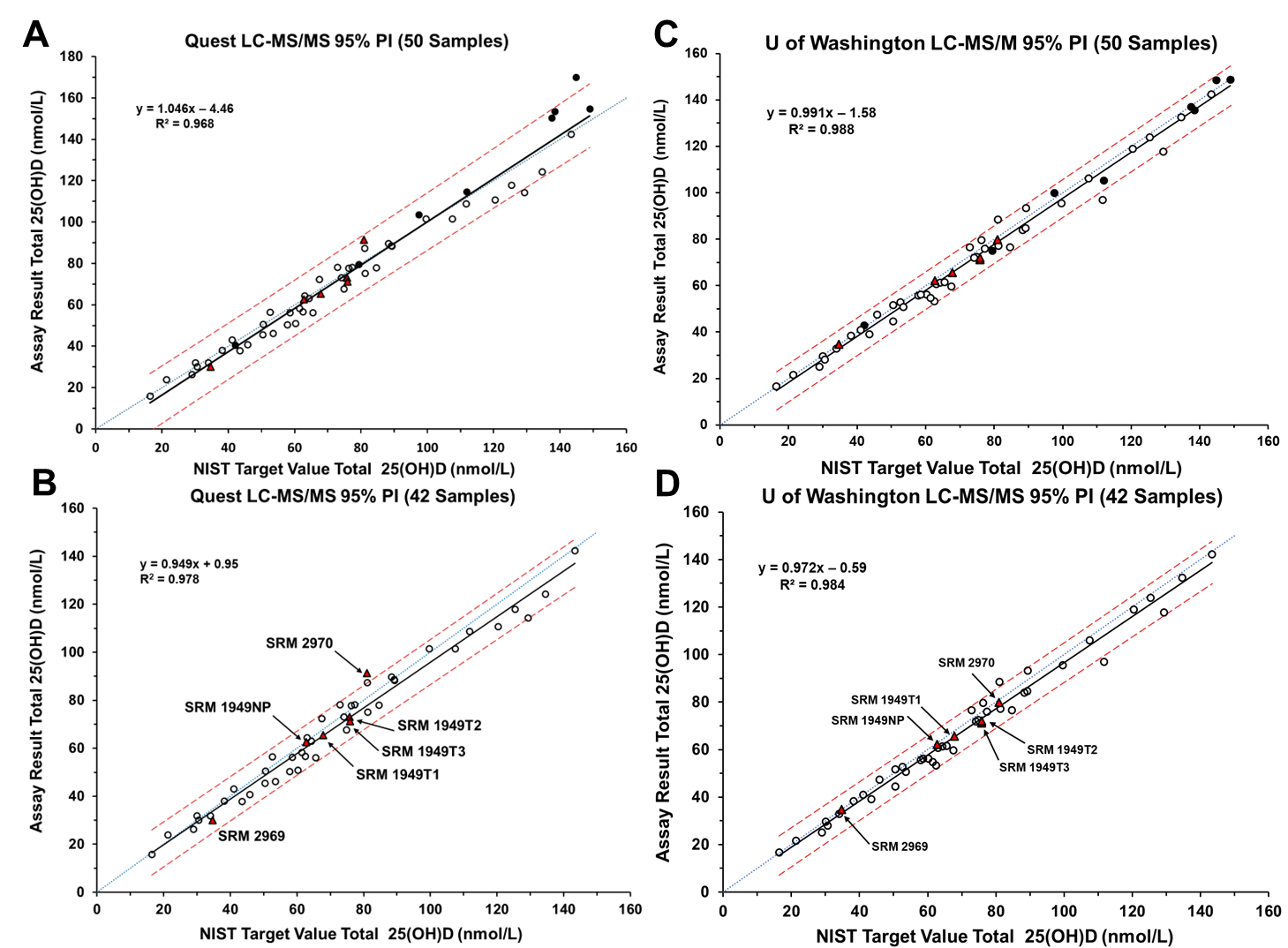
Figure S17.


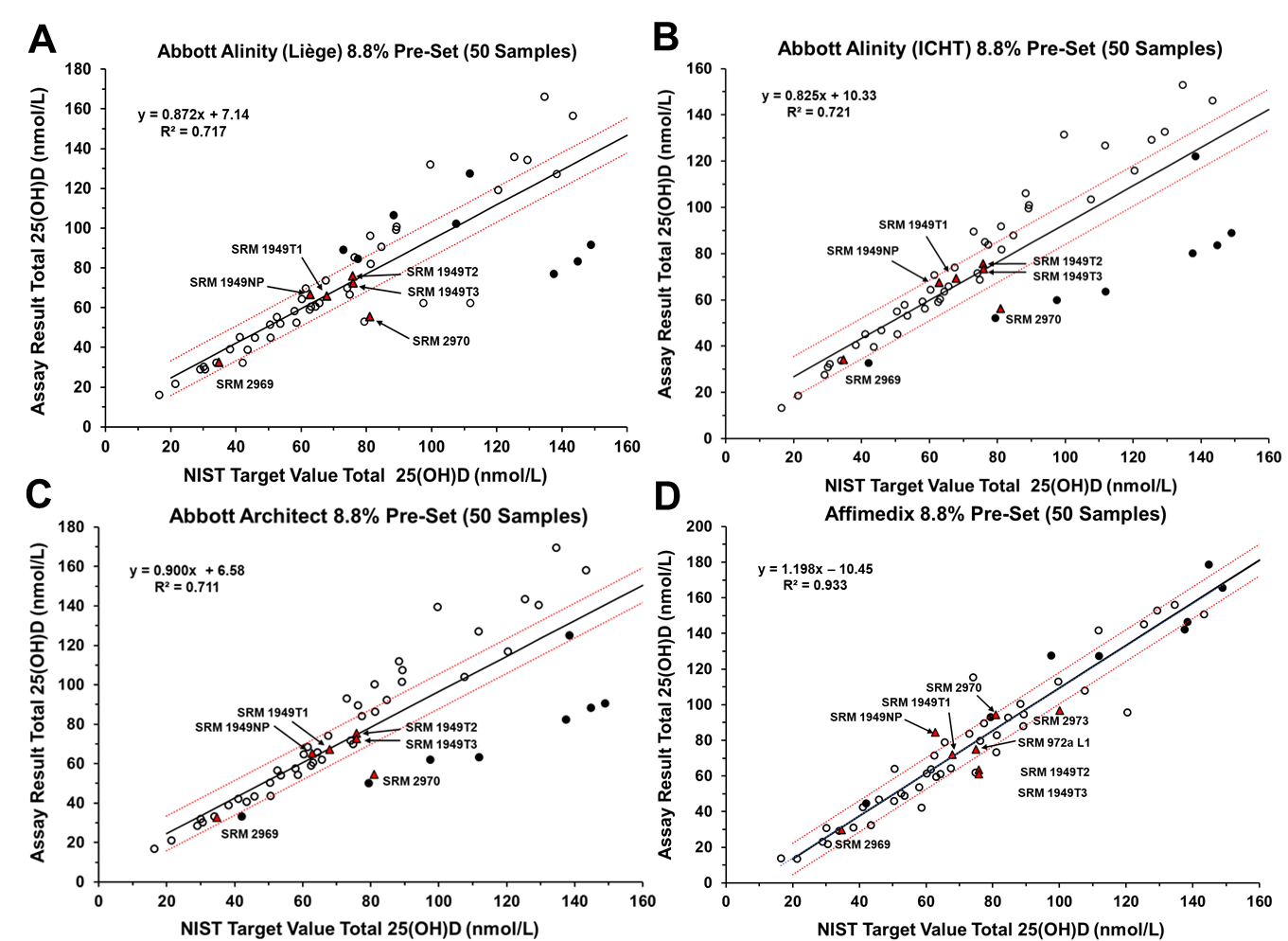


Figure S18.


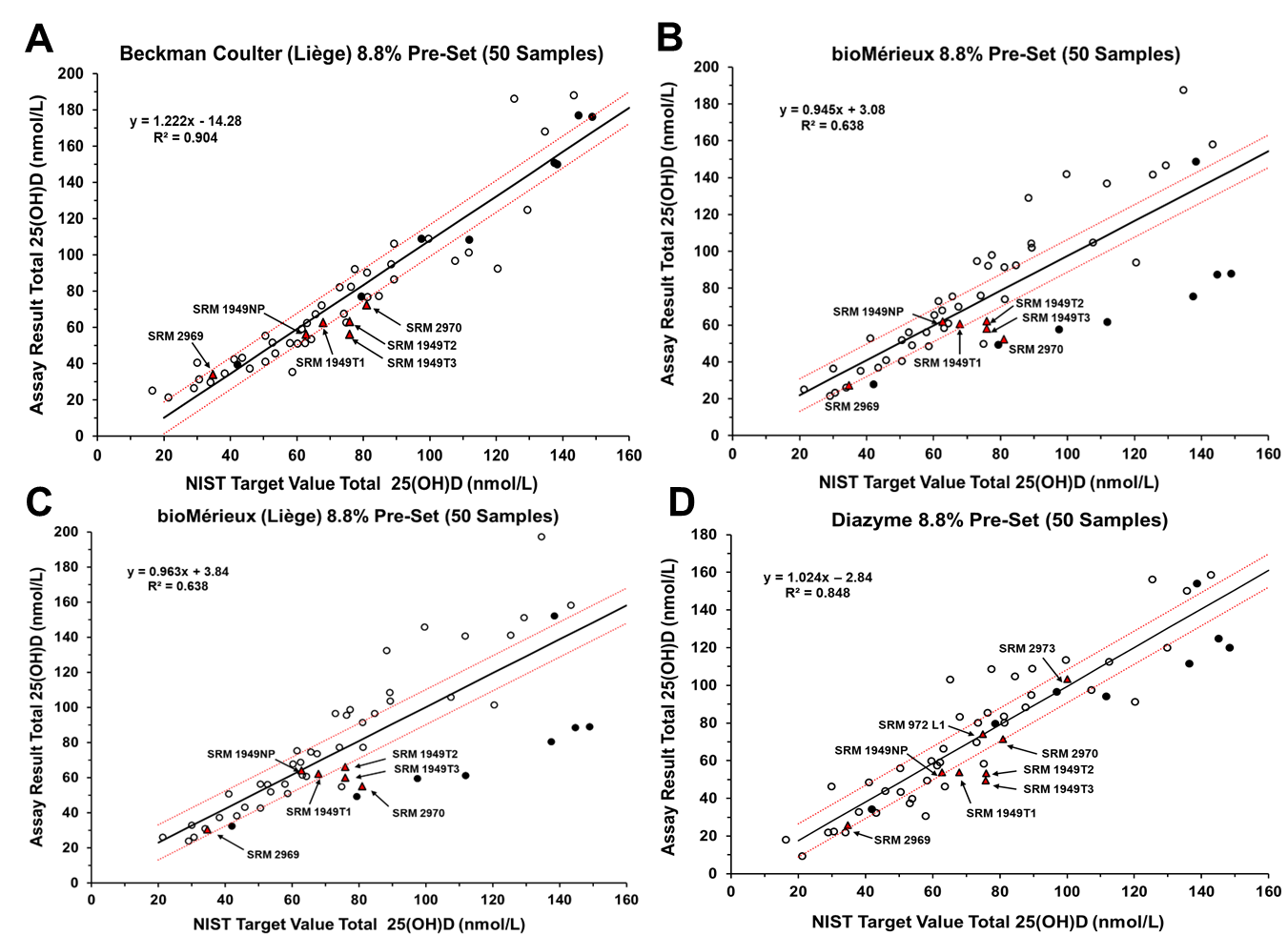


Figure S19.


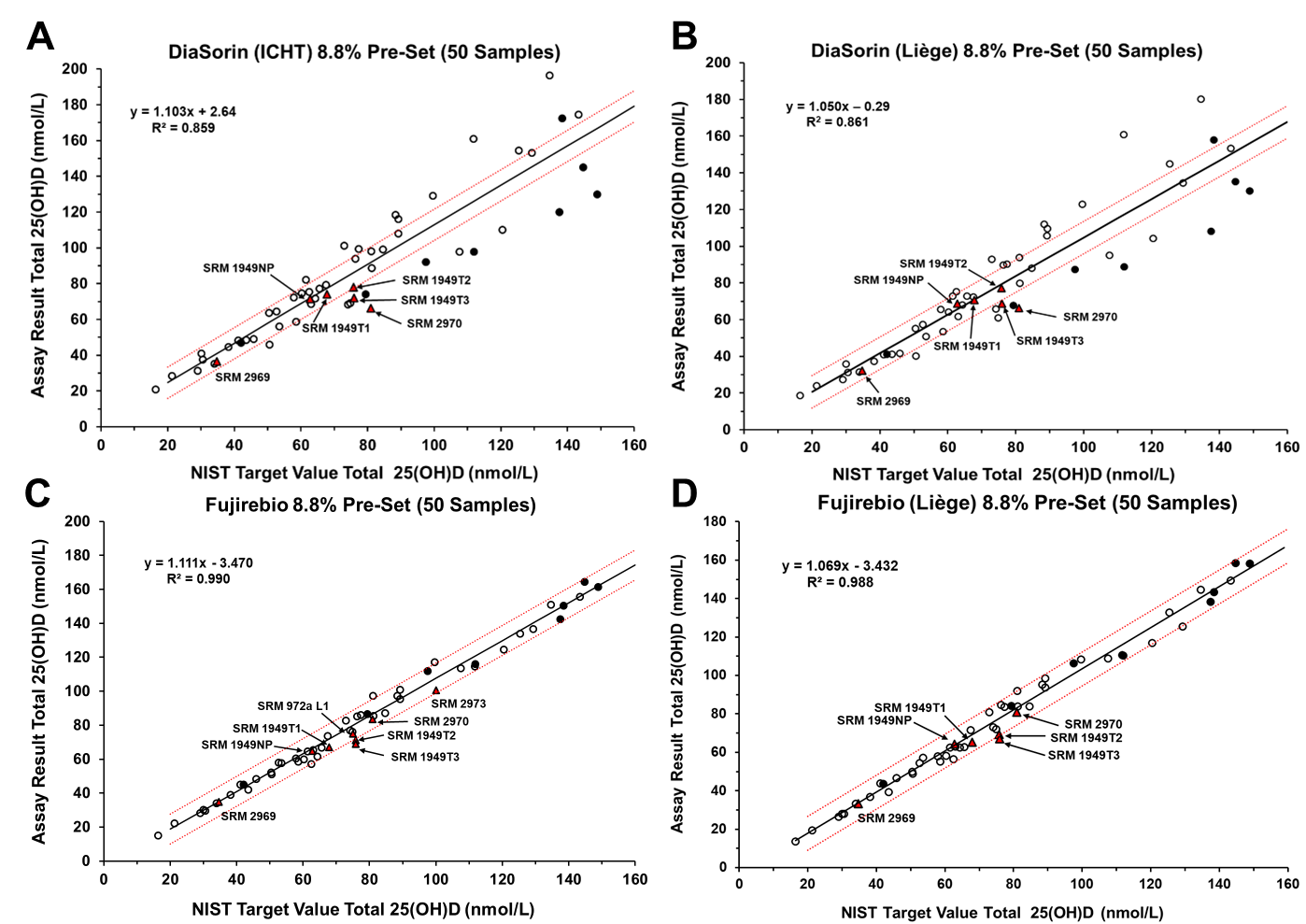


Figure S20.


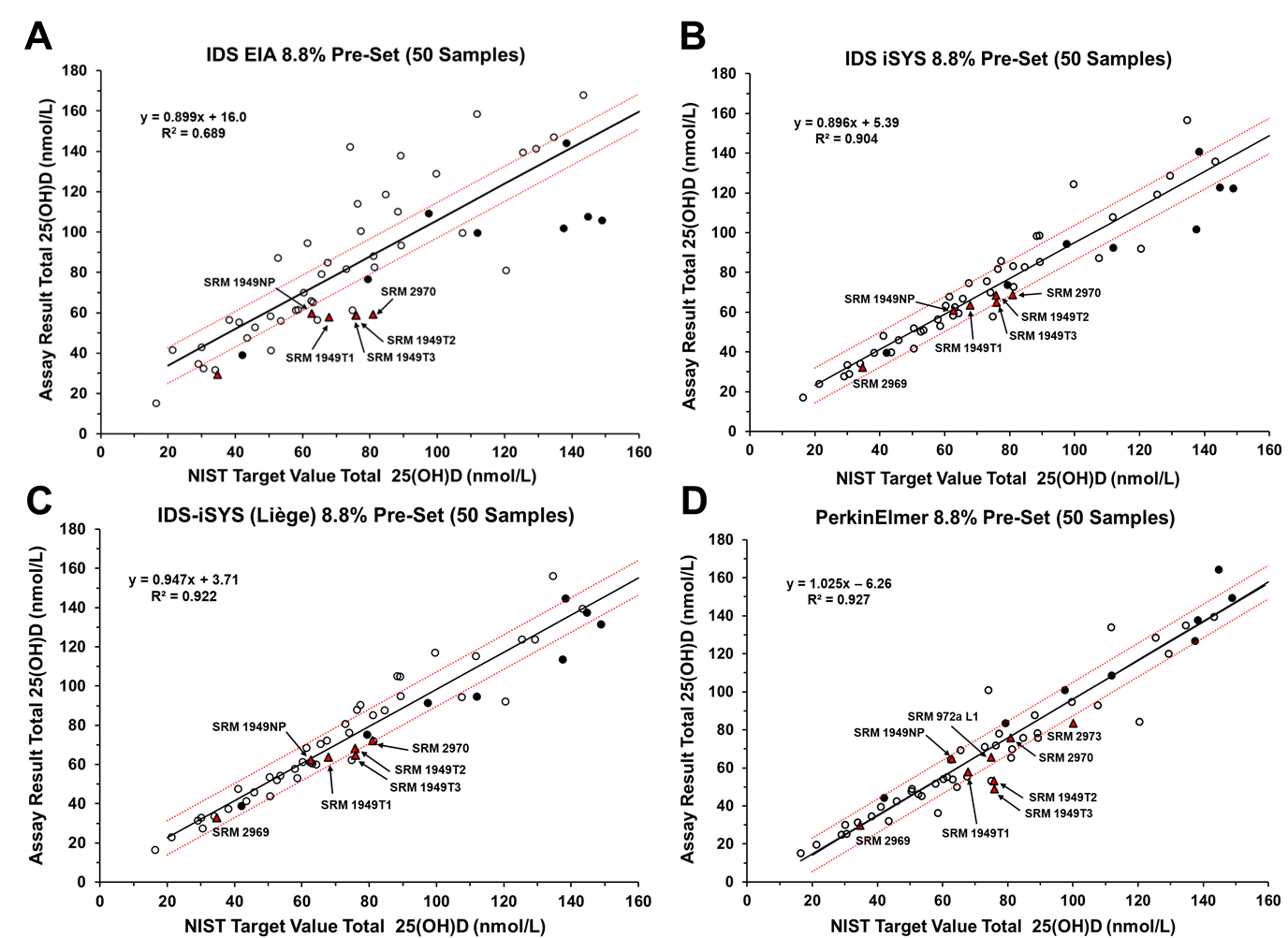


Figure S21.


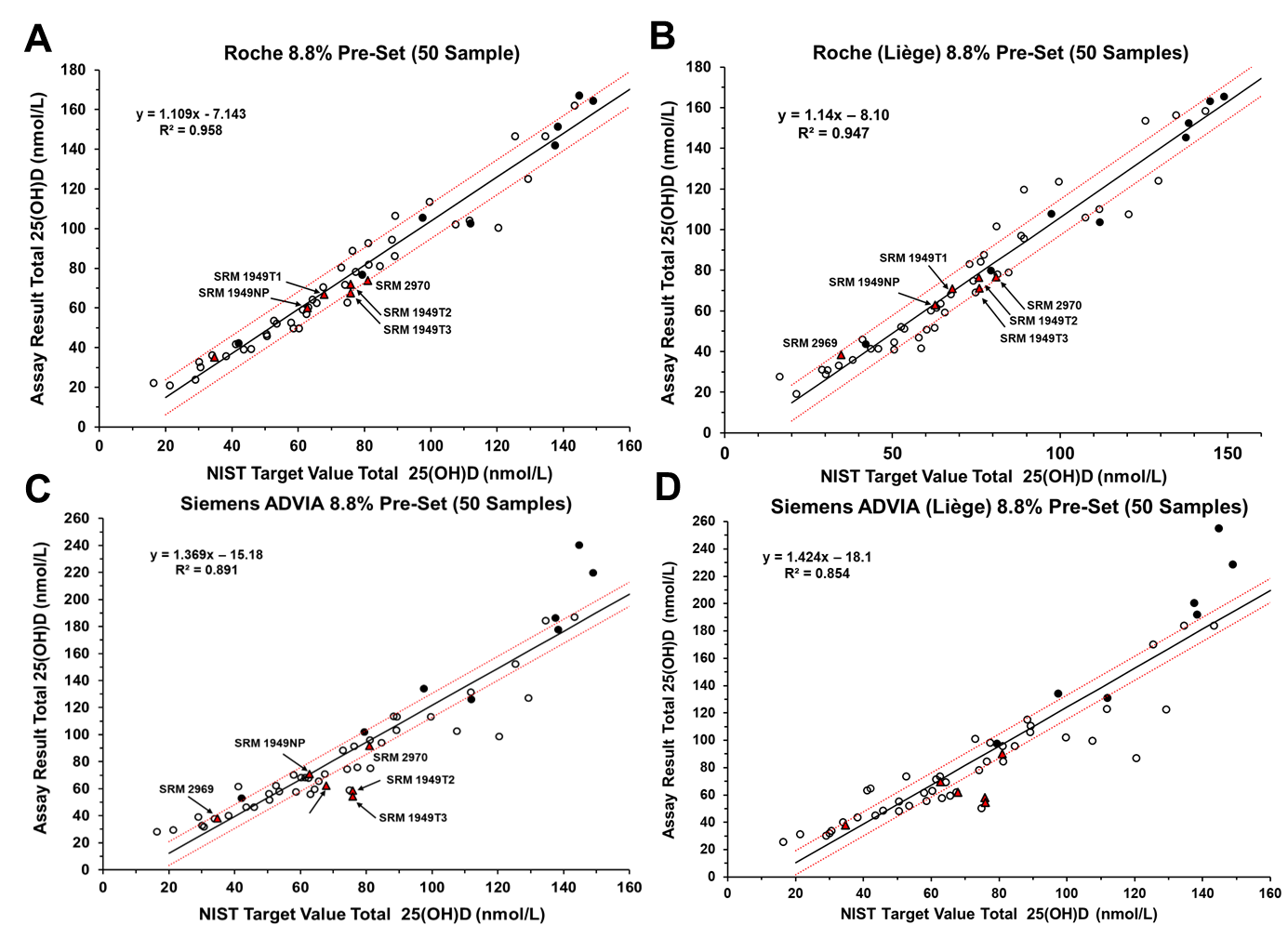


Figure S22.


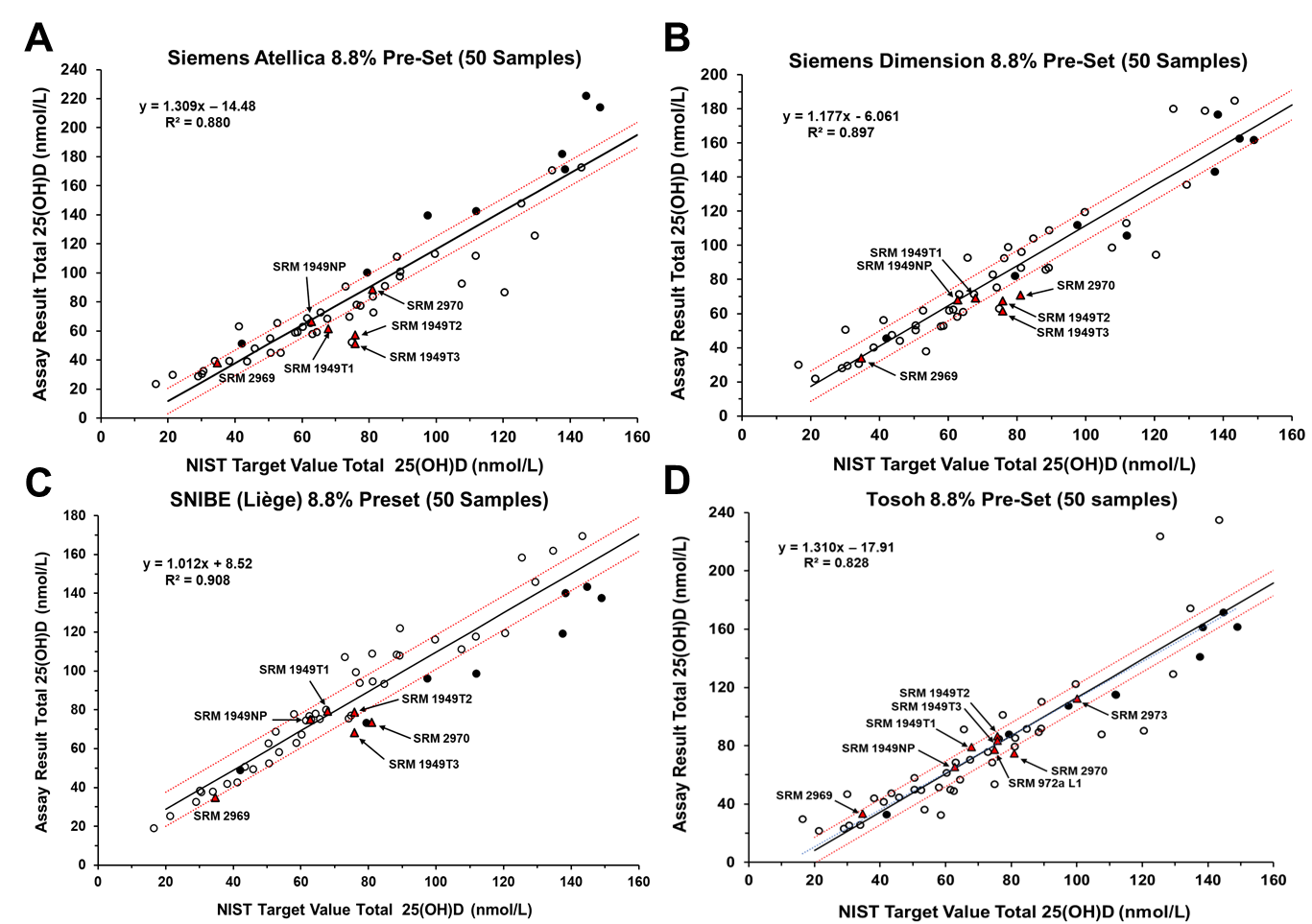


Figure S23.


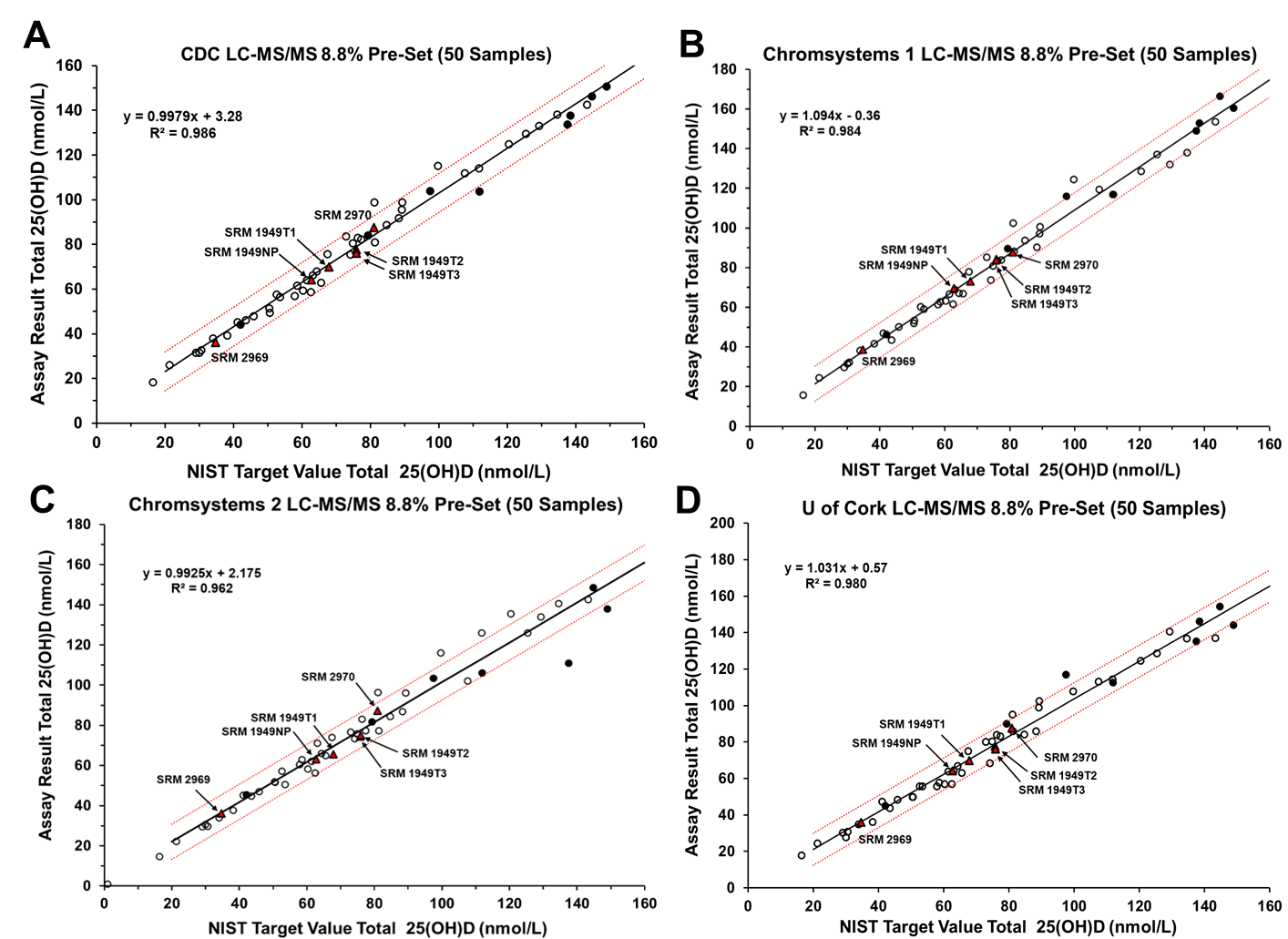


Figure S24.


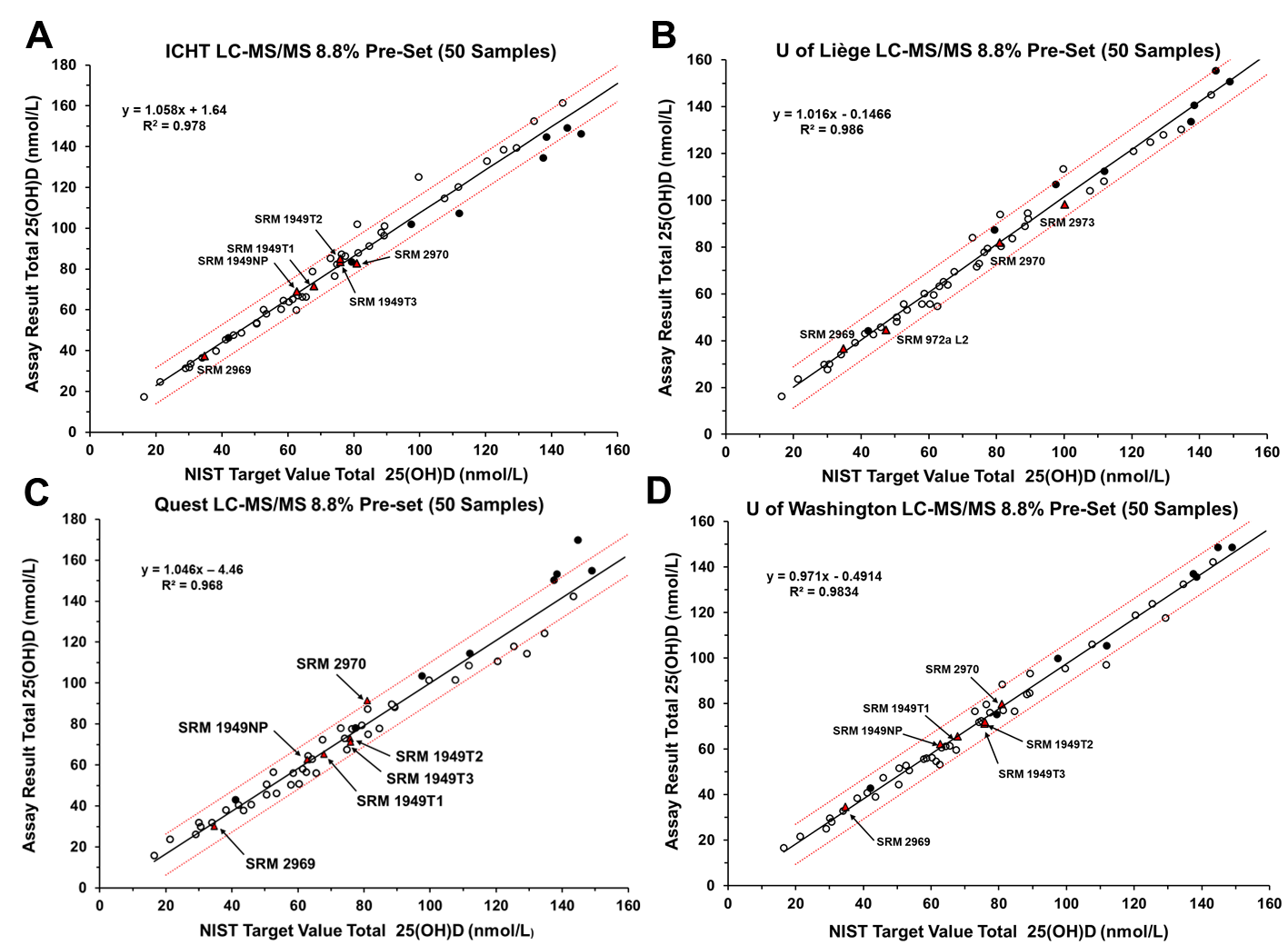


Figure S25.


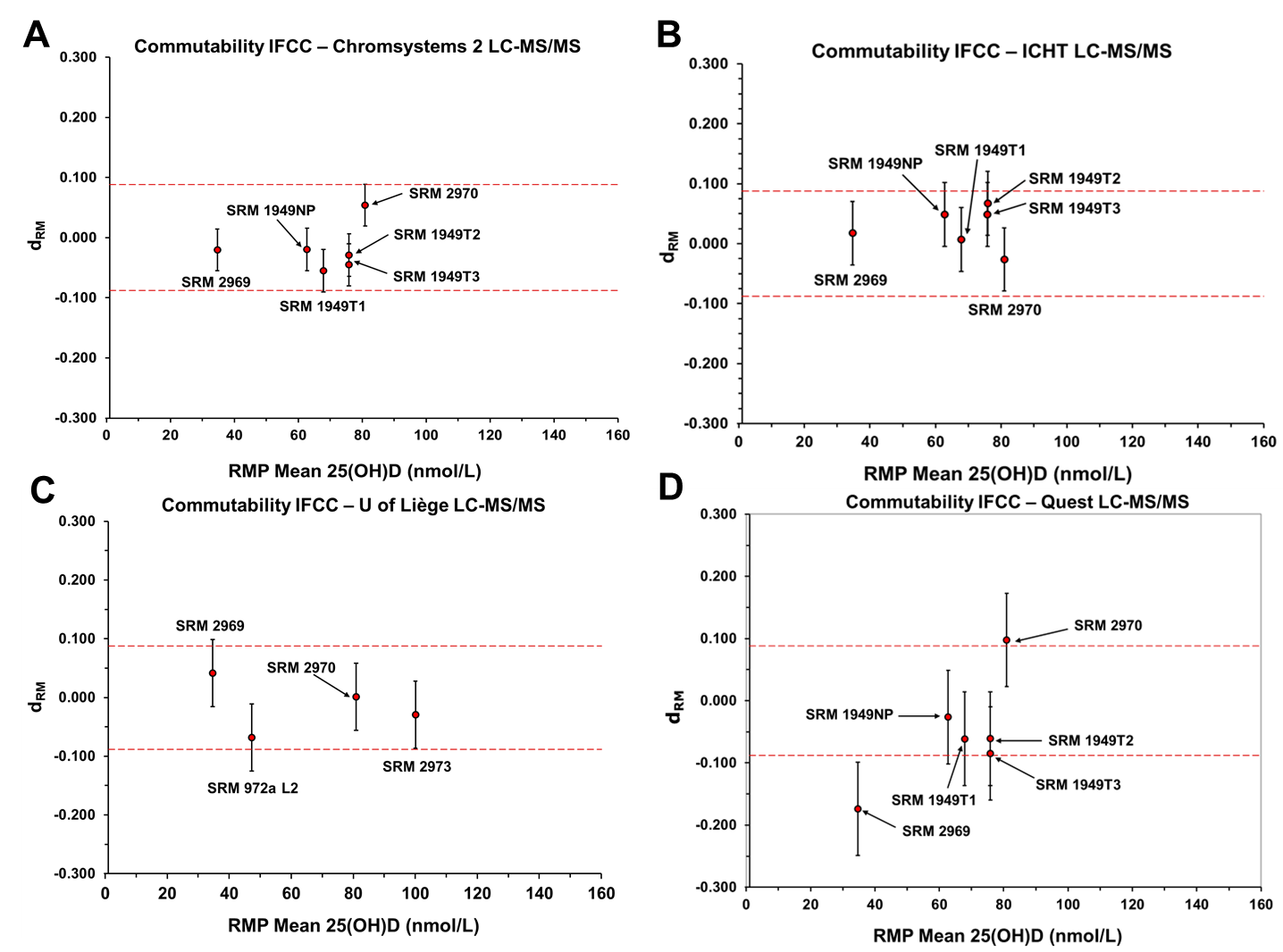
Figure S26.


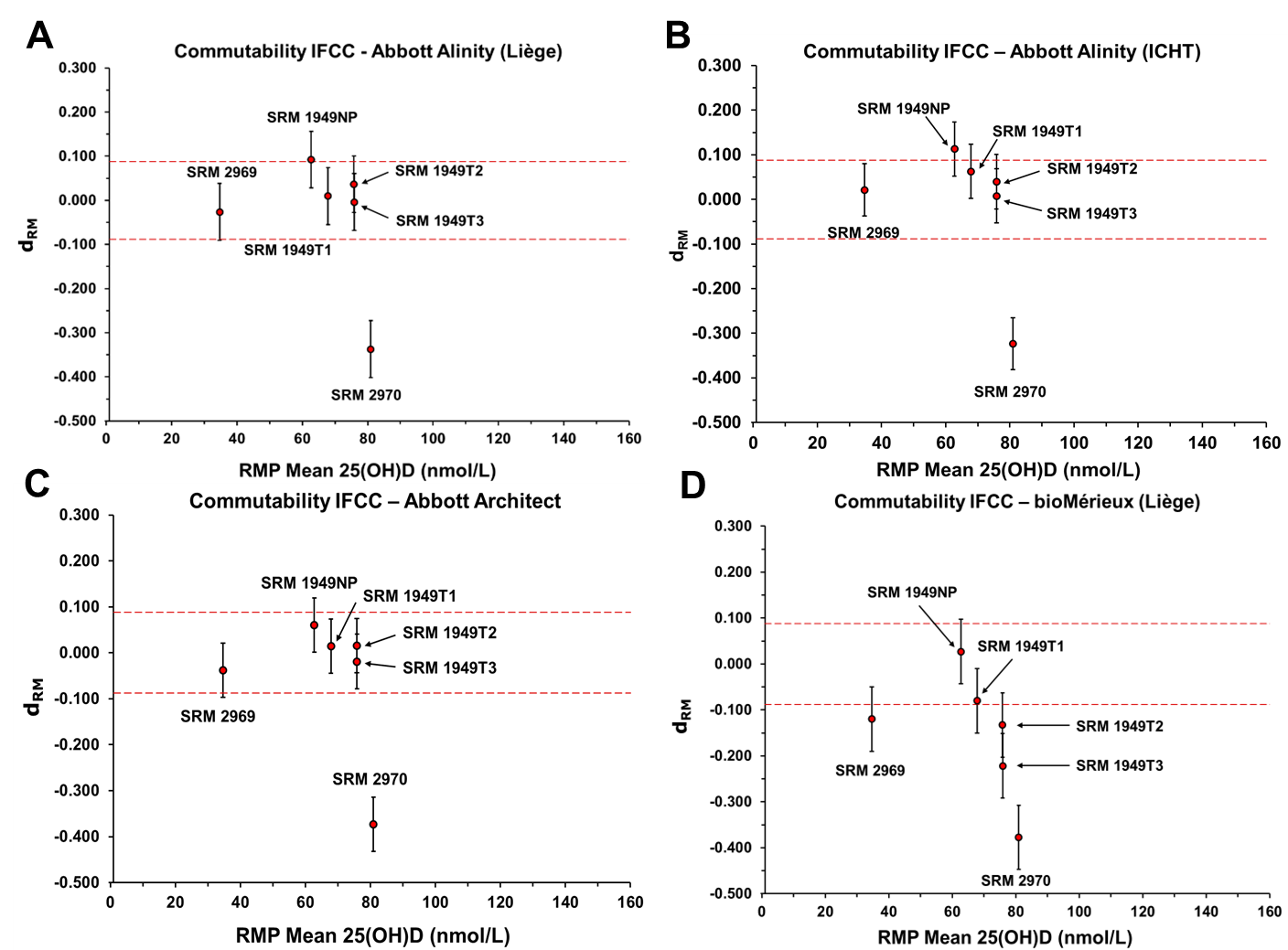


Figure S27.


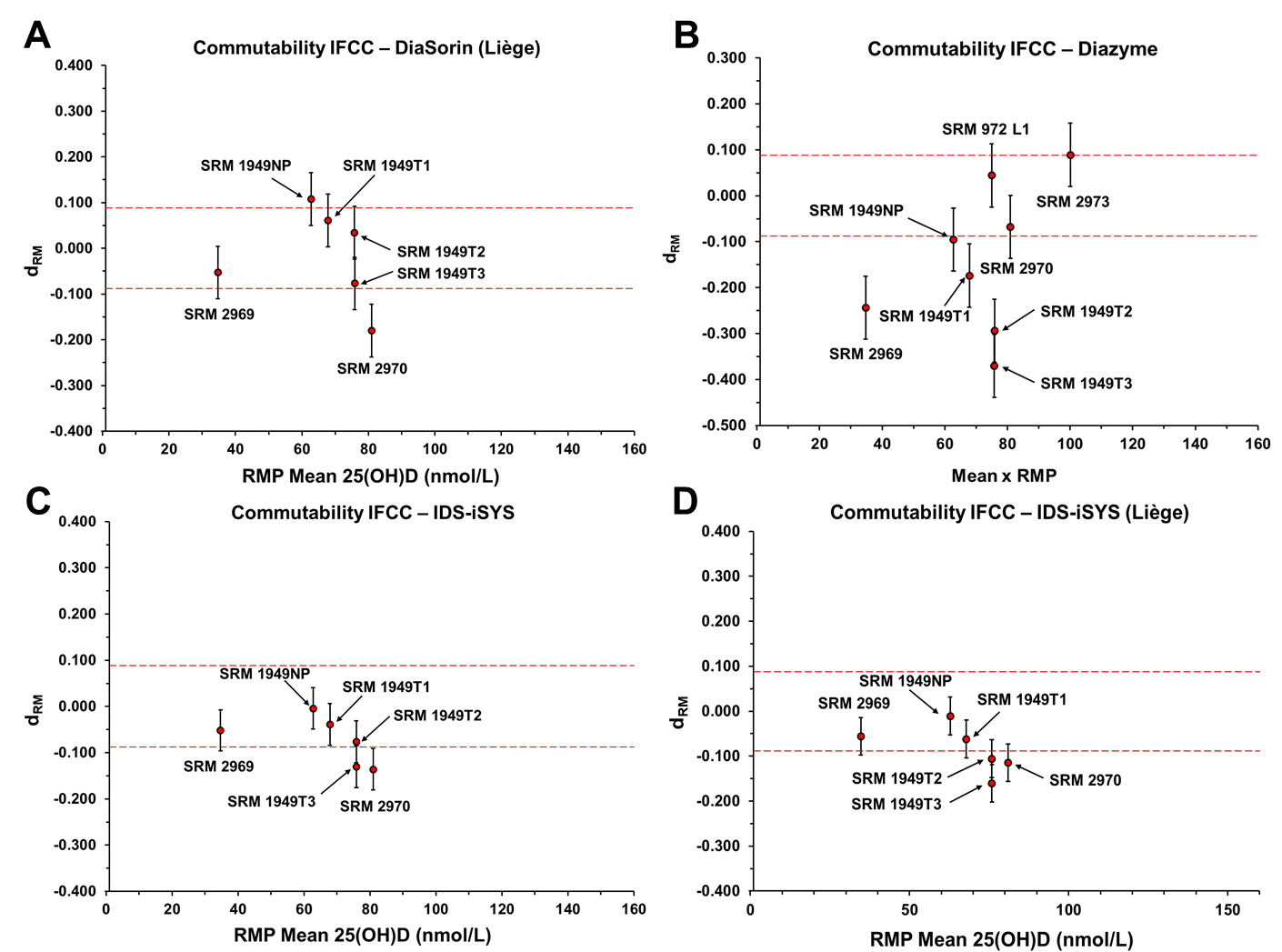


Figure S28.


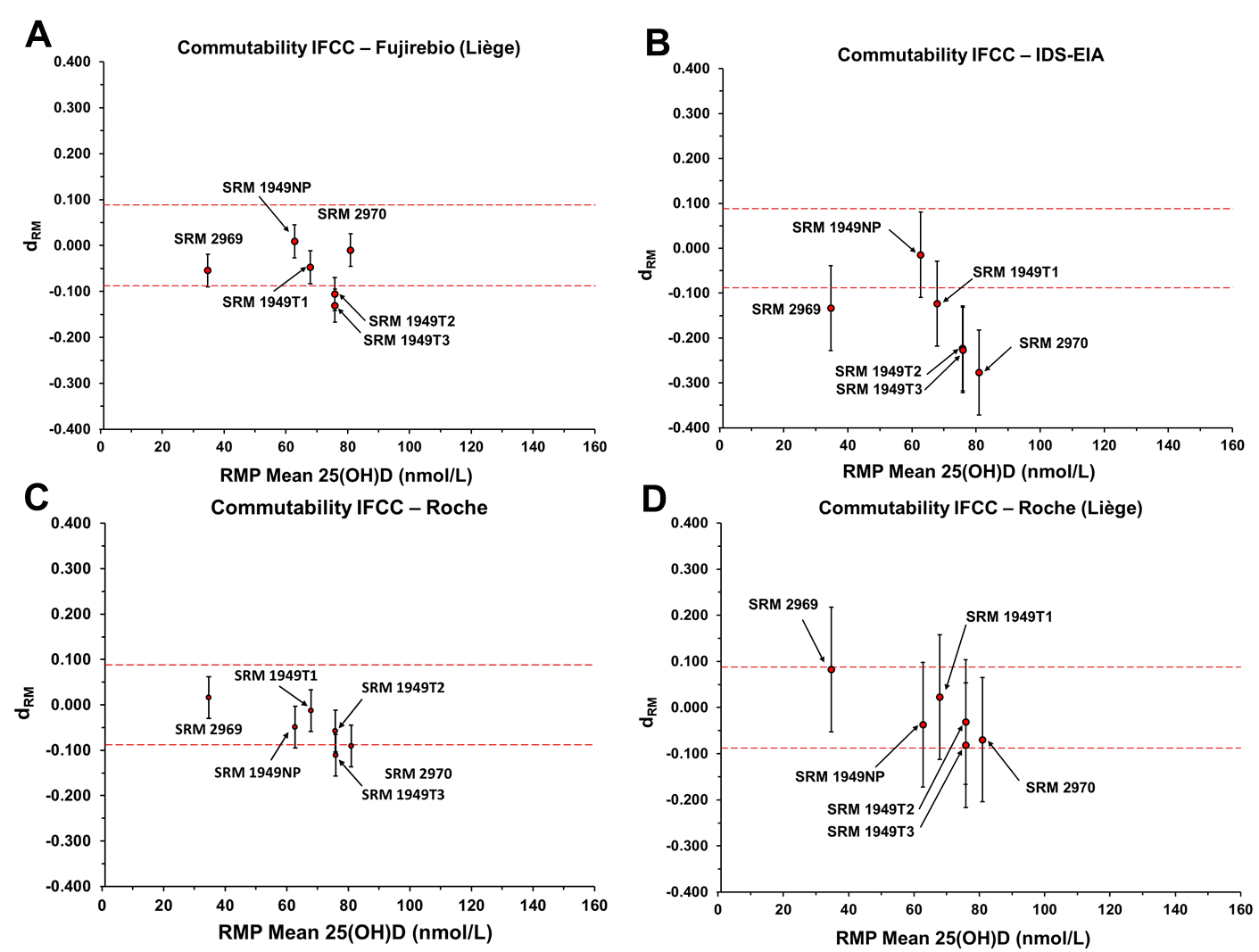


Figure S29.


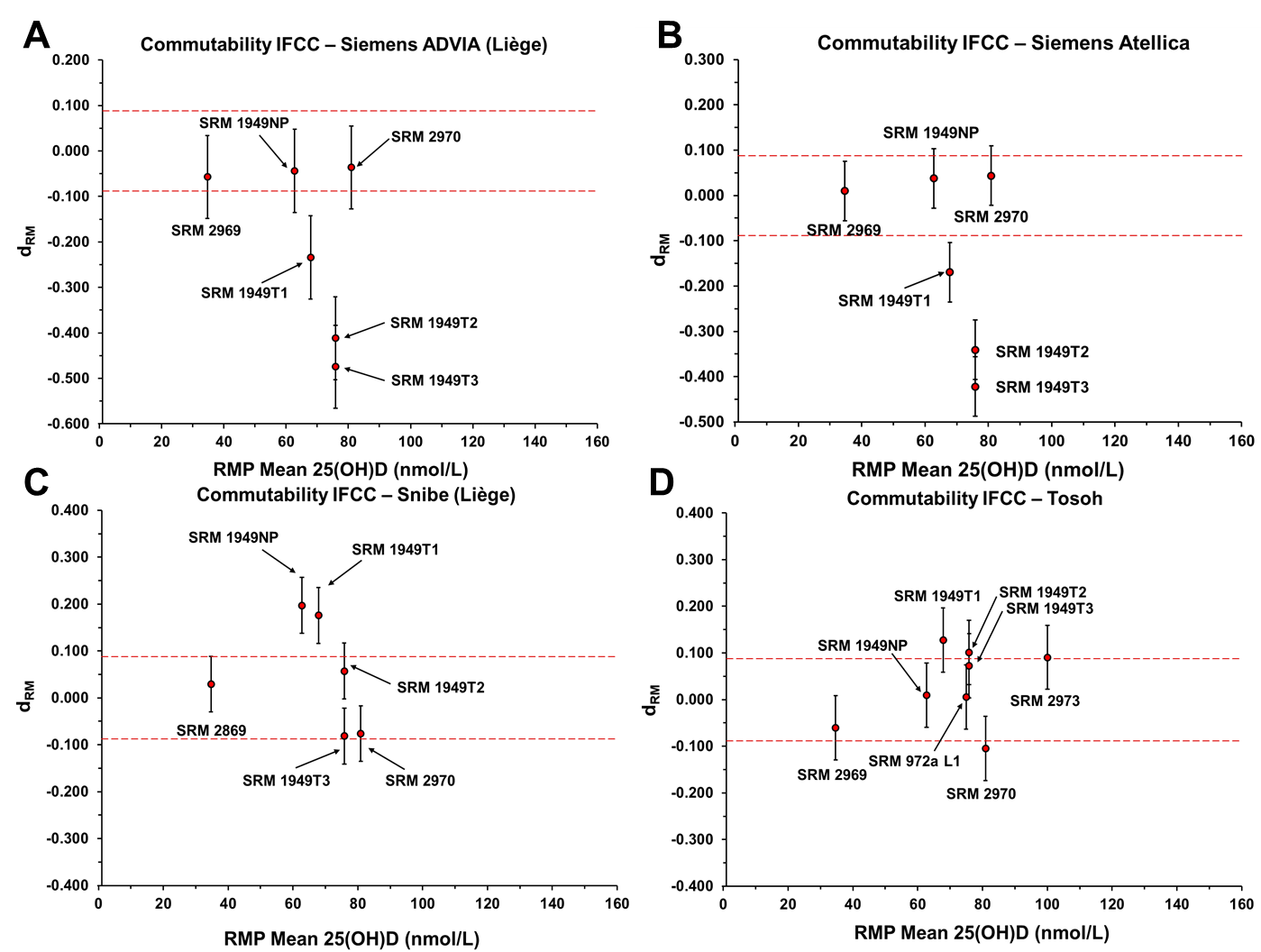


Figure S30.


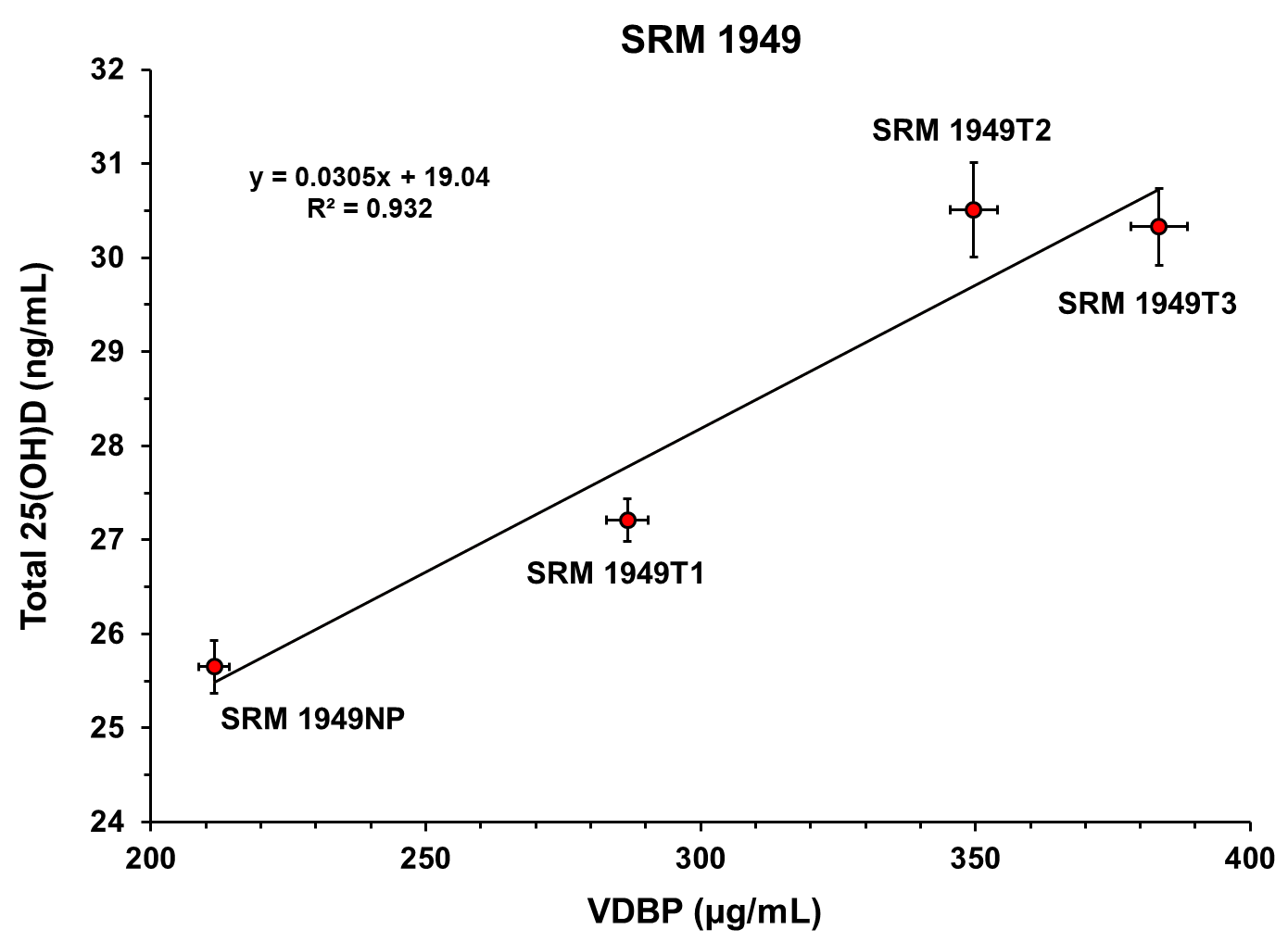

Supplement: Supplementary file 1 — Supplementary file1 (DOCX 7.10 MB) [file 216_2024_5699_MOESM1_ESM.docx]
