# Supplementary material for: Commutability assessment of new standard reference materials (SRMs) for determining serum total 25-hydroxyvitamin D using ligand binding and liquid chromatography–tandem mass spectrometry (LC–MS/MS) assays
Source: Anal Bioanal Chem. 2025 Jan 10;417(12):2539–61. doi: 10.1007/s00216-024-05699-7 (PMC12003064; doi:10.1007/s00216-024-05699-7)
Supplement: Supplementary file 2 — Supplementary file2 (DOCX 184 KB) [file 216_2024_5699_MOESM2_ESM.docx]

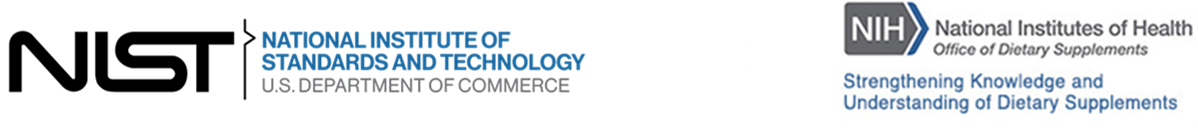


**25(OH)D Commutability Study Protocol 2022**

**(SRM 2969, SRM 2970, and SRM 1949)**

# A Collaborative Effort from the National Institutes of Health (NIH), Office of Dietary Supplements (ODS) and the National Institute of Standards and Technology (NIST)

**Study Contacts:**

Stephen A. Wise, Ph.D., NIH ODS

stephen.wise@nih.gov

Adam J. Kuszak, Ph.D., NIH ODS

adam.kuszak@nih.gov

Johanna E. Camara, Ph.D., NIST

johanna.camara@nist.gov

**INTRODUCTION**

All Certified Reference Materials (CRMs) for clinical diagnostic markers in human serum, which are typically pooled and/or processed, should undergo a commutability assessment to support their equivalent performance to that of patient samples. The National Institutes of Health, Office of Dietary Supplements (NIH ODS) has supported two previous commutability studies to determine the commutability of Standard Reference Materials^®^ (SRMs^®^) and External Quality Assessment (EQA) materials for the determination of total 25(OH)D, with the first study conducted in 2011 (SRM 972a Vitamin D Metabolites in Frozen Human Serum) [1] and the second study conducted in 2016 [SRM 972a and SRM 2973 Vitamin D Metabolites in Frozen Human Serum (High Level] [2]. SRMs are CRMs produced by the National Institute of Standards and Technology (NIST).

Funded in part by NIH ODS, NIST has recently made available three new SRMs for clinical vitamin D metabolite measurements with novel properties:

- SRM 2969 *Vitamin D Metabolites in Frozen Human Serum (Total 25-Hydroxyvitamin D Low Level)* has a lower level of total 25(OH)D (i.e., 13.9 ng/mL) than prior existing SRMs (i.e., 18.9 ng/mL in SRM 972a). The previous commutability studies have not addressed this low level of 25(OH)D with SRMs or EQA samples.
- SRM 2970 *Vitamin D Metabolites in Frozen Human Serum (25-Hydroxyvitamin D_2_ High Level)* has a high endogenous 25(OH)D_2_ concentration, which is higher than prior existing endogenous SRMs (i.e., 56.9 nmol/L vs. 32.0 nmol/L in SRM 972a L3).
- SRM 1949 *Frozen Human Prenatal Serum (Four Levels)* comprises prenatal serum with three levels for the three trimesters of pregnancy and one for non-pregnant women [3], and it has values assigned for 25(OH)D_3_, 25(OH)D_2_, and 3-epi-25(OH)D_3_.

Assessing commutability for SRM 2969 and 2970 is important because materials with low levels of 25(OH)D have not been assessed previously and materials with high levels of 25(OH)D_2_ were shown to be non-commutable for several assays in previous commutabilities studies [2]. SRM 1949 will be tested for commutability to identify any potential problems with its fitness for use in determinations of 25(OH)D.

This document describes the protocol for the 25(OH)D Commutability Study 2022. In this study, we will assess the commutability of the three new SRMs with a total of six levels:

1. SRM 2969 Vitamin D Metabolites in Frozen Human Serum (Total 25-Hydroxyvitamin D Low Level)
2. SRM 2970 Vitamin D Metabolites in Frozen Human Serum (25-Hydroxyvitamin D_2_ High Level)
3. SRM 1949 Frozen Human Prenatal Serum (Four Levels)

In addition, several participants using assays that were not included in the previous commutability study [2], when SRM 972a and SRM 2973 were evaluated, will also incorporate the following SRMs into the analysis protocol to provide a more comprehensive evaluation of commutability for these SRMs:

1. NIST SRM 972a Vitamin D Metabolites in Frozen Human Serum, (Level 1 only)
2. NIST SRM 2973 Vitamin D Metabolites in Frozen Human Serum (High Level)

Recently, the International Federation of Clinical Chemistry (IFCC) proposed a new statistical approach for the assessment of commutability of CRMs and EQA samples since the previous commutability study was conducted in 2017 [4-6]. This commutability study is designed and conducted in a manner to employ the new IFCC statistical approaches for assessment of commutability [5]. Consideration will also be given to the generation of results to allow the assessment of commutability using other historical statistical approaches, thereby providing the clinical community with a case study example to evaluate potential advantages of the new IFCC guidelines.

**PROTOCOL**

Please review the participant protocol in its entirety prior to the start of analysis. Samples will be shipped frozen on dry ice from NIST and should remain frozen at -60 °C or lower until analysis. You will also receive a data reporting template. If there are any questions regarding the protocol or the data reporting template, please contact Stephen Wise (stephen.wise@nih.gov) for clarification.

*Study Materials*

Each participant will receive a panel of 50 single-donor serum samples (DS) obtained according to the Clinical and Laboratory Standards Institute (CLSI) C37-A protocol [7]. These 50 samples cover the range of total 25(OH)D of 20 nmol/L to 150 nmol/L, which is the range commonly observed in the general population.

In addition to the panel of 50 single-donor samples, each participant will also receive three NIST SRMs: (1) SRM 2969, (2) SRM 2970, and (3) SRM 1949 (four levels). The single-donor samples and SRMs used in this study are summarized in Table 1.

**Table 1: Single-Donor and SRMs provided for commutability study**

| **Sample ID** | **Designation**  **in Run Order** | **No. of Vials Provided** | **Approximate Volume in Each Vial (mL)** |
| --- | --- | --- | --- |
| DS01 to DS50 | DS01 to DS50 | 50 | 0.5 |
| SRM 2969 | SRM2969 | 1 | 1.1 |
| SRM 2970 | SRM2970 | 1 | 1.1 |
| SRM 1949NP | SRM1949NP | 1 | 1.8 |
| SRM 1949T1 | SRM1949T1 | 1 | 1.8 |
| SRM 1949T2 | SRM1949T2 | 1 | 1.8 |
| SRM 1949T3 | SRM1949T3 | 1 | 1.8 |

DS = Donor Sample

NP = Non-pregnant

T1 = 1^st^ Trimester

T2 = 2^nd^ Trimester

T3 = 3^rd^ Trimester

**NOTE: If you are running multiple assay platforms or if your analyses will require more sample than is available in single vials of the single-donor and SRM samples, please contact us immediately so that we can provide you with the appropriate quantity of samples (johanna.camara@nist.gov with a copy to stephen.wise@nih.gov).**

*Sample Receipt*

Each participant will receive 1 black cryobox with vials of serum from 50 single donors (0.5 mL each) and 1 white cryobox with SRM 2969, SRM 2970, and SRM 1949. The black cryobox containing the coded single-donor serum samples will be labeled with a 3-digit set number which is unique to each participant. Upon receipt, all samples should be stored frozen (at -60 °C or lower) until analyzed. Each participant will receive an e-mail with the shipping information for tracking, a list of samples with sample IDs, and a data reporting template (Microsoft Excel file: Data Reporting Template 2022 Commutability Study).

***Frozen materials will be shipped on dry ice during the weeks of October 10 and October 17, 2022 by NIST.*** Upon receipt of the samples, participants should assess the shipment for completeness, for samples that are damaged or leaking, and whether samples are still frozen. Please contact Johanna Camara (johanna.camara@nist.gov) within 24 hours of receiving samples to confirm receipt of samples and to report any discrepancies.

*Sample Analysis*

The analysis protocol consists of one run with duplicate measurements in adjacent positions of the single-donor samples and of the six SRM levels distributed in five positions in the run sequence (a total of 160 samples). If the analysis run for the 160 samples requires multiple days, the sample run sequence should be maintained over the analysis period. For the SRMs, five subsamples from each SRM (denoted as A, B, C, D, and E) are measured in duplicate and distributed among the single-donor samples in five different positions. Duplicate measurements are noted in the run order by appending the sample codes with “-1” and “-2” for each DS and SRM sample. Duplicate measurements are to be made from the same sample vial. For laboratories that require a greater volume of serum than is available in a single donor sample vial (0.5 mL), an additional vial will be provided to facilitate the duplicate measurements. The contents of the second vial can be combined with the contents of the first vial prior to analysis or the duplicates can be prepared from the separate vials. Methods requiring sample preparation before the actual measurement need to perform two separate sample preparations. Two injections from the same sample preparation would ***not*** be considered duplicate measurements for the purpose of this study. For those assays that use sampling cups, duplicate measurements should be made from aliquots out of separate cups. ***The specified run order is shown in Appendix A and provided in the data reporting template***.

The vials should be allowed to thaw at room temperature for at least 30 min under subdued light prior to analysis. The contents of the vials should then be gently mixed prior to removal of a test portion for analysis. Precautions should be taken to avoid exposure to strong UV light and direct sunlight.

All participants are responsible for fulfilling their laboratory internal quality control requirements. Participants are requested to provide their quality control data along with study sample data on the data reporting template provided.

*Reporting Results*

***Results should be reported back to NIH ODS by December 16, 2022.*** Reported measurement results and method information should be completed by participants on the provided data reporting template. All available results for total 25(OH)D, 25(OH)D_2_, 25(OH)D_3_, and 3-epi-25(OH)D_3_ should be reported. All sample vial IDs should be recorded in the data reporting template provided. **All donor sample vials have unique IDs. All results should be reported in units of nmol/L with 3 significant digits.**

Please provide the following information about your assay in the data reporting template:

1. Instrument description and measurement technology
2. Assay performance characteristics such as limit of detection and measurement range
3. Lot numbers of reagent(s), calibrators, and controls used in the analysis
4. Measurement results of all samples (nmol/L) including the manufacturer’s controls
5. Concentration range assigned to the manufacturer’s controls

Please e-mail the completed data reporting template to Stephen Wise at NIH ODS (stephen.wise@nih.gov).

**DATA ANALYSIS**

Historically, commutability of SRMs and EQA materials has been assessed following Clinical and Laboratory Standards Institute (CLSI) Guideline EP30-A “Characterization and Qualification of Commutable Reference Materials for Laboratory Medicine: Approved Guideline” [8]. In brief, a mathematical relationship between the laboratory results and the Reference Measurement Procedure (RMP) results is established using the donor samples. Then, results from the SRM samples are assessed as to whether they fit in this mathematical relationship (i.e., whether they fall within the 95% prediction interval of the regression line). Materials fitting in this mathematical relationship would be considered commutable.

For this study, commutability assessment of the SRMs will be performed following recent guidelines published by the International Federation of Clinical Chemistry (IFCC) [4-6]. This approach is based on the difference in bias between an SRM and clinical samples (CSs) measured using two different measurement procedures (MPs). In this study, one of the MPs is the NIST RMP based on isotope dilution liquid chromatography – tandem mass spectrometry (ID LC-MS/MS) for the determination of 25(OH)D_2_ and 25(OH)D_3_ [9]. This difference in bias is compared with a criterion based on a medically relevant difference between the SRM and CS results to make a conclusion regarding commutability. The assessment is based on an error model that allows estimation of various random and systematic sources of error, including those from sample-specific effects of interfering substances. An advantage of this approach is that the difference in bias between the SRM and the average bias of CSs at the concentration (i.e., amount of substance present or quantity value) of the SRM is determined and its uncertainty estimated. An SRM is considered fit for purpose for those MPs for which commutability is demonstrated [6].

Analysis of the data for the commutability assessment will be performed at NIH ODS. After completion of the data analysis, a participant report will be generated and sent to each participant in which the NIST-assigned values for each donor sample will be compared with the participant assay results and the commutability of the SRMs will be evaluated using the participant assay results. Upon completion of the detailed assessment of commutability of the SRMs using all assays, a report and/or manuscript describing these results will be provided to the participants for review and comment. Commutability assessment using the IFCC approach may be compared with results using the historical CLSI approach. Results obtained from this study, including participant and assay manufacturer information, will be submitted for publication in a peer-reviewed journal.

**SAFETY**

ALL SERUM MATERIALS PROVIDED ARE INTENDED FOR LABORATORY USE ONLY. THESE ARE HUMAN-SOURCE MATERIALS. HANDLE PRODUCTS AS BIOHAZARDOUS MATERIALS CAPABLE OF TRANSMITTING INFECTIOUS DISEASE. The supplier of the serum has reported that each donor unit of serum used in the preparation of this product has been tested by an FDA-approved method and found non-reactive/negative for hepatitis B surface antigen (HbsAg), human immunodeficiency (HIV) 1 and 2 antibodies, and hepatitis C virus (HCV). However, no known test method can offer complete assurance that hepatitis B virus, hepatitis C virus, HIV, or other infectious agents are absent from this material. Accordingly, this human blood-based product should be handled at the Biosafety Level 2 or higher as recommended for any POTENTIALLY INFECTIOUS HUMAN SERUM OR BLOOD SPECIMEN in the Centers for Disease Control/National Institutes of Health Manual [10].

**REFERENCES**

[1] K.W. Phinney, C.T. Sempos, S.S.C. Tai, J.E. Camara, S.A. Wise, J.H. Eckfeldt, A.N. Hoofnagle, G.D. Carter, J. Jones, G.L. Myers, R. Durazo-Arvizu, W.G. Miller, L.M. Bachmann, I.S. Young, J. Pettit, G. Caldwell, A. Liu, S.P.J. Brooks, K. Sarafin, M. Thamm, G.S.M. Mensink, M. Busch, M. Rabenberg, K.D. Cashman, M. Kiely, K. Galvin, J.Y. Zhang, M. Kinsella, K. Oh, S.W. Lee, C.L. Jung, L. Cox, G. Goldberg, K. Guberg, S. Meadows, A. Prentice, Baseline assessment of 25-hydroxyvitamin D reference material and proficiency testing/external quality assurance material commutability: A Vitamin D Standardization Program Study, J. AOAC Int., 100:1288-1293 (2017).

[2] J.E. Camara, S.A. Wise, A.N. Hoofnagle, E.L. Williams, G.D. Carter, J. Jones, C.Q. Burdette, G. Hahm, F. Nalin, A.J. Kuszak, E. Cavalier, P. Lukas, R. Durazo-Arvizu, C. Popp, C. Beckert, C. Schultess, G. Van Slooten, C. Tourneur, C. Pease, K. Kaul, A. Villarreal, B. Emanuelli, A. Maggio, F. Ivison, R. Fischer, J.M.W. van den Ouweland, C.S. Ho, E.W.K. Law, J.-N. Simard, R. Gonthier, B. Holmquist, M.C. Batista, H. Pham, A. Bennett, S. Meadows, L. Cox, E. Jansen, D.A. Khan, K. Robyak, M.H. Creer, M. Kilbane, J. Freeman, N. Parker, V. Chen, R. Fitzgerald, S. Mushtaq, M.W. Clarke, N. Breen, C. Simpson, C.T. Sempos, Assessment of serum total 25-hydroxyvitamin D assay commutability of Standard Reference Materials, College of American Pathologists Accuracy-Based Vitamin D (ABVD) Scheme and Vitamin D External Quality Assessment Scheme (DEQAS) materials: Vitamin D Standardization Program (VDSP) commutability study 2, Anal. Bioanal. Chem., 413:5067-5084 (2021)

[3] A.S.P. Boggs, L.E. Kilpatrick, C.Q. Burdette, D.S. Tevis, Z.A. Fultz, M.A. Nelson, J.M. Jarrett, J.V. Kemp, R.J. Singh, S.K.G. Grebe, S.A. Wise, B.L. Kassim, S.E. Long, Development of a pregnancy-specific reference material for thyroid biomarkers, vitamin D, and nutritional trace elements in serum, Clin. Chem. Lab. Med., 59:671-679 (2021).

[4] W.G. Miller, H. Schimmel, R. Rej, N. Greenberg, F. Ceriotti, C. Burns, J.R. Budd, C. Weykamp, V. Delatour, G. Nilsson, F. MacKenzie, M. Panteghini, T. Keller, J.E. Camara, I. Zegers, H.W. Vesper, Commutability, IFCC working group recommendations for assessing commutability Part 1: General experimental design, Clin. Chem., 64:447-454 (2018).

[5] G. Nilsson, J.R. Budd, N. Greenberg, V. Delatour, R. Rej, M. Panteghini, F. Ceriotti, H. Schimmel, C. Weykamp, T. Keller, J.E. Camara, C. Burns, H.W. Vesper, F. MacKenzie, W.G. Miller, IFCC working group recommendations for assessing commutability Part 2: Using the difference in bias between a reference material and clinical samples, Clin. Chem., 64:455-464 (2018).

[6] J.R. Budd, C. Weykamp, R. Rej, F. MacKenzie, F. Ceriotti, N. Greenberg, J.E. Camara, H. Schimmel, H.W. Vesper, T. Keller, V. Delatour, M. Panteghini, C. Burns, W.G. Miller, IFCC working group recommendations for assessing commutability Part 3: Using the calibration effectiveness of a reference material, Clin. Chem., 64:465-474 (2018).

[7] Clinical and Laboratory Standards Institute (CLSI), Preparation and Validation of Commutable Frozen Human Serum Pools as Secondary Reference Materials for Cholesterol Measurement Procedures. CLSI Document C37-A. Clinical and Laboratory Standards Institute, Wayne, PA (1999)

[8] Clinical and Laboratory Standards Institute (CLSI) Characterization and Qualification of Commutable Reference Materials for Laboratory Medicine. CLSI document EP30A. Clinical and Laboratory Standards Institute, Wayne, PA (2010)

[9] S.S-C. Tai, M. Bedner, K.W. Phinney Development of a candidate reference measurement procedure for the determination of 25-hydroxyvitamin D_3_ and 25-hydroxyvitamin D_2_ in human serum using isotope-dilution liquid chromatography-tandem mass spectrometry. Anal Chem 82 (5):1942-1948 (2010).

[10] CDC/NIH; Biosafety in Microbiological and Biomedical Laboratories; 6th ed.; Richardson, J.; Barkley, W.E.; Richmond, J.; McKinney, R.W.; Eds.; U.S. Department of Health and Human Services, Public Health Service, Centers for Disease Control and Prevention and National Institutes of Health; US Government Printing Office: Washington, DC (2007); available at http://www.cdc.gov/labs/BMBL.html (accessed August 5, 2022).

**APPENDIX A: ANALYSIS RUN ORDER TO BE USED ON ONE DAY**

| Run order | Sample code | Run order | Sample code | Run order | Sample code | Run order | Sample code |
| --- | --- | --- | --- | --- | --- | --- | --- |
| 1 | DS1-1 | 41 | SRM2970B-1 | 81 | DS26-1 | 121 | DS39-1 |
| 2 | DS1-2 | 42 | SRM2970B-2 | 82 | DS26-2 | 122 | DS39-2 |
| 3 | DS2-1 | 43 | DS14-1 | 83 | DS27-1 | 123 | SRM1949T2D-1 |
| 4 | DS2-2 | 44 | DS14-2 | 84 | DS27-2 | 124 | SRM1949T2D-2 |
| 5 | SRM2969A-1 | 45 | DS15-1 | 85 | SRM1949T1C-1 | 125 | DS40-1 |
| 6 | SRM2969A-2 | 46 | DS15-2 | 86 | SRM1949T1C-2 | 126 | DS40-2 |
| 7 | DS3-1 | 47 | SRM1949NPB-1 | 87 | DS28-1 | 127 | SRM1949T3D-1 |
| 8 | DS3-2 | 48 | SRM1949NPB-2 | 88 | DS28-2 | 128 | SRM1949T3D-2 |
| 9 | SRM2970A-1 | 49 | DS16-1 | 89 | DS29-1 | 129 | DS41-1 |
| 10 | SRM2970A-2 | 50 | DS16-2 | 90 | DS29-2 | 130 | DS41-2 |
| 11 | DS4-1 | 51 | DS17-1 | 91 | SRM1949T2C-1 | 131 | DS42-1 |
| 12 | DS4-2 | 52 | DS17-2 | 92 | SRM1949T2C-2 | 132 | DS42-2 |
| 13 | DS5-1 | 53 | SRM1949T1B-1 | 93 | DS30-1 | 133 | SRM2969E-1 |
| 14 | DS5-2 | 54 | SRM1949T1B-2 | 94 | DS30-2 | 134 | SRM2969E-2 |
| 15 | SRM1949NPA-1 | 55 | DS18-1 | 95 | SRM1949T3C-1 | 135 | DS43-1 |
| 16 | SRM1949NPA-2 | 56 | DS18-2 | 96 | SRM1949T3C-2 | 136 | DS43-2 |
| 17 | DS6-1 | 57 | DS19-1 | 97 | DS31-1 | 137 | SRM2970E-1 |
| 18 | DS6-2 | 58 | DS19-2 | 98 | DS31-2 | 138 | SRM2970E-2 |
| 19 | DS7-1 | 59 | SRM1949T2B-1 | 99 | DS32-1 | 139 | DS44-1 |
| 20 | DS7-2 | 60 | SRM1949T2B-2 | 100 | DS32-2 | 140 | DS44-2 |
| 21 | SRM1949T1A-1 | 61 | DS20-1 | 101 | SRM2969D-1 | 141 | DS45-1 |
| 22 | SRM1949T1A-2 | 62 | DS20-2 | 102 | SRM2969D-2 | 142 | DS45-2 |
| 23 | DS8-1 | 63 | SRM1949T3B-1 | 103 | DS33-1 | 143 | SRM1949NPE-1 |
| 24 | DS8-2 | 64 | SRM1949T3B-2 | 104 | DS33-2 | 144 | SRM1949NPE-2 |
| 25 | DS9-1 | 65 | DS21-1 | 105 | SRM2970D-1 | 145 | DS46-1 |
| 26 | DS9-2 | 66 | DS21-2 | 106 | SRM2970D-2 | 146 | DS46-2 |
| 27 | SRM1949T2A-1 | 67 | DS22-1 | 107 | DS34-1 | 147 | DS47-1 |
| 28 | SRM1949T2A-2 | 68 | DS22-2 | 108 | DS34-2 | 148 | DS47-2 |
| 29 | DS10-1 | 69 | SRM2969C-1 | 109 | DS35-1 | 149 | SRM1949T1E-1 |
| 30 | DS10-2 | 70 | SRM2969C-2 | 110 | DS35-2 | 150 | SRM1949T1E-2 |
| 31 | SRM1949T3A-1 | 71 | DS23-1 | 111 | SRM1949NPD-1 | 151 | DS48-1 |
| 32 | SRM1949T3A-2 | 72 | DS23-2 | 112 | SRM1949NPD-2 | 152 | DS48-2 |
| 33 | DS11-1 | 73 | SRM2970C-1 | 113 | DS36-1 | 153 | DS49-1 |
| 34 | DS11-2 | 74 | SRM2970C-2 | 114 | DS36-2 | 154 | DS49-2 |
| 35 | DS12-1 | 75 | DS24-1 | 115 | DS37-1 | 155 | SRM1949T2E-1 |
| 36 | DS12-2 | 76 | DS24-2 | 116 | DS37-2 | 156 | SRM1949T2E-2 |
| 37 | SRM2969B-1 | 77 | DS25-1 | 117 | SRM1949T1D-1 | 157 | DS50-1 |
| 38 | SRM2969B-2 | 78 | DS25-2 | 118 | SRM1949T1D-2 | 158 | DS50-2 |
| 39 | DS13-1 | 79 | SRM1949NPC-1 | 119 | DS38-1 | 159 | SRM1949T3E-1 |
| 40 | DS13-2 | 80 | SRM1949NPC-2 | 120 | DS38-2 | 160 | SRM1949T3E-2 |
